# Supplementary material for: Connectivity guided theta burst transcranial magnetic stimulation versus repetitive transcranial magnetic stimulation for treatment-resistant moderate to severe depression: study protocol for a randomised double-blind controlled trial (BRIGhTMIND)
Source: BMJ Open. 2020 Jul 7;10(7):e038430. doi: 10.1136/bmjopen-2020-038430 (PMC7342821; doi:10.1136/bmjopen-2020-038430)
Supplement: Supplementary data [file bmjopen-2020-038430supp001.pdf]

Appendix 1: Assessment of degree of treatment resistance.

The definition and assessment of the degree of treatment resistance depression (TRD) in the BRIGhTMIND study is operationalised using the Massachusetts General Hospital (MGH) TRD scoring system (Fava et al, 2003). This assesses the number of previous biological treatments for depression, given as an adequate treatment trial, in the current episode of depression. The use of psychological treatments is not assessed by this tool because it can be challenging to obtain reliable accounts of structured psychotherapy from participant's accounts (Lobban et al, 2017). To date, the relationship between non-response to psychotherapy and response to TMS has not been investigated. This is in contrast to evidence that the degree of treatment resistance to biological treatments may moderate treatment outcome to TMS (Lee et al, 2012). The use of, and response to, psychological therapy is recorded separately to the MGH TRD score in the BRIGhTMIND study for use as an exploratory variable in relation to predictors of response.

The MGH TRD score is based on one point being scored for every different antidepressant prescribed at the minimum effective dose for a minimum of 6 weeks, without response. An extra 0.5 points is scored if the trial has been at least 10 weeks and if the dose of the antidepressant has been optimised to a defined level. A further 0.5 can be scored if the antidepressant has been augmented by a second drug. Any course of electroconvulsive therapy lasting a minimum of 4 treatment sessions is given a score of 3 points.

The MGH TRD scoring tool used in BRIGhTMIND is an updated version of that published by Fava et al. in 2003, with medications not available at that time added and ones that are no longer, or where never, available in the UK removed (see supplementary table 1). Specifically, the tricyclic antidepressants amoxapine, desipramine, maprotiline and protriptyline, and the "other" antidepressant nefazadone, that were included in the scoring table in Fava et al. (2003), were removed. The tricyclic antidepressants dosulepin (75 and 150mg) lofepramine (140 and 210mg), monoamine oxidase inhibitor moclobemide (300 and 600mg) and "other" antidepressants reboxetine (8 and 12mg), agomelatine (25 and 50mg) and vortioxetine (10 and 20mg) were added with minimum and optimised doses indicated in brackets. These doses were based on the various drugs' Summary of Product Characteristics (SPC) and consensus of the clinicians involved in the setup of BRIGhTMIND.

In the scoring table published by Fava et al. (2003), the definition of which drugs could be counted towards augmenting an antidepressant is vague, simply presenting examples: "e.g., buspirone (Buspar), lithium, psychostimulants such as methylphenidate (Ritalin), atypical antipsychotics such as olanzapine (Zyprexa). It was decided that more precise guidance should be provided. The drugs included are based on consensus of BRIGhTMIND clinicians and British Association for Psychopharmacology (BAP) Guidelines (Cleare et al, 2015). All first and second line BAP options were included (lithium, aripiprazole, quetiapine, risperidone, olanzapine and tri-iodothyronine (T3)). BAP guidelines also include mirtazapine augmentation of another antidepressant. Because of the diverse range of antidepressant combinations sometimes used in clinical practice, "mirtazapine" was broadened to "any second antidepressant". The two remaining drugs specifically mentioned by Fava et al. (2003), buspirone and methylphenidate were retained (i.e. all drugs specifically mentioned

by Fava et al. are included). Two extra drugs were then added by consensus on the basis of awareness of their use in the centres involved in the BRIGhTMIND study: modafinil and pramipexole. These both have some RCT evidence supporting their use as augmentation agents (Goss et al. 2013; Tundo et al. 2019).

In BRIGhTMIND each patient is scored on the adapted MGH TRD scoring system. The inclusion criterion for the RCT is a score of 2 points or more. This is broadly in line with the conventional definition of TRD as being failure to respond to two adequate courses of different antidepressants (Brown et al. 2019): failure to respond to two different antidepressants prescribed at the minimum dose for 6 weeks each would score 2 X 1 point. However, it should be noted that dose optimisation and a treatment trial of a single antidepressant could lead to the same score (1 + 0.5 + 0.5 points).

Scoring the MGD TRD scale is dependent on having information regarding the patient's past treatments during this current episode. In BRIGhTMIND, this is established through a combination of interview with the patient and examination of primary and secondary care case notes. Patients are interviewed using timeline follow back techniques to identify when the current episode of depression started. However, the MGH TRD scores are very susceptible to error due to mis-remembered details and inadequate, inaccurate or inaccessible case notes. Rather than focusing on the specific MGH TRD score, patients in BRIGhTMIND are allocated to three categories of degree of treatment resistance: low, medium and high. These are defined on the basis of data collected in a previous RCT in patients with TRD, the ADD study (McAllister-Williams et al. 2016), by scores of 2-3.5, 4-6 and ≥6.5 respectively. If there is any concern that there is incomplete data on past treatments, then a patient is allocated to the high resistance group.

The specific guidance on using the MGH TRD scoring system in BRIGhTMIND is as follows:

1. The scale assesses the degree of treatment resistance in the CURRENT EPISODE. For some patients with long histories of depression it can be difficult to determine the beginning of an episode. Count a new episode of depression from the end of any period of substantially better mood for a minimum of 2 months.
2. Confirm if ANY antidepressants have been taken (not just prescribed) in the current episode. If not, then the patient is excluded.
3. If the participant has taken any antidepressants in the current episode then collect information regarding WHAT has been taken (all psychotropics), at what DOSE and for how LONG at the minimum dose or greater. Then, using supplementary table 1:
    a. in column A, tick an antidepressant the patient has taken at the minimum dose for at least 6 weeks during THIS episode of depression.
    b. for antidepressants ticked in column A, put another tick in column B if the treatment continued for at least 10 weeks.
    c. tick column C if the patient has taken the drug at a dose equal to or greater than the maximum dosage listed for that medication. *(There is no extra score for doses higher than the maximum)*
    d. If the patient has been prescribed any of the drugs listed here (taken for at least 4 weeks) during the same time period to boost the antidepressant effect, write the name in column D.

i. **NB** – if an antidepressant combination is used, then only score for one of these with the second antidepressant being the augmentation agent. For example if a patient on venlafaxine has mirtazapine added, put a tick in the venlafaxine row and write ‘mirtazapine’ in Column D. Don’t tick the Mirtazapine row (unless this was also used in monotherapy).
ii. **NB** – augmentation agents should in theory be used at minimum effective doses. However, there is a lack of consensus as to what these should be. If in any doubt seek a view of the local Principal Investigators or Chief Investigator.

4. Add the scores:
   a. For each antidepressant add a score to column E. This is +1 for the antidepressant, +0.5 if used for at least 10 weeks (at minimum dose or above), +0.5 if the Maximum dose or greater was used and +0.5 for EACH augmentation agent used.
   b. If the patient has received ECT in this episode, then add 3 into column E on the ECT row at the bottom
   c. Calculate the total MGH TRD score adding up all scores in column E.

Supplementary table 1

Adapted Massachusetts General Hospitals Staging Method to classify treatment resistant depression for the BRIGhTMIND Study.

Has the patient received any treatment with ANTIDEPRESSANT medications since the beginning of THIS CURRENT episode or period of depression?

1 = Yes 2 = No ☐ If NO – Exclude from Study

If Yes

1) On the Grid below in column A, tick an antidepressants the patient has taken at the minimum dose for at least 6 weeks during THIS episode of depression.
2) For antidepressants ticked in column A, put another tick in column B if the treatment continued for at least 10 weeks.
3) Tick column C if the patient has taken the drug at a dose equal to or greater than the maximum dosage listed for that medication. *(There is no extra score for doses higher than the maximum.)*
4) If the patient has been prescribed any of the drugs listed here during the same time period to boost the antidepressant effect, write the name in column D, *Amisulpride, Aripiprazole, Buspirone, Lithium, Methylphenidate, Modafinil, Olanzapine, Pramipexole, Quetiapine, Risperidone, triiodothyronine (T3), and any second Antidepressant.*

If ticked score			1	0.5		0.5	0.5	
			A	B		C	D	
	**Generic name**	**Min dose (mg/day)**	**At least 6 wks**	**At least 10 wks**	**Max dose (mg/day)**	**Equal or greater**	**Name of drug added to augment this antidepressant**	**Score**
**Tricyclic antidepressants**	Doxepin	150			250			
	Clomipramine	150			250			
	Amitriptyline	150			250			
	Nortriptyline	75			125			
	Trimipramine	150			250			
	Imipramine	150			250			
	Dosulepin	75			150			
	Lofepramine	140			210			
**MAOIs**	Isocarboxazid	30			60			
	Phenelzine	45			90			
	Tranylcypromine	30			60			
	Moclobemide	300			600			

**SSRIs**	Fluvoxamine	50			150			
	Paroxetine	20			60			
	Fluoxetine	20			60			
	Sertraline	50			150			
	Citalopram	20			60			
	Escitalopram	10			30			
**SNRI**	Venlafaxine	125			250			
	Duloxetine	60			100			
**Other**	Trazodone	300			600			
	Bupropion	300			450			
	Mirtazapine	15			45			
	Reboxetine	8			12			
	Agomelatin	25			50			
	Vortioxetine	10			20			
Did the patient receive electroconvulsive treatment (ECT) during the current episode (Score 3)?								
							Total MGH score	

Appendix 2 Studies on Cognition, depression and effective connectivity

Background

Depression is associated with marked impairments in attention and executive function (Austin et al., 2001; McDermott and Ebmeier, 2009). Part of these impairments could be explained by increased intraindividual variability (IIV) in attentional performance – patients with depression show a skewed distribution of response times on sustained attention tasks, with an increased frequency of slow responses consistent with attentional lapses (Gallagher et al., 2015). This can be analysed by decomposing the distribution of response times into a Gaussian component and an exponential component (Schmiedek et al., 2007) – Gallagher et al. (2015) found the time constant of the exponential component to be a more sensitive measure for differentiating patients from controls than simple mean reaction times.

It may be that attentional lapses reflect interference from brain areas involved in internally directed mental activity and rumination (the so-called “default mode network” of areas, DMN) on areas involved in executive function (the “frontoparietal network” of areas, FPN). Previous work has found that executive function in non-clinical populations is related to the degree of anticorrelation between the activities of the FPN and DMN (Kelly et al., 2008; Keller et al., 2015), suggesting that the ability to separate the activities of these networks may be critical to task performance. One of the key findings of a meta-analysis of resting-state fMRI studies in patients with depression was an increase in connectivity between the FPN and the DMN, suggesting a breakdown of this separability (Kaiser et al., 2015).

It is unclear the extent to which attentional and executive impairments are independent components of the pathophysiology of depression or a secondary consequence of impairments in mood (Bora et al., 2013; Rock et al., 2014). Independence could be demonstrated in a dissociation between improvements in mood and improvements in cognition during treatment. Cheng et al. (2016) provide preliminary evidence for such a dissociation with a selective improvement in executive function in a group receiving intermittent TBS compared to those receiving continuous TBS or a combination of intermittent and continuous TBS.

Methods

Embedded within the BRIGhTMIND study, we will explore correlations between IIV on the THINC-it battery of cognitive tests (measured with the method of Gallagher et al., 2015) and resting-state functional connectivity between the FPN and the DMN. Separate correlations using baseline measures of each variable and using change in each variable from baseline to 16 weeks will be calculated. Analyses will be performed without unblinding TMS treatment allocation group. We predict a positive correlation between IIV and FPN-DMN functional connectivity at baseline, and a positive correlation between change in IIV and change in FPN-DMN connectivity. We will explore the mediating effects of low mood (measured with the QIDS-SR, Rush et al., 2013), and improvements in mood, on these relationships. Additionally, we will examine the longitudinal relationship between mood and IIV in a within-subject design using unblinded QIDS-SR and THINC-it data, to ascertain whether increased IIV is a state or trait property of depression.

References

Austin MP, Mitchell P, Goodwin GM. Cognitive deficits in depression: possible implications for functional neuropathology. *Br J Psychiatry* 2001; **178**:200-6.

Bora E, Harrison BJ, Yücel M, et al. Neurocognitive intra-individual variability in mood disorders: effects on attentional response time distributions. *Psychol Med* 2015; **45**: 2985-97.

Brown S, Rittenbach K, Cheung S, McKean G, MacMaster FP, Clement F. Current and Common Definitions of Treatment-Resistant Depression: Findings from a Systematic Review and Qualitative Interviews. *Can. J. Psychiatry* 2019; **64**: 380–387. https://doi.org/10.1177/0706743719828965

Cheng C-M, Juan C-H, Chen M-H, et al. Different forms of prefrontal theta burst stimulation for executive function of medication-resistant depression: evidence from a randomized sham-controlled study. *Prog Neuro-Psychopharmacol Biol Psychiatry* 2016; **66**: 35-40.

Cleare A, Pariante C, Young A, et al. Evidenced-based guidelines for treating depressive disorders with antidepressants: A revision of the 2008 British Association Psychopharmacology guidelines. J Psychopharmacol 2015;29:459-525.

Fava M. Diagnosis and definition of treatment-resistant depression. *Biol Psychiatry* 2003;**53**:649–59. doi:10.1016/S0006-3223(03)00231-2.

Gallagher P, Nilsson J, Finkelmeyer A et al. Neurocognitive intra-individual variability in mood disorders: effects on attentional response time distributions. *Psychol Med.* 2015;**45**:2985-97. doi: 10.1017/S0033291715000926.

Goss AJ, Kaser M, Costafreda SG, Sahakian BJ, Fu CH. Modafinil augmentation therapy in unipolar and bipolar depression: a systematic review and meta-analysis of randomized controlled trials. *J Clin Psychiatry* 2013; **74**:1101-7. doi: 10.4088/JCP.13r08560.

Kaiser RH, Andrews-Hanna JR, Wager TD, Pizzagalli DA. Large-Scale network dysfunction in major depressive disorder: a meta-analysis of resting-state functional connectivity. *JAMA Psychiatry.* 2015;**72**:603-11. doi: 10.1001/jamapsychiatry.2015.0071.

Keller JB, Hedden T, Thompson TW, Anteraper SA, Gabrieli JD, Whitfield-Gabrieli S. Resting-state anticorrelations between medial and lateral prefrontal cortex: association with working memory, aging, and individual differences. *Cortex.* 2015;**64**:271-80. doi: 10.1016/j.cortex.2014.12.001

Kelly AM, Uddin LQ, Biswal BB et al. Competition between functional brain networks mediates behavioral variability. *Neuroimage.* 2008;**39**:527-37.

Lee JC, Blumberger DM, Fitzgerald PB, Daskalakis ZJ, Levinson AJ. The role of transcranial magnetic stimulation in treatment-resistant depression: a review. Curr Pharm Des. 2012;18:5846-52.

Lobban F, Dodd AL, Sawczuk AP et al. Assessing Feasibility and Acceptability of Web-Based Enhanced Relapse Prevention for Bipolar Disorder (ERPonline): A Randomized Controlled Trial. J Med Internet Res. 2017 Mar 24;19(3):e85. doi: 10.2196/jmir.7008.

McAllister-Williams RH, Anderson IM, Finkelmeyer A, et al. Antidepressant augmentation with metyrapone for treatment-resistant depression (the ADD study): A double-blind, randomised, placebo-controlled trial. *Lancet Psychiatry* 2016; **3:**117–27. doi:10.1016/S2215-0366(15)00436-8

McDermott LM, Ebmeier KP. A meta-analysis of depression severity and cognitive function. *J Affect Disord.* 2009;**119**:1-8. doi: 10.1016/j.jad.2009.04.022

Rock PL, Roiser JP, Riedel WJ, Blackwell AD. Cognitive impairment in depression: a systematic review and meta-analysis. *Psychol Med.* 2014;**44**:2029-40. doi: 10.1017/S0033291713002535.

Rush AJ, Trivedi MH, Ibrahim HM, et al. The 16-item Quick Inventory of Depressive Symptomatology (QIDS) Clinician Rating (QIDS-C) and Self-Report (QIDS-SR): A psychometric evaluation in patients with chronic major depression. *Biol Psychiatry* 2003; **54**: 573-583. doi: 10.1016/s0006-3223(02)01866.

Schmiedek F, Oberauer K, Wilhelm O, et al. Individual differences in components of reaction time distributions and their relations to working memory and intelligence. *J Exp Psychol General* 2007; **136**: 414-429.

Tundo A, de Filippis R, De Crescenzo F. Pramipexole in the treatment of unipolar and bipolar depression. A systematic review and meta-analysis. *Acta Psychiatr Scand* 2019; **140**:116-125. doi: 10.1111/acps.13055.
