# Supplementary material for: Connectivity guided theta burst transcranial magnetic stimulation versus repetitive transcranial magnetic stimulation for treatment-resistant moderate to severe depression: study protocol for a randomised double-blind controlled trial (BRIGhTMIND)
Source: BMJ Open. 2020 Jul 7;10(7):e038430. doi: 10.1136/bmjopen-2020-038430 (PMC7342821; doi:10.1136/bmjopen-2020-038430)
Supplement: Author's manuscript [file bmjopen-2020-038430.draft_revisions.pdf]

BMJ Open is committed to open peer review. As part of this commitment we make the peer review history of every article we publish publicly available.

When an article is published we post the peer reviewers' comments and the authors' responses online. We also post the versions of the paper that were used during peer review. These are the versions that the peer review comments apply to.

The versions of the paper that follow are the versions that were submitted during the peer review process. They are not the versions of record or the final published versions. They should not be cited or distributed as the published version of this manuscript.

BMJ Open is an open access journal and the full, final, typeset and author-corrected version of record of the manuscript is available on our site with no access controls, subscription charges or pay-per-view fees (http://bmjopen.bmj.com).

If you have any questions on BMJ Open's open peer review process please email info.bmjopen@bmj.com

BMJ Open

Connectivity guided theta burst transcranial magnetic stimulation versus repetitive transcranial magnetic stimulation for treatment resistant moderate to severe depression: study protocol for a randomised double-blind controlled trial (BRIGhTMIND).

Journal:	*BMJ Open*
Manuscript ID	bmjopen-2020-038430
Article Type:	Protocol
Date Submitted by the Author:	11-Mar-2020

Complete List of Authors:	Morriss, Richard; University of Nottingham, Psychiatry<br>Webster, Lucy; Nottinghamshire Healthcare NHS Foundation Trust<br>Abdelghani, Mohamed; Camden and Islington NHS Foundation Trust<br>Auer, Dorothee; University of Nottingham, Arthritis Research UK Pain Centre; University of Nottingham, Sir Peter Mansfield Imaging Centre<br>Barber, Shaun; University of Leicester<br>Bates, Peter<br>Blamire, Andrew; University of Newcastle upon Tyne<br>Briley, Paul; University of Nottingham<br>Brookes, Cassandra; University of Leicester, Leicester Clinical Trials Unit<br>Iwabuchi, Sarina; University of Nottingham<br>James, Marilyn; University of Nottingham, School of Medicine<br>Kaylor-Hughes, Catherine; University of Nottingham<br>Lankappa, Sudheer; Nottinghamshire Healthcare NHS Foundation Trust<br>Liddle, Peter; University of Nottingham<br>McAllister-Williams, Hamish; University of Newcastle upon Tyne<br>O'Neill-Kerr, Alex; Northamptonshire Healthcare NHS Foundation Trust<br>Pszczolkowski Parraguez, Stefan; University of Nottingham<br>Suazo Di Paola, Ana; University of Leicester, Leicester Clinical Trials Unit<br>Thomson, Louise; University of Nottingham, Psychiatry<br>Walters, Yvette; University of Leicester, Leicester Clinical Trials Unit

Keywords:	Depression & mood disorders < PSYCHIATRY, Magnetic resonance imaging < RADIOLOGY & IMAGING, Health economics < HEALTH SERVICES ADMINISTRATION & MANAGEMENT, Adult psychiatry < PSYCHIATRY, Clinical trials < THERAPEUTICS

SCHOLARONE™
Manuscripts

I, the Submitting Author has the right to grant and does grant on behalf of all authors of the Work (as defined in the below author licence), an exclusive licence and/or a non-exclusive licence for contributions from authors who are: i) UK Crown employees; ii) where BMJ has agreed a CC-BY licence shall apply, and/or iii) in accordance with the terms applicable for US Federal Government officers or employees acting as part of their official duties; on a worldwide, perpetual, irrevocable, royalty-free basis to BMJ Publishing Group Ltd ("BMJ") its licensees and where the relevant Journal is co-owned by BMJ to the co-owners of the Journal, to publish the Work in this journal and any other BMJ products and to exploit all rights, as set out in our licence.

The Submitting Author accepts and understands that any supply made under these terms is made by BMJ to the Submitting Author unless you are acting as an employee on behalf of your employer or a postgraduate student of an affiliated institution which is paying any applicable article publishing charge ("APC") for Open Access articles. Where the Submitting Author wishes to make the Work available on an Open Access basis (and intends to pay the relevant APC), the terms of reuse of such Open Access shall be governed by a Creative Commons licence – details of these licences and which Creative Commons licence will apply to this Work are set out in our licence referred to above.

Other than as permitted in any relevant BMJ Author's Self Archiving Policies, I confirm this Work has not been accepted for publication elsewhere, is not being considered for publication elsewhere and does not duplicate material already published. I confirm all authors consent to publication of this Work and authorise the granting of this licence.

For peer review only

Connectivity guided theta burst transcranial magnetic stimulation versus repetitive transcranial magnetic stimulation for treatment resistant moderate to severe depression: study protocol for a randomised double-blind controlled trial (BRIGhTMIND).

Richard Morriss, Professor of Psychiatry, University of Nottingham

Lucy Webster, Research Assistant, Nottinghamshire Healthcare NHS Foundation Trust

Mohamed Abdelghani, Consultant Psychiatrist, Camden and Islington NHS Foundation Trust,

Dorothee Auer, Professor of Neuroimaging, University of Nottingham,

Shaun Barber, medical statistician, University of Leicester

Peter Bates, PPI co-ordinator, University of Nottingham

Andrew Blamire, Professor of Neuroimaging, University of Newcastle

Paul Briley, Academic Clinical Fellow, University of Nottingham

Cassandra Brookes, Principal medical statistician, University of Leicester

Sarina Iwabuchi, Research Fellow, University of Nottingham

Marilyn James, Professor of Health Economics, University of Nottingham

Catherine Kaylor-Hughes, Research Fellow, University of Nottingham

Alex O'Neill-Kerr, Consultant Psychiatrist, University of Northampton

Sudheer Lankappa, Consultant Psychiatrist, Nottinghamshire Healthcare NHS Foundation Trust

Peter Liddle, Professor of Psychiatry, University of Nottingham

R. Hamish Mc-Allister-Williams, Professor of Affective Disorders, Newcastle University and Honorary Consultant Psychiatrist, Cumbria, Northumberland, Tyne and Wear NHS Foundation Trust

Stefan Pszczolkowski Parraguez, research Fellow, University of Nottingham

Ana Suazo Di Paola, Medical Statistician, University of Leicester

Louise Thomson, Senior Research Fellow, University of Nottingham

Yvette Walters, trial manager, University of Leicester

BRIGhTMIND study team- Lorraine Bastick, Rosie Carr, Alison Cartlidge, Harry Clark, William Cottam, Robert De Vai, Linda Davison, John Gledhill, Adele Gregory, Christopher Griffiths, Andrew Hamilton, Delilah Harding, Kelly Heath, Rachel Hobson, Gbeminiyi Ireoluwa, Najat Khalifa, Kate Johnstone, Charlotte Kirkland, Mark Liddle, Jessica Lynch, Neil Nixon, Jehill Parikh, Isabel Reid, Noemi Reiner, Sandra Simpson, Beverley Smith, Tina Sore, Joseph Stone, Carly Taylorson, Rebecca Toney, Claire Turner, Sarah Wilkinson, Andy Willis, Tom Willis

Corresponding author: Professor Richard Morriss, Institute of mental Health, University of Nottingham, Triumph Road, Nottingham, NG7 2TU. E-mail: richard.morriss@nottingham.ac.uk

For peer review only - http://bmjopen.bmj.com/site/about/guidelines.xhtml

Abstract

Introduction The BRIGhTMIND study aims to determine the clinical effectiveness, cost effectiveness and mechanism of action of connectivity guided intermittent Theta Burst Stimulation (cgiTBS) versus standard repetitive Transcranial Magnetic Stimulation (rTMS) in adults with moderate to severe treatment resistant depression.

Methods and analysis The study is a randomised double-blind controlled trial with 1:1 allocation to either 20 sessions of (a) cgiTBS or (b) standard rTMS not using connectivity guidance. A total of 368 eligible participants with a diagnosis of current unipolar major depressive disorder that is both treatment resistant (defined as scoring 2 or more on the Massachusetts General Hospital (MGH) Staging Score) and moderate to severe (scoring ≥16 on the 17-item Hamilton Depression Rating Scale (HDRS-17)), will be recruited from primary and secondary care settings at four treatment centres in the United Kingdom. The primary outcome is depression response at 16 weeks (50% or greater reduction in HDRS-17 score from baseline). Secondary outcomes include assessments of self-rated depression, anxiety, psychosocial functioning, cognition and quality of life at 8, 16 and 26 weeks post randomisation. Cost effectiveness, patient acceptability, safety, mechanism of action and predictors of response will also be examined.

Ethics and dissemination Ethical approval was granted by East Midlands Leicester Central Research Ethics Committee (ref: 18/EM/0232) on 30th August 2018. The results of the study will be published in relevant peer-reviewed journals, and then through professional and public conferences and media. Further publications will explore patient experience, moderators and mediators of outcome and mechanism of action.

Trial registration number ISRCTN19674644; Pre-results.

252 words

Strengths and Limitations of the study

- Large multicentre randomised controlled trial of cgiTBS versus standard rTMS.
- Carefully characterised population of patients with treatment resistant moderate to severe unipolar major depression with broad inclusion and exclusion criteria to enhance generalisability to clinical care.

For peer review only - http://bmjopen.bmj.com/site/about/guidelines.xhtml

- Explores efficacy, sustainability of treatment response, acceptability, cost effectiveness cognitive function, mechanism of action using MRI and spectroscopy, and clinical and MRI predictors of response in the same sample.
- Unable to completely blind sound and sensation of the treatments being tested.
- Both nature of magnetic stimulation (TBS vs rTMS) and stimulation site identification vary between treatment arms.

Introduction

The lifetime prevalence of major depressive disorder (MDD) is approximately 13% of the general population,[1] and it is the most disabling health condition in terms of years lived with disability.[2] Antidepressants and psychotherapies are effective in treating MDD. However, at least 33% of patients in specialist care,[3] and 22% in general primary care,[4] fail to respond to adequate trials of at least two antidepressants. Therefore, it is essential to find alternative treatments for such “treatment-resistant depression” (TRD).

Neuromodulation techniques that attempt to directly modulate the function of targeted brain regions, such as electroconvulsive therapy (ECT),[5] and repetitive transcranial magnetic stimulation (rTMS),[6] can be effective treatments for MDD. Unlike ECT, rTMS does not require anaesthesia nor produce potential cognitive deficits, and is cost effective in TRD compared with current standard care.[7]

A recent meta-analysis in TRD demonstrated the acute effectiveness of rTMS when compared with sham TMS, showing significant reductions in depression symptoms, and increases in response and remission and response rates, after treatment [6]. However, the beneficial effects of rTMS on mood in TRD may be relatively short, lasting only 1-3 months. [6,8]

Theta burst stimulation (TBS) is an alternative patterned form of TMS, which employs high frequency stimulation, with each treatment administration requiring less time. Unlike rTMS, TBS is associated with cortical long-term potentiation that may induce plasticity in more distal brain areas, such as the hippocampus, potentially leading to a higher proportion of responders and a longer duration of effect in TRD.[9] A meta-analysis of five randomised controlled trials (RCTs) in 221 patients with MDD,[10] found unilateral intermittent TBS (iTBS) and bilateral iTBS to be robustly effective versus sham TBS on depression symptoms and response. One RCT demonstrating 83% of TRD patients given unilateral iTBS maintained their response at 14 weeks unlike sham and bilateral TBS [11]. A large RCT of 414 TRD patients comparing iTBS versus rTMS to the left dorsolateral prefrontal cortex (DLPFC) showed a non-inferior reduction in depression symptoms after 4-6 weeks of treatment and at 1, 4 and 12 weeks after treatment.[12] Thus, TBS requires a shorter duration of administration than rTMS without

For peer review only - http://bmjopen.bmj.com/site/about/guidelines.xhtml

compromising clinical effectiveness. Further studies are required to maximise efficacy and durability of effect of TBS, identify its mechanisms of action and predictors of response.

Brain connectivity changes as detected by task-free, resting state functional magnetic resonance imaging (rsfMRI) may individualise neurostimulation therapy of MDD,[13] with the insula suggested as a target for neuromodulation.[14] In depression, an altered network communication exists within and between affective, cognitive control and default mode networks,[15-18] with a disruption of the reciprocal loop between the DLPFC and insula extending to the sensory regions.[16] In addition, connectivity of the insula predicts efficacy of cognitive behaviour therapy and antidepressant treatment in patients with depression [19]. One biotype, characterised by strong connectivity between the insula and other regions of the brain, was related to partial treatment response to rTMS of at least 25% improvement on depression symptoms in 82.5% participants.[20]

Therefore, in a small pilot study of 27 TRD patients,[21] individualised left DLPFC targets were determined using Granger causality analysis (GCA) to provide a measure of effective functional connectivity (eFC) seeded from the right anterior insula (rAI). Of the 18 participants who completed all follow-up in the two treatment arms (connectivity guided rTMS (cgrTMS) and connectivity guided iTBS (cgiTBS)) higher response rates were found at three-month follow-up favouring cgiTBS (89%) over cgrTMS (44%), indicating the potential for longer lasting efficacy of cgiTBS.

In addition, rsfMRI and magnetic resonance spectroscopy (MRS) may advance neuromodulation therapy through mechanistic evaluation of effects and response predictions. iTBS may dampen fronto-insular eFC in responders with TRD [21]. Left DLPFC GABA levels are increased significantly in rTMS responders versus non-responders.[22] In addition, DLPFC targeted TMS normalises dysfunctional fronto-limbic networks in TRD, with decreased hyperconnectivity between the subgenual anterior cingulate cortex, ventromedial and dorsomedial prefrontal cortex and DLPFC.[23]

iTBS may induce widespread and longer-term network change but precise anatomical localisation of the target circuitry may be required for maximal efficacy of iTBS to normalise dysfunctional fronto-limbic circuitry. Such changes may increase the proportion of people with TRD who obtain a sustained response compared to standard rTMS. Thus, this multi-centre RCT will examine the clinical and cost effectiveness of cgiTBS in comparison with standard rTMS, in treatment resistant moderate to severe MDD.

For peer review only - http://bmjopen.bmj.com/site/about/guidelines.xhtml

Methods and analysis

Study Design

The study is a multicentre parallel group, double blind, randomised, controlled trial of the efficacy of cgiTBS versus standard rTMS without connectivity guidance, in patients with a primary diagnosis of moderate to severe MDD, which is treatment resistant in their current episode (TRD). The study will be carried out at four sites across UK National Health Services (NHS): Nottinghamshire Healthcare Foundation NHS Trust, Northamptonshire Healthcare NHS Foundation Trust, Cumbria, Northumberland, Tyne and Wear NHS Foundation Trust and Camden and Islington NHS Foundation Trust. The study flow chart (Figure 1) provides an overview of the study design and procedures.

[Insert Figure 1]

Study population and recruitment

368 participants will be recruited in total (184 per treatment arm). Table 1 shows the detailed inclusion/exclusion criteria. Participant identification will occur through primary and secondary care NHS settings across the four sites.

Table 1. Inclusion and Exclusion Criteria

Inclusion Criteria	Exclusion Criteria
• Adults $\geq$ 18 years<br>• Diagnosis of current MDD (defined according to the Diagnostic and Statistical Manual of Mental Disorders, Fifth Edition), that is treatment resistant defined as scoring 2 or more on the Massachusetts General Hospital Treatment Resistant Depression staging score (appendix 1).<br>• Have a 17-Item Hamilton Depression Rating Scale score of 16 or more (moderate to severe depression).<br>• Capacity to provide informed consent before any trial related activities.	• History of bipolar disorder (due to risk of mania) or depression secondary to other mental disorder.<br>• Neurological conditions e.g. brain neoplasm, cerebrovascular events, epilepsy, neurodegenerative disorders, and prior brain surgery.<br>• Standard contraindications to MRI i.e. irremovable metal objects in and around body e.g. cardiac pacemaker, implanted medication pump, and pregnancy (any doubt resolved by pregnancy test, women of childbearing age taking precautions against

For peer review only - http://bmjopen.bmj.com/site/about/guidelines.xhtml

	pregnancy) This will include other potential complicated factors such as red tattoo's which consist of iron on the head, neck and back and claustrophobia (we offer mock scanner testing and training in some sites).<br>• Major unstable medical illness requiring further investigation or treatment.<br>• Change in prescribed medication 2 weeks before baseline assessment.<br>• Prescription of lamotrigine, gabapentin, pregabalin in the 2 weeks prior to baseline assessment.<br>• Daily prescription of benzodiazepine above 5 mg diazepam equivalents, Zopiclone above 7.5mg, zolpidem above 10mg or Zaleplon above 10mg. These drugs should not be used intermittently in the 2 weeks before baseline assessment.<br>• Current substance abuse or dependence (defined by DSM-5 criteria).<br>• Prior TMS treatment.<br>• At risk of suicidality.<br>• Potential complicated factors relating to the TMS treatment i.e. Hairstyles which would impair magnetic transmission and piercings. (Participants would only be excluded if they chose to not make the changes required to ensure effective treatment.)<br>• Involved with any other clinical trial at the time of consent or 6 months prior.<br>• Unable to read or understand English.

Interventions

For all treatments, a 70mm figure-of-eight coil (E-z Cool coil) and a Magstim® Horizon® Performance Stimulator with StimGuide® Navigated TMS Package (Magstim Company, Whitland, United Kingdom)

For peer review only - http://bmjopen.bmj.com/site/about/guidelines.xhtml

will be used. Participants in both treatment arms will receive neuronavigation guided single coil stimulation for 20 TMS sessions delivered over 4-6 weeks. A total of 3000 pulses will be delivered in each rTMS or cgiTBS session. Each treatment session, irrespective of treatment arm, is designed to last approximately 37.5 minutes in total for the purposes of blinding the participants and assessors of outcome. There must not be a gap of more than 4 days between treatments in the course of 20. Medication will be unchanged over 16 weeks unless there is clinical risk of harm.

Individuals assigned to cgiTBS will receive bursts of 3 pulses (80% motor threshold) at 50Hz applied at a frequency of 5 Hz (i.e. every 200 ms) for 40 seconds duration over a site determined from the assessment of maximal strength of effective connectivity between the right anterior insula and the left DLPFC from functional magnetic resonance imaging (fMRI) and structural magnetic resonance imaging (MRI). Neuronavigation computes the nearest location for TBS stimulus on the scalp from three fiducal points, the nasion, left preauricular and right preauricular sites. If this site causes discomfort to the participant, it is moved by 1 cm in any direction until there is no discomfort. The pulses are repeated for a total of 5 runs with 5-minute rest intervals between runs.

Individuals assigned to rTMS will follow the standard US Food and Drug Administration (FDA) approved protocol. The site of stimulation will be determined using structural and fMRI utilising neuronavigation to compute the F3 electrode site over the left DLPFC for TMS stimulation from the same three fiducal points. Stimulation is at 120% motor threshold with 75 x 4-second trains of 10Hz interspersed by 26-second intertrain intervals.

Primary Clinical Objective

Our primary hypothesis is that cgiTBS is more efficacious at 16 weeks than standard rTMS in patients with TRD as assessed by the proportion of patients who show a response (50% reduction in depression symptoms from baseline on the HDRS-17).

Secondary Clinical Objectives

- To explore secondary efficacy outcomes of the mean change in HDRS-17 scores between the two treatment arms and the proportion of patients meeting criteria for remission (HDRS-17 score ≤7) at 16 weeks.
- To explore secondary clinical outcomes of importance to patients and clinicians namely cognition, anxiety, social function and quality of life.
- To examine cost effectiveness of cgiTBS versus rTMS in a UK NHS population.
- To examine the patient acceptability and patient experience of cgiTBS and rTMS.

For peer review only - http://bmjopen.bmj.com/site/about/guidelines.xhtml

Mechanistic Objectives

- To investigate the neural mechanism of efficacy in cgiTBS and rTMS.
- To develop response prediction models from brain biotypes and clinical features.

Protocols for the study will be published separately describing the mechanistic objectives and localisation of sites of stimulation in greater detail.

Study Schedule

Trained researchers under the supervision of clinically trained investigators will obtain informed consent and undertake all assessments. A pre-screening questionnaire will be used to telephone screen interested participants first, with potentially eligible participants invited to attend a baseline assessment. Written informed consent will be obtained and study eligibility determined at the baseline assessment.

Information on socio-demographics, past medical and psychiatric history including a detailed assessment of treatment resistance will be obtained from case files and primary care notes. Diagnosis will be assessed by the Structured Clinical Interview (SCID-5)[24] for the Diagnostic and Statistical Manual of Mental Disorders, Fifth Edition (DSM-5).[25]

Treatment resistance will be measured by the Massachusetts General Hospital (MGH) TRD staging score (adapted for new treatment options - see supplementary material, appendix 1).[26]

Depression severity and the primary outcome measure will be assessed using the GRID version of HDRS-17.[27] The HDRS-17 and secondary outcome measures will be assessed in eligible patients at baseline, 8, 16 and 26 weeks post randomisation (Table 2). The primary outcome measure is a binary variable of responder or non-responder at 16 weeks. Individuals observed to have a 50% drop or greater in HDRS-17 from baseline to 16 weeks are defined as responders. In contrast, individuals with <50% reduction or a null value are classified as non- responders. The Childhood Trauma Questionnaire (CTQ)[28] will be assessed at baseline as a moderator of treatment response at 16 weeks.

Secondary outcome measures are the Beck Depression Inventory-II (BDI-II),[29] Patient Health Questionnaire (PHQ-9),[30] Generalised Anxiety Disorder Assessment (GAD-7),[31] Work and Social Adjustment Scale (WSAS),[32] EuroQol-5D-5L,[33] and cognitive functioning as measured by the THINC-Integrated Tool (THINC-it) (THINC-it Task Force, http://thinc.progress.im/en). QIDS-SR [34] is completed for a mechanism study (see supplementary material – Appendix 2).

For peer review only - http://bmjopen.bmj.com/site/about/guidelines.xhtml

In addition, a purposely designed patient proforma will be used to collect patient NHS service utilisation information at the 16 and 26 week follow up time points. This will cover relevant items listed in the Client Service Receipt Inventory (CSRI),[35] and tailor the resources items measured following good practice approaches used by the health economics DiRUM (database of instruments for resource use measurement) group to estimate costs. Patient acceptability will be measured on a 1-5 scale from unacceptable to acceptable and patient experience of overall improvement will be assessed using the Patient Global Impression of Change (1-5 scale much worse to much better)[36] after each TMS session and at each follow up time point. A side effect checklist will be completed after each TMS session and 8 weeks follow up assessment. We will note any changes in medication or other forms of treatment.

Table 2. Study Schedule and Assessments

**Outcome measures**	**Baseline Assessment (Consent to the study)**	**Baseline MRI Scan**	**Treatment Mon- Fri for 4-6 weeks**	**8 Week Follow up Assessment**	**16 Week Follow Up Assessment**	**16 Week MRI Scan**	**26 Week Follow Up Assessment**
Visit Window		Within 14 days of Baseline Assessment	+ 14 days of MRI Scan	+/- 1 week from Randomisation	+/- 1 week from Randomisation	Within 14 days of 16 Week Follow Up Assessment	+/- 1 week from Randomisation
HDRS-17	✓		✓Only if Baseline assessment exceeds 4 weeks	✓	✓		✓
MGH	✓		✓ Only if Baseline assessment exceeds 4 weeks				
BDI-II	✓			✓	✓		✓
PHQ-9	✓			✓	✓		✓
WSAS	✓			✓	✓		✓
GAD7	✓			✓	✓		✓
EQ-5D-5L	✓			✓	✓		✓
THINC-it	✓			✓	✓		✓
SCID-5	✓						
CTQ	✓						
QIDS-SR	✓			✓	✓		✓
Client Resource Questionnaire	✓				✓		✓
Patient Acceptability			✓	✓	✓		✓
Side Effects Checklist (Adverse Events)			✓	✓			
fMRI		✓				✓	
MRI		✓				✓	
rsfMRI)		✓				✓	
MRS		✓				✓	
Diffusion weighted imaging		✓				✓	

For peer review only - http://bmjopen.bmj.com/site/about/guidelines.xhtml

Participants will undergo baseline structural MRI, resting state fMRI, diffusion weighted imaging scans to assess structural connections between relevant brain regions, and MRS scans (not in London where only fMRI and structural MRI scans will be performed). Treatment target identification will be analysed centrally in Nottingham and communicated to treatment sites. Using GCA of fMRI scans in each subject, coordinates for the stimulation target within the left DLPFC that shows maximal effective connectivity with the right anterior insula will be identified. For the MRS scans, voxels will be placed in the DLPFC and anterior cingulate cortex (ACC), using a similar method to prior work with the MEGA-PRESS sequence for GABA-edited MRS. [22] The MRI scans are repeated at 16 weeks to assess functional connectivity, effective connectivity and GABA.

Randomisation

Randomisation will take place immediately prior to the start of the first treatment session, with participants randomised to either rTMS or cgiTBS treatments. Randomisation will be conducted via a web-based randomisation system (Sealed Envelope, www.sealedevelope.com) by a named TMS nurse practitioner and health care assistant delivering TMS at each centre who will remain un-blinded. Participants will be randomly assigned in a 1:1 ratio into the rTMS and cgiTBS arms using blocks of varying size. Randomisation will be stratified by centre and minimised on severity of depression at baseline and degree of treatment resistance. Baseline depression will be measured by HDRS-17 score and classified as moderate (16-23) or severe (≥24). [37]. Treatment resistance will be measured by the MGH TRD staging score and, based on distributions of treatment resistance in the ADD study ,[38], degree of treatment resistance will be classified as low (2-3.5), medium (4-6) and high (≥6.5).

Statistical Analyses

Efficacy analysis

The primary analysis will test the null hypothesis that treatment with cgiTBS does not increase response rate as measured by HDRS-17 compared to rTMS at 16 weeks on an intent to treat population with any missing response data at 16 weeks being imputed as no response having been achieved. A logistic regression will be fitted for the outcome of response with treatment arm, centre, baseline HDRS-17 score and MGH TRD score.

For peer review only - http://bmjopen.bmj.com/site/about/guidelines.xhtml

As a secondary analysis a logistic regression model will be repeated in completers (those with 10 or more rTMS or cgiTBS treatments, assessed at baseline and 16 weeks) as a per protocol analysis. Secondary outcomes of the mean improvement in HDRS-17 scores and proportion of remitters at 8, 16 and 26 weeks, and sustained responders at both 16 and 26 weeks, will be compared between groups using logistic regression adjusted for treatment centre, baseline HDRS-17 score and MGH TRD score (5% significance). All outcomes are shown in Table 3. Repeated measure linear models will be used for continuous outcomes. Patient acceptability (5-point scale) and safety of both cgiTBS and rTMS (side-effects checklist) will be reported descriptively.

We will explore moderators of depression response at 16 weeks, namely severity of depression by baseline HDRS-17 score, degree of treatment resistance, childhood trauma and age. The number of TMS sessions attended will be explored as a mediator of outcome in exploratory sub-group analyses of the depression response at 16 weeks.

Table 3. Study efficacy outcome measures

Outcomes	Measure	Time Points
Primary Outcome	17-Item Hamilton Depression Rating Scale (HDRS-17)	Response at 16 weeks (defined as by a 50% or greater reduction in HDRS-17 score from baseline)
Secondary Outcomes	HDRS-17	Mean change in HDRS-17 score compared with baseline at 8, 16 and 26 weeks
		Response (as defined above) at 8 weeks and at 26 weeks
		Sustained response at 16 and 26 weeks (defined as a continuing response as defined above following a response at the previous time point)
		Remitters at 8, 16 and 26 weeks (defined as a score of 7 or less on the HDRS-17)
	Beck Depression Inventory-II (BDI-II)	At 8, 16 and 26 weeks
	Patient Health Questionnaire (PHQ-9)	At 8, 16 and 26 weeks
	Generalised Anxiety Disorder Assessment (GAD-7)	At 8, 16 and 26 weeks
	THINC Integrated Tool (THINC-it)	At 8, 16 and 26 weeks
	EuroQol-5D-5L	At 8, 16 and 26 weeks
	Work and Social Adjustment Scale (WSAS)	At 8, 16 and 26 weeks
	Patient Acceptability (1-5 scale)	After each TMS session and at 8, 16 and 26 weeks

For peer review only - http://bmjopen.bmj.com/site/about/guidelines.xhtml

Adverse Events Checklist	After each TMS session and 8 weeks
MRI scan (structural [T1], diffusion weighted imaging [DWI] for structural connectivity, resting state fMRI for functional connectivity [FC] and effective connectivity [eFC], and GABA MRS of the DLPFC	T1, DWI, FC, eFC at baseline (all centres)<br>GABA at baseline and T1, DWI, FC, eFC, GABA at 16 weeks (all sites except London).

Economic analysis

The economics analysis will take an NHS and personal social services cost perspective in accordance with NICE guidance, including medication, inpatient and outpatient hospital visits and primary and community care use. It will also take a wider societal perspective to capture the broader effects of rTMS and cgiTBS on depression such as paid employment, caring responsibilities and other implications for friends and family. Resource data will then form the units on which cost data, using sources such as the Unit Cost of Health and Social Care\PSSRU,[39] the British National Formulary (BNF), and national reference costs can be attached. The nurse and health care assistant at each centre will complete a diary of time spent managing each participant in the RCT to derive treatment costs for rTMS and cgiTBS. The number of treatment sessions for the alternative therapies will be carefully recorded and a separate intervention cost assigned to each of the therapies. We will delineate the time spent delivering cgiTBS or rTMS from time spent on research only procedures, such as imaging for delivery of rTMS and additional mechanistic scans in both treatment arms or the extended length of the TBS session for blinding, that would not be used in the real clinical world. The outcome measure for the economic evaluation will be the number of quality-adjusted life year (QALYs) based on a six-month time horizon with no discounting for costs or outcomes as they accrue within a 12-month period. An incremental analysis will be used between the two groups and where appropriate an incremental cost effectiveness ratio (ICER) will be reported between rTMS and cgiTBS. We will use the net monetary benefit framework and implement a net benefit regression,[40] to estimate the extent to which, and the probability that, the cgiTBS intervention is cost-effective compared to standard rTMS at a range of threshold values for the willingness to pay per QALY, generating cost effectiveness acceptability curves (CEACs). Data will be analysed for baseline and centre effects. Key cost drivers will be examined using probabilistic sensitivity analysis.

For peer review only - http://bmjopen.bmj.com/site/about/guidelines.xhtml

Qualitative analysis

A purposive sample of 25-30 participants from both arms and all centres, reflecting a mix of demographic characteristics, consent or non-consent to participate, adherence and non-adherence to treatment and follow up will be selected for qualitative interviews, each lasting for up to an hour, after the 16-week assessment (the primary outcome). We will ask about their general views of TMS, benefits from, disadvantages from or dislikes about receiving TMS. Interviews will continue until theme saturation is achieved, i.e. no more themes emerge in subsequent interviews. Qualitative interviews of barriers to recruitment, with staff members involved in recruitment, will also be completed to optimise strategies to improve recruitment as the RCT proceeds. All interviews will be recorded and transcribed verbatim. Inductive thematic analysis using a grounded approach will be adopted.

Sample Size

Sample size is calculated on the minimum clinically important difference in responder rates from baseline to 16 weeks of 15% (equivalent to a number needed to treat (NNT) of 7) between two active treatments (cgiTBS versus rTMS) in favour of the experimental condition cgiTBS. This is an effect size regarded in the literature as a clinically important difference in studies using invasive approaches to manage TRD such as vagal nerve stimulation.[40] The most recent meta-analysis of 16 RCTs in 977 participants with TRD reports a response rate on the HDRS-17 and the Montgomery–Åsberg Depression Rating Scale of 26.5% for rTMS at the end of treatment versus 13.1% for sham TMS.[6] Assuming a response rate on the HDRS-17 of 26.5% with rTMS and 41.5% with cgiTBS, 368 patients in total (184 per arm) will provide a power of 80% to detect the difference in response rates at a 5% significance level (two-tailed), allowing for 15% loss to follow-up at 16 weeks [6].

Blinding

Patients, referring clinical teams and the outcomes assessors will be kept blind with respect to the treatment protocol assigned and administered. Any unintended unblinding of outcome assessors will be recorded and another assessor will complete all further assessments for that participant. At each assessment, the outcomes assessor will be asked to guess the treatment allocation of the participant.

For peer review only - http://bmjopen.bmj.com/site/about/guidelines.xhtml

Ethics and dissemination

Ethical Considerations

Ethical approval was granted by East Midlands Leicester Central Research Ethics Committee (ref: 18/EM/0232) on 30/08/2018. The study was registered on 02/10/2018 (ISRCTN19674644) under the public title "BRIGhTMIND: brain imaging guided transcranial magnetic stimulation in depression". The first participant was randomised on 22/01/19. Study finish is scheduled for September 2021. Overview of the study is performed by an external independent Data Monitoring Ethics Committee reporting to a Trial Steering Committee.

Expenses will be covered for participation in the study along with a £10.00 shopping voucher at 16 and 26 Week follow up assessments as a mark of respect and gratitude for the time and input of the participants to the follow up aspects of the trial.

Safety Considerations

Internationally agreed definitions of adverse events (any untoward medical occurrence in a clinical trial subject administered TMS whether or not it has a causal relationship with TMS) and serious adverse events (any adverse event or adverse reaction that results in death, is life-threatening, requires hospitalisation or prolongation of existing hospitalisation, results in persistent or significant disability or incapacity, or is a congenital anomaly or birth defect). All participants will be asked about adverse events after every treatment (immediately and a maximum of 72 hours later) until they are resolved. Any participant found to be at risk to themselves (suicide, neglect) or others, or developing a serious adverse event will be referred to relevant clinical services. A review by a clinical expert in TRD will be offered to any participant whose depression has become more severe at 16 and 26 weeks for safety reasons.

Dissemination

All participants will be sent a report summary of the results. Results of the study will be published in peer-reviewed academic journals and communicated widely to academic, profession and public audiences through conferences, media and social media.

For peer review only - http://bmjopen.bmj.com/site/about/guidelines.xhtml

Patient and public involvement.

Regarding this study a co-production approach has been taken. We worked with the Involvement Centre at Nottinghamshire Healthcare Trust to create a Magnetic Stimulation Advisory Group who co-produced the treatment pathway for prior pilot work [21] and the current study.

During the study, each centre will be invited to contribute at least two Patient and Public Involvement (PPI) representatives to form a Lived Experience Advisory Panel (LEAP) who will contribute to all study meetings and a representative will be invited to attend all research meetings. At each site PPI representatives will seek opinions, test ideas and gain support for the study. Participant information sheets and other study materials will be co-designed. They will see oversee the interpretation of our findings, particularly the emerging qualitative analysis on barriers to recruitment and patient acceptability of TMS. The PPI process will be overseen by an experienced PPI lead who has trained supported and mentored many PPI groups over recent years. The voice of experts by experience will be heard in all dissemination activities, including presentations or publications.

Discussion

Although TMS is an established efficacious and safe treatment approach for TRD, research is required to establish if it is possible to increase the proportion of people with TRD who derived a sustained improvement in depression response [8]. Based on theoretical and preliminary empirical evidence, this study explores the potential of using precise personalised neuroanatomical stimulation at a site where affective, cognitive control and default mode networks overlap in neuroanatomical space as determined by MRI derived maximal strength of connectivity between the right anterior insula and the left DLPFC. It will be compared with standard rTMS in a large multicentre randomised controlled trial. Both rTMS and iTBS are effective against sham treatments [6, 8, 10] and recommended for clinical practice in the UK [8]. Furthermore, standard iTBS is non-inferior but not superior to rTMS [12]. Thus, if the current RCT showed that cgiTBS was superior in efficacy, it would be reasonable to conclude that the increased efficacy was due to the personalisation of the site of iTBS treatment.

The design of the trial aims to have high external validity and generalisability to inform routine clinical care. Therefore, inclusion and exclusion criteria are broad and confined to ensuring that safety, efficacy and the investigation of mechanism of action are not compromised. Our definition of TRD [26] is one of the most widely used but it does not consider psychological treatment and underestimates treatment resistance in those who refuse or are not offered antidepressants or ECT in the current episode because of lack of effectiveness or intolerance in previous depression episodes. The broad

For peer review only - http://bmjopen.bmj.com/site/about/guidelines.xhtml

inclusion and exclusion criteria, large sample size, inclusion of clinical and MRI variables and long duration of follow up will permit a wide variation in potential predictors and non-predictors of response for clinical practice and further research.

Compromises have been made to the design of the study to improve blinding. cgiTBS and rTMS have been matched procedurally so both use the same MRI and neuronavigation system, although these are not currently being used in clinical practice in UK. TMS treatments have also been matched in terms of pulses received and duration of treatment, although the duration of cgiTBS would be much shorter in clinical practice. The use of sham coils to mask the sound and sensation of iTBS and rTMS was explored but effective shams were not available. As a result, entry into the study is restricted to people who had never previously received TMS.

A co-productive approach has been taken with our patient and public LEAP group and this has been particularly important around informing participants accurately about the study and managing participant expectations. By doing this we hope to optimise recruitment and retention to the study.

In many countries, 3 Tesla MRI is increasingly available so that cgiTBS could be implemented into routine care for TRD. Should the RCT demonstrate that cgiTBS is clinically effective at 16 weeks and cost effective compared to rTMS, then the possibility of maintaining participants with one full course of cgiTBS with booster sessions two or three times per year using the same site of stimulation from a baseline MRI might be feasible. If it is not effective, then other parameters of delivering TMS should be the focus of research and development.

3998 words

Abbreviations

ACC: anterior cingulate cortex; BDI: Beck Depression Inventory; BNF: British National Formulary; CEACs; Cost effectiveness acceptability curves; cgiTBS: Connectivity Guided Intermitted Theta Burst Stimulation; cgrTMS: Connectivity Guided repetitive Transcranial Magnetic Stimulation; CRF: Case Report Forms; CSRI: Client Service Receipt Inventory; cTBS: continuous Theta Burst Stimulation; CTQ: Childhood Trauma Questionnaire; DiRUM: Data of instrument for resource use measurement; DLPFC: Dorsolateral Prefrontal Cortex; DMPFC: Dorsomedial Prefrontal Cortex; DSM-5: The Diagnostic and Statistical Manual of Mental Disorders, Fifth Edition; ECT: Electroconvulsive Therapy; eFC: Effective Functional Connectivity; FC: Functional Connectivity; FDA: Food and Drug Administration; fMRI: Functional Magnetic Resonance Imaging; GABA: Gamma-amino butyric acid; GAD-7: Generalised Anxiety Disorder Assessment; GCA: Granger Causality Analysis; glx: glutamate and glutamine; GRID-HAMD: 17-Item Hamilton Depression Rating Scale GRID form; HDRS-17: 17-Item Hamilton Depression Rating Scale; ICER; Incremental cost effectiveness ratio; iTBS: intermittent Theta Burst Stimulation;

For peer review only - http://bmjopen.bmj.com/site/about/guidelines.xhtml

MADRS: The Montgomery–Åsberg Depression Rating Scale; MDD: Major Depressive Disorder; MGH: Massachusetts General Hospital; MRI: Magnetic Resonance Imaging; MRS: Magnetic resonance spectroscopy; NHS: National Health Services; NICE: National Institute of Health and Care Excellence; NNT: Number need to treat; PHQ-9: Patient Health Questionnaire; QALY's: Quality-adjusted life year; rAI: right anterior insula; RCT: Randomised Controlled Trials; rsfMRI: resting state Functional magnetic resonance imaging; rTMS: repetitive Transcranial Magnetic Stimulation; SCID-5: Structured Clinical Interview for DSM-5; TBS: Theta Burst Stimulation; THINC-it: THINC Integrated Tool; TMS: Transcranial Magnetic Stimulation; TRD: Treatment Resistant Depression; WSAS: Work and Social Adjustment Scale

Acknowledgements

We thank the ongoing support of the Funders, the Patient and Public Involvement representatives, participants, research and treatment staff at the four centres, the NIHR Clinical Research Network in East Midlands, North East and North Thames, the clinical, methodological and lay members of the independent Data Monitoring Ethics Committee and Trial Steering Committees, and the Leicester Clinical Trial Unit and all other staff whom are involved in the study. RM is supported by the Nottingham National Institute for Health Research (NIHR) Biomedical Research Centre, NIHR MindTech Med Tech and in Vitro Centre and NIHR Applied Research Centre East Midlands.

Authors' contributions

All authors approved the final manuscript.

Richard Morriss is the chief investigator and guarantor of the project. He designed the study, obtained funding and wrote the final draft of the protocol.

Lucy Webster wrote operational guidelines for the project, qualitative analysis protocols and the first draft on this manuscript.

Mohammed Abdelghani is the principal investigator for the London site, obtained funding, and designed the TMS schedules.

Dorothee Auer is the neuroimaging lead, led some of the underpinning work designed the neuroimaging and obtained grant funding.

Shaun Barber carried out power calculations for the study and designed the statistical analysis

Peter Bates has led the patient and public involvement aspects of the project and their contributions to the operation of the project.

Andrew Blamire designed the neuroimaging and obtained grant funding.

Paul Briley designed some of the neuroimaging protocol and is analysing the imaging data

Cassandra Brookes designed the statistical analysis and obtained funding for the study.

Sarina Iwabuchi led some of the underpinning work, designed the neuroimaging and TMS parts of the protocol and obtained funding.

For peer review only - http://bmjopen.bmj.com/site/about/guidelines.xhtml

Marilyn James designed the health economic analysis, the health economic data collection forms and will be responsible for the health economic management of the trial and oversee all the health economic analysis**.**

Catherine Kaylor-Hughes wrote protocols for TMS in the study and carried out some of the neuroimaging analysis

Alex Kerr is the principal investigator for the Northampton site, obtained funding, and designed the TMS schedules.

Sudheer Lankappa, the principal investigator for the Nottingham site, carried out some of the underpinning work, obtained funding, and designed the TMS schedules.

Peter Liddle carried out some of the underpinning work and designed some of the neuroimaging and TMS work, and obtained funding.

Hamish Mc-Allister-Williams is the principal investigator for the Newcastle site, designed the cognitive assessment and assessment of treatment resistance and obtained funding

Stefan Pszczolkowski Parraguez designed and carried out the computer programming for TMS site location, helped design the Neuronavigation and TMS protocols, and is carrying out neuroimaging analysis.

Ana Suazo Di Paolo has helped designed the statistics protocols and is carrying out the statistical analysis

Louise Thomson is the qualitative research lead, designed the qualitative parts of the project and obtained funding.

Yvette Walters is the trial manager of the study and has led the writing of study operating procedures, ethics and governance protocols for the study.

Protocol version

Version 4.0 22/01/2020

Funding

The trial was supported by the Efficacy and Mechanism Evaluation (EME) Programme, an MRC and NIHR partnership. (Grant number 16/44/22). Equipment for the study was provided by Magstim Company Ltd

Sponsor

Research and Evidence Team, Nottinghamshire Healthcare NHS Foundation Trust. Contact e-mail: research@nottshc.nhs.uk. Neither the study sponsor nor funder has or will have any role in designing, collecting, analysing or interpreting the data or any study reports.

For peer review only - http://bmjopen.bmj.com/site/about/guidelines.xhtml

Competing Interests

Alex Kerr has received fees for consultancy with Magstim. No other competing interests declared.

References

1 Kessler RC, Bromet EJ. The epidemiology of depression across cultures. *Annu Rev Public Health.* 2013;34:119-38. doi: 10.1146/annurev-publhealth-031912-114409.

2 World Health Organisation. *Depression and Other Common Mental Disorders: Global Health Estimates.* Geneva: World Health Organization; 2017. Licence: CC BY-NC-SA 3.0

3 Rush AJ, Trivedi MH, Wisniewski SR, *et al.* Acute and longer-term outcomes in depressed outpatients requiring one or several treatment steps: A STAR*D report. *Am J Psychiatry* 2006;**163**:1905–17. doi:10.1176/ajp.2006.163.11.1905

4 Rizvi SJ, Grima E, Tan M, *et al.* Treatment-resistant depression in primary care across Canada. *Can J Psychiatry* 2014;**59**:349–57. doi:10.1177/070674371405900702

5 Khalid N, Atkins M, Tredget J, *et al.* The effectiveness of electroconvulsive therapy in treatment-resistant depression: A naturalistic study. *J ECT* 2008;**24**:141–5. doi:10.1097/YCT.0b013e318157ac58

6 Health Quality Ontario. Repetitive Transcranial Magnetic Stimulation for Treatment-Resistant Depression: A Systematic Review and Meta-Analysis of Randomized Controlled Trials. *Ont Health Technol Assess Ser.* 2016;**16**:1–66.

7 Simpson KN, Welch MJ, Kozel FA, *et al.* Cost-effectiveness of transcranial magnetic stimulation in the treatment of major depression: A health economics analysis. *Adv Ther* 2009;**26**:346–68. doi:10.1007/s12325-009-0013-x

8 NICE:IPG542. *Repetitive transcranial magnetic stimulation for depression*.2015.https://www.nice.org.uk/guidance/ipg542

9 Larson J, Munkácsy E. Theta-burst LTP. Brain Res. 2015;**1621**:38–50. doi:10.1016/j.brainres.2014.10.034

10 Berlim MT, McGirr A, Rodrigues Dos Santos N, *et al.* Efficacy of theta burst stimulation (TBS) for major depression: An exploratory meta-analysis of randomized and sham-controlled trials. *J Psychiatr Res* 2017;**90**:102–9. doi:10.1016/j.jpsychires.2017.02.015

11 Li C-T, Chen M-H, Juan C-H, *et al.* Efficacy of prefrontal theta-burst stimulation in refractory depression: a randomized sham-controlled study. *Brain* 2014;**137**:2088–98. doi:10.1093/brain/awu109

12 Blumberger DM, Vila-Rodriguez F, Thorpe KE, *et al.* Effectiveness of theta burst versus high-frequency repetitive transcranial magnetic stimulation in patients with depression (THREE-D): a randomised non-inferiority trial. *Lancet* 2018;**391**:1683–92. doi:10.1016/S0140-6736(18)30295-2.

13 Fox MD, Liu H, Pascual-Leone A. Identification of reproducible individualized targets for treatment of depression with TMS based on intrinsic connectivity. *Neuroimage.* 2013;**66**:151-60. doi: 10.1016/j.neuroimage.2012.10.082.

14. Iwabuchi SJ, Peng D, Fang Y, *et al.* Alterations in effective connectivity anchored on the insula

For peer review only - http://bmjopen.bmj.com/site/about/guidelines.xhtml

in major depressive disorder. *Eur Neuropsychopharmacol* 2014;**24**:1784–92. doi:10.1016/j.euroneuro.2014.08.005

15 Mayberg HS. Limbic-cortical dysregulation: A proposed model of depression. *J. Neuropsychiatry Clin. Neurosci.* 1997;**9**:471–81. doi:10.1176/jnp.9.3.471

16 Sheline YI, Price JL, Yan Z, *et al.* Resting-state functional MRI in depression unmasks increased connectivity between networks via the dorsal nexus. *Proc Natl Acad Sci U S A* 2010;**107**:11020–5. doi:10.1073/pnas.1000446107

17 Mulders PC, van Eijndhoven PF, Schene AH, Beckmann CF, Tendolkar I. Resting-state functional connectivity in major depressive disorder: A review. *Neurosci Biobehav Rev.* 2015;**56**:330-44. doi: 10.1016/j.neubiorev.2015.07.014.

18 Lan MJ, Chhetry BT, Liston C, Mann JJ, Dubin M. Transcranial magnetic stimulation of left dorsolateral prefrontal cortex induces brain morphological changes in regions associated with a treatment resistant major depressive episode: an exploratory analysis. *Brain Stimul*. 2016;**9**:577-83. doi: 10.1016/j.brs.2016.02.011.

19 McGrath CL, Kelley ME, Holtzheimer PE *et al.* Toward a neuroimaging treatment selection biomarker for major depressive disorder. *JAMA Psychiatry*. 2013;**70**:821-9. doi: 10.1001/jamapsychiatry.2013.143.

20 Drysdale AT, Grosenick L, Downar J, *et al.* Resting-state connectivity biomarkers define neurophysiological subtypes of depression. *Nat Med* 2017;**23**:28–38. doi:10.1038/nm.4246

21 Iwabuchi SJ, Auer DP, Lankappa ST, *et al.* Baseline effective connectivity predicts response to repetitive transcranial magnetic stimulation in patients with treatment-resistant depression. *Eur Neuropsychopharmacol* 2019;**29**:681–90. doi:10.1016/j.euroneuro.2019.02.012

22 Iwabuchi SJ, Raschke F, Auer DP, *et al.* Targeted transcranial theta-burst stimulation alters fronto-insular network and prefrontal GABA. *Neuroimage* 2017;**146**:395–403. doi:10.1016/j.neuroimage.2016.09.043

23 Liston C, Chen AC, Zebley BD, *et al.* Default mode network mechanisms of transcranial magnetic stimulation in depression. *Biol Psychiatry* 2014;**76**:517–26. doi:10.1016/j.biopsych.2014.01.023

24 First MB, Williams JBW, Karg RS, *et al*. Structured clinical interview for DSM-5—Research version (SCID-5 for DSM-5, research version; SCID-5-RV). Arlington, Va, American Psychiatric Association 2015

25 American Psychiatric Association. *Diagnostic and statistical manual of mental disorders (DSM-5. 5th edn).* Arlington, VA: American Psychiatric Association Publishing, 2013

26 Fava M. Diagnosis and definition of treatment-resistant depression. *Biol Psychiatry* 2003;**53**:649–59. doi:10.1016/S0006-3223(03)00231-2

27. Williams JBW, Kobak KA, Bech P, *et al.* The GRID-HAMD: standardization of the Hamilton Depression Rating Scale. *Int Clin Psychopharmacol* 2008;**23**:120–9. doi:10.1097/YIC.0b013e3282f948f5

28. Bernstein DP, Fink L, Handelsman L, *et al.* Initial reliability and validity of a new retrospective measure of child abuse and neglect. *Am J Psychiatry* 1994;**151**:1132–6. doi:10.1176/ajp.151.8.1132

29 Beck AT, Steer RA, Brown GK. Manual for Beck Depression Inventory-II. San Antonio, TX:

For peer review only - http://bmjopen.bmj.com/site/about/guidelines.xhtml

Psychological Corporation, 1996

30 Kroenke K, Spitzer RL, Williams JBW. The PHQ-9: Validity of a brief depression severity measure. *J Gen Intern Med* 2001;**16**:606–13. doi:10.1046/j.1525-1497.2001.016009606.x

31 Spitzer RL, Kroenke K, Williams JBW, *et al.* A brief measure for assessing generalized anxiety disorder: The GAD-7. *Arch Intern Med* 2006;**166**:1092–7. doi:10.1001/archinte.166.10.1092

32 Mundt JC, Marks IM, Shear MK, et al. The Work and Social Adjustment Scale: A simple measure of impairment in functioning. Br J Psychiatry 2002;180:461–4. doi:10.1192/bjp.180.5.461

33 Van Hout B, Janssen MF, Feng Y-F et al. Interim Scoring for the EQ-5D-5L: Mapping the EQ-5D-5L to EQ-5D-3L. Value Sets. Value in Health 2012; 15: 708-715. doi: 10.1016/j.jval.2012.02.008

34 Rush AJ, Trivedi MH, Ibrahim HM, et al. The 16-item Quick Inventory of Depressive Symptomatology (QIDS) Clinician Rating (QIDS-C) and Self-Report (QIDS-SR): A psychometric evaluation in patients with chronic major depression. Biol Psychiatry 2003; 54: 573-583. doi: 10.1016/s0006-3223(02)01866-8

35 Beecham J, Knapp MR. Costing psychiatric intervention. In: Thornicroft GJ ed. Measuring mental health needs. Gaskell, 2001

36 Demyttenaere K, Desaiah D, Petit C, et al. Patient-assessed versus physician-assessed disease severity and outcome in patients with nonspecific pain associated with major depressive disorder. Prim Care Companion J Clin Psychiatry 2009;11:8–15. doi:10.4088/pcc.08m00670

37 Zimmerman M, Martinez JH, Young D, *et al.* Severity classification on the Hamilton depression rating scale. *J Affect Disord* 2013;**150**:384–8. doi:10.1016/j.jad.2013.04.028

38 McAllister-Williams RH, Anderson IM, Finkelmeyer A, *et al.* Antidepressant augmentation with metyrapone for treatment-resistant depression (the ADD study): A double-blind, randomised, placebo-controlled trial. *The Lancet Psychiatry* 2016;**3**:117–27. doi:10.1016/S2215-0366(15)00436-8

39 Curtis, L., Burns, A. (2017) Unit Costs of Health and Social Care 2017, Personal Social Services Research Unit, University of Kent, Canterbury. https://doi.org/10.22024/UniKent/01.02/65559

40 Hoch JS, Briggs AH, Willan AR. Something old, something new, something borrowed, something blue: a framework for the marriage of health econometrics and cost-effectiveness analysis. *Health Econ* 2002;**11**:415–30. doi:10.1002/hec.678

For peer review only - http://bmjopen.bmj.com/site/about/guidelines.xhtml

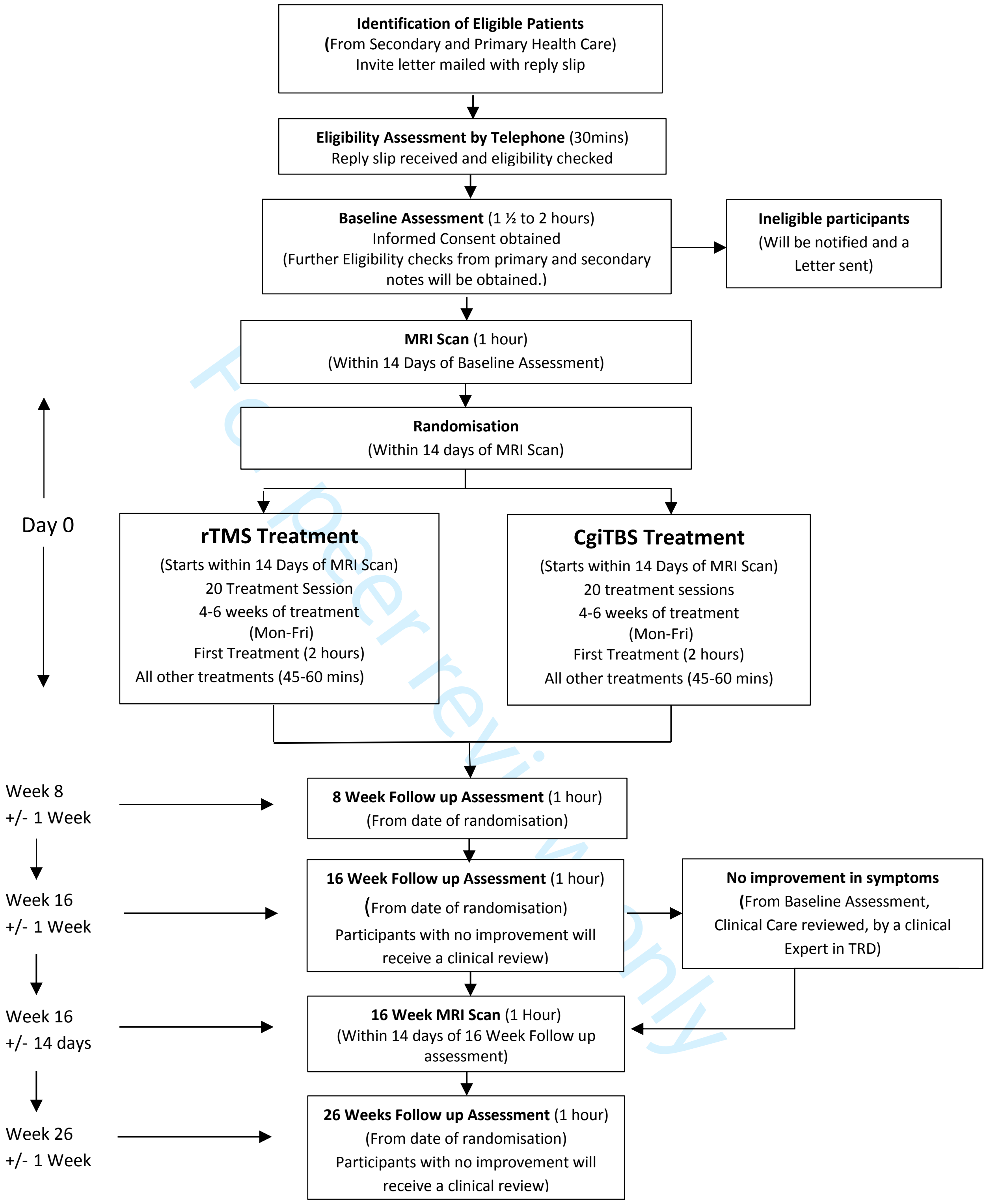

Figure 1. Study Flow Chart

Appendix 1: Assessment of degree of treatment resistance.

The definition and assessment of the degree of treatment resistance depression (TRD) in the BRIGhTMIND study is operationalised using the Massachusetts General Hospital (MGH) TRD scoring system (Fava et al, 2003). This assesses the number of previous biological treatments for depression, given as an adequate treatment trial, in the current episode of depression. The use of psychological treatments is not assessed by this tool because it can be challenging to obtain reliable accounts of structured psychotherapy from participant's accounts (Lobban et al, 2017). To date, the relationship between non-response to psychotherapy and response to TMS has not been investigated. This is in contrast to evidence that the degree of treatment resistance to biological treatments may moderate treatment outcome to TMS (Lee et al, 2012). The use of, and response to, psychological therapy is recorded separately to the MGH TRD score in the BRIGhTMIND study for use as an exploratory variable in relation to predictors of response.

The MGH TRD score is based on one point being scored for every different antidepressant prescribed at the minimum effective dose for a minimum of 6 weeks, without response. An extra 0.5 points is scored if the trial has been at least 10 weeks and if the dose of the antidepressant has been optimised to a defined level. A further 0.5 can be scored if the antidepressant has been augmented by a second drug. Any course of electroconvulsive therapy lasting a minimum of 4 treatment sessions is given a score of 3 points.

The MGH TRD scoring tool used in BRIGhTMIND is an updated version of that published by Fava et al. in 2003, with medications not available at that time added and ones that are no longer, or where never, available in the UK removed (see supplementary table 1). Specifically, the tricyclic antidepressants amoxapine, desipramine, maprotiline and protriptyline, and the "other" antidepressant nefazadone, that were included in the scoring table in Fava et al. (2003), were removed. The tricyclic antidepressants dosulepin (75 and 150mg) lofepramine (140 and 210mg), monoamine oxidase inhibitor moclobemide (300 and 600mg) and "other" antidepressants reboxetine (8 and 12mg), agomelatine (25 and 50mg) and vortioxetine (10 and 20mg) were added with minimum and optimised doses indicated in brackets. These doses were based on the various drugs' Summary of Product Characteristics (SPC) and consensus of the clinicians involved in the setup of BRIGhTMIND.

In the scoring table published by Fava et al. (2003), the definition of which drugs could be counted towards augmenting an antidepressant is vague, simply presenting examples: "e.g., buspirone (Buspar), lithium, psychostimulants such as methylphenidate (Ritalin), atypical antipsychotics such as olanzapine (Zyprexa). It was decided that more precise guidance should be provided. The drugs included are based on consensus of BRIGhTMIND clinicians and British Association for Psychopharmacology (BAP) Guidelines (Cleare et al, 2015). All first and second line BAP options were included (lithium, aripiprazole, quetiapine, risperidone, olanzapine and tri-iodothyronine (T3)). BAP guidelines also include mirtazapine augmentation of another antidepressant. Because of the diverse range of antidepressant combinations sometimes used in clinical practice, "mirtazapine" was broadened to "any second antidepressant". The two remaining drugs specifically mentioned by Fava et al. (2003), buspirone and methylphenidate were retained (i.e. all drugs specifically mentioned

For peer review only

by Fava et al. are included). Two extra drugs were then added by consensus on the basis of awareness of their use in the centres involved in the BRIGhTMIND study: modafinil and pramipexole. These both have some RCT evidence supporting their use as augmentation agents (Goss et al. 2013; Tundo et al. 2019).

In BRIGhTMIND each patient is scored on the adapted MGH TRD scoring system. The inclusion criterion for the RCT is a score of 2 points or more. This is broadly in line with the conventional definition of TRD as being failure to respond to two adequate courses of different antidepressants (Brown et al. 2019): failure to respond to two different antidepressants prescribed at the minimum dose for 6 weeks each would score 2 X 1 point. However, it should be noted that dose optimisation and a treatment trial of a single antidepressant could lead to the same score (1 + 0.5 + 0.5 points).

Scoring the MGD TRD scale is dependent on having information regarding the patient's past treatments during this current episode. In BRIGhTMIND, this is established through a combination of interview with the patient and examination of primary and secondary care case notes. Patients are interviewed using timeline follow back techniques to identify when the current episode of depression started. However, the MGH TRD scores are very susceptible to error due to mis-remembered details and inadequate, inaccurate or inaccessible case notes. Rather than focusing on the specific MGH TRD score, patients in BRIGhTMIND are allocated to three categories of degree of treatment resistance: low, medium and high. These are defined on the basis of data collected in a previous RCT in patients with TRD, the ADD study (McAllister-Williams et al. 2016), by scores of 2-3.5, 4-6 and ≥6.5 respectively. If there is any concern that there is incomplete data on past treatments, then a patient is allocated to the high resistance group.

The specific guidance on using the MGH TRD scoring system in BRIGhTMIND is as follows:

1. The scale assesses the degree of treatment resistance in the CURRENT EPISODE. For some patients with long histories of depression it can be difficult to determine the beginning of an episode. Count a new episode of depression from the end of any period of substantially better mood for a minimum of 2 months.
2. Confirm if ANY antidepressants have been taken (not just prescribed) in the current episode. If not, then the patient is excluded.
3. If the participant has taken any antidepressants in the current episode then collect information regarding WHAT has been taken (all psychotropics), at what DOSE and for how LONG at the minimum dose or greater. Then, using supplementary table 1:
    a. in column A, tick an antidepressant the patient has taken at the minimum dose for at least 6 weeks during THIS episode of depression.
    b. for antidepressants ticked in column A, put another tick in column B if the treatment continued for at least 10 weeks.
    c. tick column C if the patient has taken the drug at a dos equal to or greater than the maximum dosage listed for that medication. *(There is no extra score for doses higher than the maximum)*
    d. If the patient has been prescribed any of the drugs listed here (taken for at least 4 weeks) during the same time period to boost the antidepressant effect, write the name in column D.

For peer review only

i. **NB** – if an antidepressant combination is used, then only score for one of these with the second antidepressant being the augmentation agent. For example if a patient on venlafaxine has mirtazapine added, put a tick in the venlafaxine row and write ‘mirtazapine’ in Column D. Don’t tick the Mirtazapine row (unless this was also used in monotherapy).

ii. **NB** – augmentation agents should in theory be used at minimum effective doses. However, there is a lack of consensus as to what these should be. If in any doubt seek a view of the local Principal Investigators or Chief Investigator.

4. Add the scores:
   a. For each antidepressant add a score to column E. This is +1 for the antidepressant, +0.5 if used for at least 10 weeks (at minimum dose or above), +0.5 if the Maximum dose or greater was used and +0.5 for EACH augmentation agent used.
   b. If the patient has received ECT in this episode, then add 3 into column E on the ECT row at the bottom
   c. Calculate the total MGH TRD score adding up all scores in column E.

For peer review only

Supplementary table 1

Adapted Massachusetts General Hospitals Staging Method to classify treatment resistant depression for the BRIGhTMIND Study.

Has the patient received any treatment with ANTIDEPRESSANT medications since the beginning of THIS CURRENT episode or period of depression?

1 = Yes 2 = No ☐ If NO – Exclude from Study

If Yes

1) On the Grid below in column A, tick an antidepressants the patient has taken at the minimum dose for at least 6 weeks during THIS episode of depression.
2) For antidepressants ticked in column A, put another tick in column B if the treatment continued for at least 10 weeks.
3) Tick column C if the patient has taken the drug at a dose equal to or greater than the maximum dosage listed for that medication. *(There is no extra score for doses higher than the maximum.)*
4) If the patient has been prescribed any of the drugs listed here during the same time period to boost the antidepressant effect, write the name in column D, *Amisulpride, Aripiprazole, Buspirone, Lithium, Methylphenidate, Modafinil, Olanzapine, Pramipexole, Quetiapine, Risperidone, triiodothyronine (T3), and any second Antidepressant.*

**If ticked score**			1	0.5		0.5	0.5	
			A	B		C	D	
	**Generic name**	**Min dose (mg/day)**	**At least 6 wks**	**At least 10 wks**	**Max dose (mg/day)**	**Equal or greater**	**Name of drug added to augment this antidepressant**	**Score**
**Tricyclic antidepressants**	Doxepin	150			250			
	Clomipramine	150			250			
	Amitriptyline	150			250			
	Nortriptyline	75			125			
	Trimipramine	150			250			
	Imipramine	150			250			
	Dosulepin	75			150			
	Lofepramine	140			210			
**MAOIs**	Isocarboxazid	30			60			
	Phenelzine	45			90			
	Tranylcypromine	30			60			
	Moclobemide	300			600			

SSRIs	Fluvoxamine	50			150			
	Paroxetine	20			60			
	Fluoxetine	20			60			
	Sertraline	50			150			
	Citalopram	20			60			
	Escitalopram	10			30			
SNRI	Venlafaxine	125			250			
	Duloxetine	60			100			
Other	Trazodone	300			600			
	Bupropion	300			450			
	Mirtazapine	15			45			
	Reboxetine	8			12			
	Agomelatin	25			50			
	Vortioxetine	10			20			
Did the patient receive electroconvulsive treatment (ECT) during the current episode (Score 3)?								
							Total MGH score	

Appendix 2 Studies on Cognition, depression and effective connectivity

Background

Depression is associated with marked impairments in attention and executive function (Austin et al., 2001; McDermott and Ebmeier, 2009). Part of these impairments could be explained by increased intraindividual variability (IIV) in attentional performance – patients with depression show a skewed distribution of response times on sustained attention tasks, with an increased frequency of slow responses consistent with attentional lapses (Gallagher et al., 2015). This can be analysed by decomposing the distribution of response times into a Gaussian component and an exponential component (Schmiedek et al., 2007) – Gallagher et al. (2015) found the time constant of the exponential component to be a more sensitive measure for differentiating patients from controls than simple mean reaction times.

It may be that attentional lapses reflect interference from brain areas involved in internally directed mental activity and rumination (the so-called "default mode network" of areas, DMN) on areas involved in executive function (the "frontoparietal network" of areas, FPN). Previous work has found that executive function in non-clinical populations is related to the degree of anticorrelation between the activities of the FPN and DMN (Kelly et al., 2008; Keller et al., 2015), suggesting that the ability to separate the activities of these networks may be critical to task performance. One of the key findings of a meta-analysis of resting-state fMRI studies in patients with depression was an increase in connectivity between the FPN and the DMN, suggesting a breakdown of this separability (Kaiser et al., 2015).

It is unclear the extent to which attentional and executive impairments are independent components of the pathophysiology of depression or a secondary consequence of impairments in mood (Bora et al., 2013; Rock et al., 2014). Independence could be demonstrated in a dissociation between improvements in mood and improvements in cognition during treatment. Cheng et al. (2016) provide preliminary evidence for such a dissociation with a selective improvement in executive function in a group receiving intermittent TBS compared to those receiving continuous TBS or a combination of intermittent and continuous TBS.

Methods

Embedded within the BRIGhTMIND study, we will explore correlations between IIV on the THINC-it battery of cognitive tests (measured with the method of Gallagher et al., 2015) and resting-state functional connectivity between the FPN and the DMN. Separate correlations using baseline measures of each variable and using change in each variable from baseline to 16 weeks will be calculated. Analyses will be performed without unblinding TMS treatment allocation group. We predict a positive correlation between IIV and FPN-DMN functional connectivity at baseline, and a positive correlation between change in IIV and change in FPN-DMN connectivity. We will explore the mediating effects of low mood (measured with the QIDS-SR, Rush et al., 2013), and improvements in mood, on these relationships. Additionally, we will examine the longitudinal relationship between mood and IIV in a within-subject design using unblinded QIDS-SR and THINC-it data, to ascertain whether increased IIV is a state or trait property of depression.

For peer review only

References

Austin MP, Mitchell P, Goodwin GM. Cognitive deficits in depression: possible implications for functional neuropathology. *Br J Psychiatry* 2001; **178**:200-6.

Bora E, Harrison BJ, Yücel M, et al. Neurocognitive intra-individual variability in mood disorders: effects on attentional response time distributions. *Psychol Med* 2015; **45**: 2985-97.

Brown S, Rittenbach K, Cheung S, McKean G, MacMaster FP, Clement F. Current and Common Definitions of Treatment-Resistant Depression: Findings from a Systematic Review and Qualitative Interviews. *Can. J. Psychiatry* 2019; **64**: 380–387. https://doi.org/10.1177/0706743719828965

Cheng C-M, Juan C-H, Chen M-H, et al. Different forms of prefrontal theta burst stimulation for executive function of medication-resistant depression: evidence from a randomized sham-controlled study. *Prog Neuro-Psychopharmacol Biol Psychiatry* 2016; **66**: 35-40.

Cleare A, Pariante C, Young A, et al. Evidenced-based guidelines for treating depressive disorders with antidepressants: A revision of the 2008 British Association Psychopharmacology guidelines. J Psychopharmacol 2015;29:459-525.

Fava M. Diagnosis and definition of treatment-resistant depression. *Biol Psychiatry* 2003;**53**:649–59. doi:10.1016/S0006-3223(03)00231-2.

Gallagher P, Nilsson J, Finkelmeyer A et al. Neurocognitive intra-individual variability in mood disorders: effects on attentional response time distributions. *Psychol Med.* 2015;**45**:2985-97. doi: 10.1017/S0033291715000926.

Goss AJ, Kaser M, Costafreda SG, Sahakian BJ, Fu CH. Modafinil augmentation therapy in unipolar and bipolar depression: a systematic review and meta-analysis of randomized controlled trials. *J Clin Psychiatry* 2013; **74**:1101-7. doi: 10.4088/JCP.13r08560.

Kaiser RH, Andrews-Hanna JR, Wager TD, Pizzagalli DA. Large-Scale network dysfunction in major depressive disorder: a meta-analysis of resting-state functional connectivity. *JAMA Psychiatry.* 2015;**72**:603-11. doi: 10.1001/jamapsychiatry.2015.0071.

Keller JB, Hedden T, Thompson TW, Anteraper SA, Gabrieli JD, Whitfield-Gabrieli S. Resting-state anticorrelations between medial and lateral prefrontal cortex: association with working memory, aging, and individual differences. *Cortex.* 2015;**64**:271-80. doi: 10.1016/j.cortex.2014.12.001

Kelly AM, Uddin LQ, Biswal BB et al. Competition between functional brain networks mediates behavioral variability. *Neuroimage.* 2008;**39**:527-37.

Lee JC, Blumberger DM, Fitzgerald PB, Daskalakis ZJ, Levinson AJ. The role of transcranial magnetic stimulation in treatment-resistant depression: a review. Curr Pharm Des. 2012;18:5846-52.

Lobban F, Dodd AL, Sawczuk AP et al. Assessing Feasibility and Acceptability of Web-Based Enhanced Relapse Prevention for Bipolar Disorder (ERPonline): A Randomized Controlled Trial. J Med Internet Res. 2017 Mar 24;19(3):e85. doi: 10.2196/jmir.7008.

McAllister-Williams RH, Anderson IM, Finkelmeyer A, et al. Antidepressant augmentation with metyrapone for treatment-resistant depression (the ADD study): A double-blind, randomised, placebo-controlled trial. *Lancet Psychiatry* 2016; **3**:117–27. doi:10.1016/S2215-0366(15)00436-8

For peer review only

McDermott LM, Ebmeier KP. A meta-analysis of depression severity and cognitive function. *J Affect Disord.* 2009;**119**:1-8. doi: 10.1016/j.jad.2009.04.022

Rock PL, Roiser JP, Riedel WJ, Blackwell AD. Cognitive impairment in depression: a systematic review and meta-analysis. *Psychol Med.* 2014;**44**:2029-40. doi: 10.1017/S0033291713002535.

Rush AJ, Trivedi MH, Ibrahim HM, et al. The 16-item Quick Inventory of Depressive Symptomatology (QIDS) Clinician Rating (QIDS-C) and Self-Report (QIDS-SR): A psychometric evaluation in patients with chronic major depression. *Biol Psychiatry* 2003; **54**: 573-583. doi: 10.1016/s0006-3223(02)01866.

Schmiedek F, Oberauer K, Wilhelm O, et al. Individual differences in components of reaction time distributions and their relations to working memory and intelligence. *J Exp Psychol General* 2007; **136**: 414-429.

Tundo A, de Filippis R, De Crescenzo F. Pramipexole in the treatment of unipolar and bipolar depression. A systematic review and meta-analysis. *Acta Psychiatr Scand* 2019; **140**:116-125. doi: 10.1111/acps.13055.

For peer review only

STANDARD PROTOCOL ITEMS: RECOMMENDATIONS FOR INTERVENTIONAL TRIALS

SPIRIT 2013 Checklist: Recommended items to address in a clinical trial protocol and related documents*

Section/item	Item No	Description	Addressed on page number
**Administrative information**			
Title	1	Descriptive title identifying the study design, population, interventions, and, if applicable, trial acronym	______1_______
Trial registration	2a	Trial identifier and registry name. If not yet registered, name of intended registry	______2_______
	2b	All items from the World Health Organization Trial Registration Data Set	ISRCTN19674644
Protocol version	3	Date and version identifier	_____18________
Funding	4	Sources and types of financial, material, and other support	_____18_______
Roles and responsibilities	5a	Names, affiliations, and roles of protocol contributors	____1,17,18_____
	5b	Name and contact information for the trial sponsor	_____18________
	5c	Role of study sponsor and funders, if any, in study design; collection, management, analysis, and interpretation of data; writing of the report; and the decision to submit the report for publication, including whether they will have ultimate authority over any of these activities	_____18________
	5d	Composition, roles, and responsibilities of the coordinating centre, steering committee, endpoint adjudication committee, data management team, and other individuals or groups overseeing the trial, if applicable (see Item 21a for data monitoring committee)	_____14________

For peer review only - http://bmjopen.bmj.com/site/about/guidelines.xhtml

**Introduction**			
Background and rationale	6a	Description of research question and justification for undertaking the trial, including summary of relevant studies (published and unpublished) examining benefits and harms for each intervention	____3,4________
	6b	Explanation for choice of comparators	____3,4________
Objectives	7	Specific objectives or hypotheses	_____4,7_______
Trial design	8	Description of trial design including type of trial (eg, parallel group, crossover, factorial, single group), allocation ratio, and framework (eg, superiority, equivalence, noninferiority, exploratory)	______5_______
**Methods: Participants, interventions, and outcomes**			
Study setting	9	Description of study settings (eg, community clinic, academic hospital) and list of countries where data will be collected. Reference to where list of study sites can be obtained	______5_______
Eligibility criteria	10	Inclusion and exclusion criteria for participants. If applicable, eligibility criteria for study centres and individuals who will perform the interventions (eg, surgeons, psychotherapists)	______5,6______
Interventions	11a	Interventions for each group with sufficient detail to allow replication, including how and when they will be administered	______6,7______
	11b	Criteria for discontinuing or modifying allocated interventions for a given trial participant (eg, drug dose change in response to harms, participant request, or improving/worsening disease)	_____6,7_______
	11c	Strategies to improve adherence to intervention protocols, and any procedures for monitoring adherence (eg, drug tablet return, laboratory tests)	_____6,7_______
	11d	Relevant concomitant care and interventions that are permitted or prohibited during the trial	______7_______
Outcomes	12	Primary, secondary, and other outcomes, including the specific measurement variable (eg, systolic blood pressure), analysis metric (eg, change from baseline, final value, time to event), method of aggregation (eg, median, proportion), and time point for each outcome. Explanation of the clinical relevance of chosen efficacy and harm outcomes is strongly recommended	______8,9, 11___
Participant timeline	13	Time schedule of enrolment, interventions (including any run-ins and washouts), assessments, and visits for participants. A schematic diagram is highly recommended (see Figure)	______9, Figure 1

For peer review only - http://bmjopen.bmj.com/site/about/guidelines.xhtml

Sample size	14	Estimated number of participants needed to achieve study objectives and how it was determined, including clinical and statistical assumptions supporting any sample size calculations	___13_______
Recruitment	15	Strategies for achieving adequate participant enrolment to reach target sample size	_____8________

Methods: Assignment of interventions (for controlled trials)

Allocation:

Sequence generation	16a	Method of generating the allocation sequence (eg, computer-generated random numbers), and list of any factors for stratification. To reduce predictability of a random sequence, details of any planned restriction (eg, blocking) should be provided in a separate document that is unavailable to those who enrol participants or assign interventions	______10_______
Allocation concealment mechanism	16b	Mechanism of implementing the allocation sequence (eg, central telephone; sequentially numbered, opaque, sealed envelopes), describing any steps to conceal the sequence until interventions are assigned	______10_______
Implementation	16c	Who will generate the allocation sequence, who will enrol participants, and who will assign participants to interventions	______10_______
Blinding (masking)	17a	Who will be blinded after assignment to interventions (eg, trial participants, care providers, outcome assessors, data analysts), and how	______13_______
	17b	If blinded, circumstances under which unblinding is permissible, and procedure for revealing a participant's allocated intervention during the trial	______13_______

Methods: Data collection, management, and analysis

Data collection methods	18a	Plans for assessment and collection of outcome, baseline, and other trial data, including any related processes to promote data quality (eg, duplicate measurements, training of assessors) and a description of study instruments (eg, questionnaires, laboratory tests) along with their reliability and validity, if known. Reference to where data collection forms can be found, if not in the protocol	____8-10______
	18b	Plans to promote participant retention and complete follow-up, including list of any outcome data to be collected for participants who discontinue or deviate from intervention protocols	_______14_____

Data management	19	Plans for data entry, coding, security, and storage, including any related processes to promote data quality (eg, double data entry; range checks for data values). Reference to where details of data management procedures can be found, if not in the protocol	Statistical Analysis Plan___________
Statistical methods	20a	Statistical methods for analysing primary and secondary outcomes. Reference to where other details of the statistical analysis plan can be found, if not in the protocol	__10-12_______
	20b	Methods for any additional analyses (eg, subgroup and adjusted analyses)	___11-12_______
	20c	Definition of analysis population relating to protocol non-adherence (eg, as randomised analysis), and any statistical methods to handle missing data (eg, multiple imputation)	___10-12_______
**Methods: Monitoring**			
Data monitoring	21a	Composition of data monitoring committee (DMC); summary of its role and reporting structure; statement of whether it is independent from the sponsor and competing interests; and reference to where further details about its charter can be found, if not in the protocol. Alternatively, an explanation of why a DMC is not needed	_____14,17_____
	21b	Description of any interim analyses and stopping guidelines, including who will have access to these interim results and make the final decision to terminate the trial	_____None______
Harms	22	Plans for collecting, assessing, reporting, and managing solicited and spontaneously reported adverse events and other unintended effects of trial interventions or trial conduct	_____14________
Auditing	23	Frequency and procedures for auditing trial conduct, if any, and whether the process will be independent from investigators and the sponsor	___By sponsor__
**Ethics and dissemination**			
Research ethics approval	24	Plans for seeking research ethics committee/institutional review board (REC/IRB) approval	______14_______
Protocol amendments	25	Plans for communicating important protocol modifications (eg, changes to eligibility criteria, outcomes, analyses) to relevant parties (eg, investigators, REC/IRBs, trial participants, trial registries, journals, regulators)	___No changes_

For peer review only - http://bmjopen.bmj.com/site/about/guidelines.xhtml

Consent or assent	26a	Who will obtain informed consent or assent from potential trial participants or authorised surrogates, and how (see Item 32)	_______8_______
	26b	Additional consent provisions for collection and use of participant data and biological specimens in ancillary studies, if applicable	_____N/A________
Confidentiality	27	How personal information about potential and enrolled participants will be collected, shared, and maintained in order to protect confidentiality before, during, and after the trial	___Under GCP and Data Protection Act___
Declaration of interests	28	Financial and other competing interests for principal investigators for the overall trial and each study site	_____19__________
Access to data	29	Statement of who will have access to the final trial dataset, and disclosure of contractual agreements that limit such access for investigators	_Statistical Analysis Plan_
Ancillary and post-trial care	30	Provisions, if any, for ancillary and post-trial care, and for compensation to those who suffer harm from trial participation	___None________
Dissemination policy	31a	Plans for investigators and sponsor to communicate trial results to participants, healthcare professionals, the public, and other relevant groups (eg, via publication, reporting in results databases, or other data sharing arrangements), including any publication restrictions	_____14__________
	31b	Authorship eligibility guidelines and any intended use of professional writers	___17,18_______
	31c	Plans, if any, for granting public access to the full protocol, participant-level dataset, and statistical code	Publication of protocol paper and Statistical Analysis Plan
**Appendices**			
Informed consent materials	32	Model consent form and other related documentation given to participants and authorised surrogates	From sponsor
Biological specimens	33	Plans for collection, laboratory evaluation, and storage of biological specimens for genetic or molecular analysis in the current trial and for future use in ancillary studies, if applicable	______N/A_______

For peer review only - http://bmjopen.bmj.com/site/about/guidelines.xhtml

*It is strongly recommended that this checklist be read in conjunction with the SPIRIT 2013 Explanation & Elaboration for important clarification on the items. Amendments to the protocol should be tracked and dated. The SPIRIT checklist is copyrighted by the SPIRIT Group under the Creative Commons "Attribution-NonCommercial-NoDerivs 3.0 Unported" license.

For peer review only

BMJ Open

Connectivity guided theta burst transcranial magnetic stimulation versus repetitive transcranial magnetic stimulation for treatment resistant moderate to severe depression: study protocol for a randomised double-blind controlled trial (BRIGhTMIND).

Journal:	*BMJ Open*
Manuscript ID	bmjopen-2020-038430.R1
Article Type:	Protocol
Date Submitted by the Author:	05-May-2020
Complete List of Authors:	Morriss, Richard; University of Nottingham, Psychiatry<br>Webster, Lucy; Nottinghamshire Healthcare NHS Foundation Trust<br>Abdelghani, Mohamed; Camden and Islington NHS Foundation Trust<br>Auer, Dorothee; University of Nottingham, Arthritis Research UK Pain Centre; University of Nottingham, Sir Peter Mansfield Imaging Centre<br>Barber, Shaun; University of Leicester<br>Bates, Peter<br>Blamire, Andrew; University of Newcastle upon Tyne<br>Briley, Paul; University of Nottingham<br>Brookes, Cassandra; University of Leicester, Leicester Clinical Trials Unit<br>Iwabuchi, Sarina; University of Nottingham<br>James, Marilyn; University of Nottingham, School of Medicine<br>Kaylor-Hughes, Catherine; University of Nottingham<br>Lankappa, Sudheer; Nottinghamshire Healthcare NHS Foundation Trust<br>Liddle, Peter; University of Nottingham<br>McAllister-Williams, Hamish; University of Newcastle upon Tyne<br>O'Neill-Kerr, Alex; Northamptonshire Healthcare NHS Foundation Trust<br>Pszczolkowski Parraguez, Stefan; University of Nottingham, Precision Imaging Beacon<br>Suazo Di Paola, Ana; University of Leicester, Leicester Clinical Trials Unit<br>Thomson, Louise; University of Nottingham, Psychiatry<br>Walters, Yvette; University of Leicester, Leicester Clinical Trials Unit
**Primary Subject Heading**:	Mental health
Secondary Subject Heading:	Radiology and imaging, Health economics
Keywords:	Depression & mood disorders < PSYCHIATRY, Magnetic resonance imaging < RADIOLOGY & IMAGING, Health economics < HEALTH SERVICES ADMINISTRATION & MANAGEMENT, Adult psychiatry < PSYCHIATRY, Clinical trials < THERAPEUTICS

For peer review only - http://bmjopen.bmj.com/site/about/guidelines.xhtml

SCHOLARONE™
Manuscripts

I, the Submitting Author has the right to grant and does grant on behalf of all authors of the Work (as defined in the below author licence), an exclusive licence and/or a non-exclusive licence for contributions from authors who are: i) UK Crown employees; ii) where BMJ has agreed a CC-BY licence shall apply, and/or iii) in accordance with the terms applicable for US Federal Government officers or employees acting as part of their official duties; on a worldwide, perpetual, irrevocable, royalty-free basis to BMJ Publishing Group Ltd ("BMJ") its licensees and where the relevant Journal is co-owned by BMJ to the co-owners of the Journal, to publish the Work in this journal and any other BMJ products and to exploit all rights, as set out in our licence.

The Submitting Author accepts and understands that any supply made under these terms is made by BMJ to the Submitting Author unless you are acting as an employee on behalf of your employer or a postgraduate student of an affiliated institution which is paying any applicable article publishing charge ("APC") for Open Access articles. Where the Submitting Author wishes to make the Work available on an Open Access basis (and intends to pay the relevant APC), the terms of reuse of such Open Access shall be governed by a Creative Commons licence – details of these licences and which Creative Commons licence will apply to this Work are set out in our licence referred to above.

Other than as permitted in any relevant BMJ Author's Self Archiving Policies, I confirm this Work has not been accepted for publication elsewhere, is not being considered for publication elsewhere and does not duplicate material already published. I confirm all authors consent to publication of this Work and authorise the granting of this licence.

For peer review only

Connectivity guided theta burst transcranial magnetic stimulation versus repetitive transcranial magnetic stimulation for treatment resistant moderate to severe depression: study protocol for a randomised double-blind controlled trial (BRIGhTMIND).

Richard Morriss, Professor of Psychiatry, University of Nottingham

Lucy Webster, Research Assistant, Nottinghamshire Healthcare NHS Foundation Trust

Mohamed Abdelghani, Consultant Psychiatrist, Camden and Islington NHS Foundation Trust,

Dorothee Auer, Professor of Neuroimaging, University of Nottingham,

Shaun Barber, medical statistician, University of Leicester

Peter Bates, PPI co-ordinator, University of Nottingham

Andrew Blamire, Professor of Neuroimaging, University of Newcastle

Paul M. Briley, Academic Clinical Fellow, University of Nottingham

Cassandra Brookes, Principal medical statistician, University of Leicester

Sarina Iwabuchi, Research Fellow, University of Nottingham

Marilyn James, Professor of Health Economics, University of Nottingham

Catherine Kaylor-Hughes, Research Fellow, University of Nottingham

Alex O'Neill-Kerr, Consultant Psychiatrist, University of Northampton

Sudheer Lankappa, Consultant Psychiatrist, Nottinghamshire Healthcare NHS Foundation Trust

Peter Liddle, Professor of Psychiatry, University of Nottingham

R. Hamish Mc-Allister-Williams, Professor of Affective Disorders, Newcastle University and Honorary Consultant Psychiatrist, Cumbria, Northumberland, Tyne and Wear NHS Foundation Trust

Stefan Pszczolkowski Parraguez, Rresearch Fellow, University of Nottingham

Ana Suazo Di Paola, Medical Statistician, University of Leicester

Louise Thomson, Senior Research Fellow, University of Nottingham

Yvette Walters, trial manager, University of Leicester

BRIGhTMIND study team- Lorraine Bastick, Rosie Carr, Alison Cartlidge, Harry Clark, William Cottam, Robert De Vai, Linda Davison, John Gledhill, Adele Gregory, Christopher Griffiths, Andrew Hamilton, Delilah Harding, Kelly Heath, Rachel Hobson, Gbeminiyi Ireoluwa, Najat Khalifa, Kate Johnstone, Charlotte Kirkland, Mark Liddle, Jessica Lynch, Neil Nixon, Jehill Parikh, Isabel Reid, Noemi Reiner, Sandra Simpson, Beverley Smith, Tina Sore, Joseph Stone, Carly Taylorson, Rebecca Toney, Claire Turner, Sarah Wilkinson, Andy Willis, Tom Willis

Corresponding author: Professor Richard Morriss, Institute of mental Health, University of Nottingham, Triumph Road, Nottingham, NG7 2TU. E-mail: richard.morriss@nottingham.ac.uk

For peer review only - http://bmjopen.bmj.com/site/about/guidelines.xhtml

Abstract

Introduction The BRIGhTMIND study aims to determine the clinical effectiveness, cost effectiveness and mechanism of action of connectivity guided intermittent Theta Burst Stimulation (cgiTBS) versus standard repetitive Transcranial Magnetic Stimulation (rTMS) in adults with moderate to severe treatment resistant depression.

Methods and analysis The study is a randomised double-blind controlled trial with 1:1 allocation to either 20 sessions of (a) cgiTBS or (b) neuronavigated rTMS not using connectivity guidance. A total of 368 eligible participants with a diagnosis of current unipolar major depressive disorder that is both treatment resistant (defined as scoring 2 or more on the Massachusetts General Hospital (MGH) Staging Score) and moderate to severe (scoring $\geq$16 on the 17-item Hamilton Depression Rating Scale (HDRS-17)), will be recruited from primary and secondary care settings at four treatment centres in the United Kingdom. The primary outcome is depression response at 16 weeks (50% or greater reduction in HDRS-17 score from baseline). Secondary outcomes include assessments of self-rated depression, anxiety, psychosocial functioning, cognition and quality of life at 8, 16 and 26 weeks post randomisation. Cost effectiveness, patient acceptability, safety, mechanism of action and predictors of response will also be examined.

Ethics and dissemination Ethical approval was granted by East Midlands Leicester Central Research Ethics Committee (ref: 18/EM/0232) on 30th August 2018. The results of the study will be published in relevant peer-reviewed journals, and then through professional and public conferences and media. Further publications will explore patient experience, moderators and mediators of outcome and mechanism of action.

Trial registration number ISRCTN19674644; Pre-results.

252 words

Strengths and Limitations of the study

- Large multicentre randomised controlled trial of cgiTBS versus standard rTMS.
- Carefully characterised population of patients with treatment resistant moderate to severe unipolar major depression with broad inclusion and exclusion criteria to enhance generalisability to clinical care.

For peer review only - http://bmjopen.bmj.com/site/about/guidelines.xhtml

- Explores efficacy, sustainability of treatment response, acceptability, cost effectiveness cognitive function, mechanism of action using MRI and spectroscopy, and clinical and MRI predictors of response in the same sample.
- Unable to completely blind sound and sensation of the treatments being tested.
- Both nature of magnetic stimulation (TBS vs rTMS) and stimulation site identification vary between treatment arms.

Introduction

The lifetime prevalence of major depressive disorder (MDD) is approximately 13% of the general population,[1] and it is the most disabling health condition in terms of years lived with disability.[2] Antidepressants and psychotherapies are effective in treating MDD. However, at least 33% of patients in specialist care,[3] and 22% in general primary care,[4] fail to respond to adequate trials of at least two antidepressants. Therefore, it is essential to find alternative treatments for such "treatment-resistant depression" (TRD).

Neuromodulation techniques that attempt to directly modulate the function of targeted brain regions, such as electroconvulsive therapy (ECT),[5] and repetitive transcranial magnetic stimulation (rTMS),[6] can be effective treatments for MDD. rTMS uses powerful magnetic pulses that can be focussed usually on the left dorsolateral prefrontal cortex (DLPFC) to produce changes in brain activity if it is given as a course of treatments. Unlike ECT, rTMS does not require anaesthesia nor produce potential cognitive deficits, and is cost effective in TRD compared with current standard care.[7]

A recent meta-analysis in TRD demonstrated the acute effectiveness of rTMS when compared with sham TMS, showing significant reductions in depression symptoms, and increases in response and remission and response rates, after treatment. [6] However, the beneficial effects of rTMS on mood in TRD may be relatively short, lasting only 1-3 months. [6,8]

Theta burst stimulation (TBS) is an alternative patterned form of TMS, which employs high frequency stimulation, with each treatment administration requiring less time. Unlike rTMS, TBS uses bursts of magnetic pulses that mimic endogenous theta rhythms and it is associated with cortical long-term potentiation that may induce plasticity in more distal brain areas, such as the hippocampus, potentially leading to a higher proportion of responders and a longer duration of effect in TRD.[9] A meta-analysis of five randomised controlled trials (RCTs) in 221 patients with MDD,[10] found unilateral intermittent TBS (iTBS) applied to the left DLPFC and consecutive iTBS applied to the left DLPFC and then continuous TBS applied to the right DLPFC to be robustly effective versus sham TBS

For peer review only - http://bmjopen.bmj.com/site/about/guidelines.xhtml

on depression symptoms and response. One RCT demonstrating 83% of TRD patients given unilateral iTBS maintained their response at 14 weeks unlike sham and bilateral TBS.[11]. A large RCT of 414 TRD patients comparing iTBS versus rTMS to the left dorsolateral prefrontal cortex (DLPFC) showed a non-inferior reduction in depression symptoms after 4-6 weeks of treatment and at 1, 4 and 12 weeks after treatment.[12] Thus, TBS requires a shorter duration of administration than rTMS without compromising clinical effectiveness. Further studies are required to maximise efficacy and durability of effect of TBS, identify its mechanisms of action and predictors of response.

Brain connectivity changes as detected by task-free, resting state functional magnetic resonance imaging (rsfMRI) may individualise neurostimulation therapy of MDD,[13] with the insula suggested as a target for neuromodulation.[14] In depression, an altered network communication exists within and between affective, cognitive control and default mode networks,[15-18] with a disruption of the reciprocal loop between the DLPFC and insula extending to the sensory regions.[16] In addition, hypometabolism at the right anterior insula predicts remission with cognitive behaviour therapy and similarly, hypermetabolism at the right anterior insula predicts remission with antidepressant treatment in patients with MDD.[19] One biotype of MDD, characterised by strong connectivity between the insula and other regions of the brain, was related to partial treatment response to rTMS of at least 25% improvement on depression symptoms in 82.5% participants.[20]

Therefore, in a small pilot study of 27 TRD patients,[21] individualised left DLPFC targets were determined using Granger causality analysis (GCA) to provide a measure of effective functional connectivity (eFC) seeded from the right anterior insula (rAI). Of the 18 participants who completed all follow-up in the two treatment arms (connectivity guided rTMS (cgrTMS) and connectivity guided iTBS (cgiTBS)) higher response rates were found at three-month follow-up favouring cgiTBS (89%) over cgrTMS (44%), indicating the potential for longer lasting efficacy of cgiTBS.

In addition, rsfMRI and magnetic resonance spectroscopy (MRS) may advance neuromodulation therapy through mechanistic evaluation of effects and response predictions. iTBS may dampen fronto-insular eFC in responders with TRD.[21] Left DLPFC GABA levels are increased significantly in rTMS responders versus non-responders.[22] In addition, DLPFC targeted TMS normalises dysfunctional fronto-limbic networks in TRD, with decreased hyperconnectivity between the subgenual anterior cingulate cortex, ventromedial and dorsomedial prefrontal cortex and DLPFC.[23]

iTBS may induce widespread and longer-term network change but precise anatomical localisation of the target circuitry may be required for maximal efficacy of iTBS to normalise dysfunctional fronto-limbic circuitry. Such changes may increase the proportion of people with TRD who obtain a

For peer review only - http://bmjopen.bmj.com/site/about/guidelines.xhtml

sustained response compared to neuronavigated rTMS. Thus, this multi-centre RCT will examine the clinical and cost effectiveness of cgiTBS in comparison with neuronavigated rTMS, in treatment resistant moderate to severe MDD.

Methods and analysis

Study Design

The study is a multicentre parallel group, double blind, randomised, controlled trial of the efficacy of cgiTBS versus neuronavigated rTMS without connectivity guidance, in patients with a primary diagnosis of moderate to severe MDD, which is treatment resistant in their current episode (TRD). The study will be carried out at four sites across UK National Health Services (NHS): Nottinghamshire Healthcare Foundation NHS Trust, Northamptonshire Healthcare NHS Foundation Trust, Cumbria, Northumberland, Tyne and Wear NHS Foundation Trust and Camden and Islington NHS Foundation Trust. The study flow chart (Figure 1) provides an overview of the study design and procedures.

[Insert Figure 1]

Study population and recruitment

368 participants will be recruited in total (184 per treatment arm). Table 1 shows the detailed inclusion/exclusion criteria. Participant identification will occur through primary and secondary care NHS settings across the four sites.

Table 1. Inclusion and Exclusion Criteria

**Inclusion Criteria**	**Exclusion Criteria**
• Adults ≥ 18 years<br>• Diagnosis of current MDD (defined according to the Diagnostic and Statistical Manual of Mental Disorders, Fifth Edition), that is treatment resistant defined as scoring 2 or more on the Massachusetts General Hospital Treatment Resistant Depression staging	• History of bipolar disorder (due to risk of mania) or depression secondary to other mental disorder.<br>• Neurological conditions e.g. brain neoplasm, cerebrovascular events, epilepsy, neurodegenerative disorders, and prior brain surgery.

For peer review only - http://bmjopen.bmj.com/site/about/guidelines.xhtml

score (appendix 1).<br>• Have a 17-Item Hamilton Depression Rating Scale score of 16 or more (moderate to severe depression).<br>• Capacity to provide informed consent before any trial related activities.	• Standard contraindications to MRI i.e. irremovable metal objects in and around body e.g. cardiac pacemaker, implanted medication pump, and pregnancy (any doubt resolved by pregnancy test, women of childbearing age taking precautions against pregnancy) This will include other potential complicated factors such as red tattoos which consist of iron on the head, neck and back and claustrophobia (we offer mock scanner testing and training in some sites).<br>• Major unstable medical illness requiring further investigation or treatment.<br>• Change in prescribed medication 2 weeks before baseline assessment.<br>• Prescription of lamotrigine, gabapentin, pregabalin in the 2 weeks prior to baseline assessment but may be used before then.<br>• Daily prescription of benzodiazepine above 5 mg diazepam equivalents, Zopiclone above 7.5mg, zolpidem above 10mg or Zaleplon above 10mg. These drugs should not be used intermittently in the 2 weeks before baseline assessment but may be used before then.<br>• Current substance abuse or dependence (defined by DSM-5 criteria).<br>• Prior TMS treatment.<br>• At risk of suicidality.<br>• Potential complicated factors relating to the TMS treatment i.e. Hairstyles which would impair magnetic transmission and piercings. (Participants would only be excluded if they chose to not make the changes required to ensure effective treatment.)<br>• Involved with any other clinical trial at the time of consent or 6 months prior.<br>• Unable to read or understand English.

For peer review only

For peer review only - http://bmjopen.bmj.com/site/about/guidelines.xhtml

Interventions

For all treatments, a 70mm figure-of-eight coil (E-z Cool coil) and a Magstim® Horizon® Performance Stimulator with StimGuide® Navigated TMS Package (Magstim Company, Whitland, United Kingdom) will be used. Participants in both treatment arms will receive neuronavigation guided single coil stimulation for 20 TMS sessions delivered over 4-6 weeks. A total of 3000 pulses will be delivered in each rTMS or cgiTBS session. Each treatment session, irrespective of treatment arm, is designed to last approximately 37.5 minutes in total for the purposes of blinding the participants and assessors of outcome. There must not be a gap of more than 4 days between treatments in the course of 20. Medication will be unchanged over 16 weeks follow up unless there is clinical risk of harm.

Individuals assigned to cgiTBS will receive bursts of 3 pulses (80% motor threshold) at 50Hz applied at a frequency of 5 Hz (i.e. every 200 ms) for 40 seconds duration over a site determined from the assessment of maximal strength of effective connectivity between the right anterior insula and the left DLPFC from functional magnetic resonance imaging (fMRI) and structural magnetic resonance imaging (MRI). Neuronavigation computes the nearest location for TBS stimulus on the scalp from an individualised head model based on structural MRI and three fiducial points, the nasion, left preauricular and right preauricular sites. If this site causes discomfort to the participant, it is moved by 1 cm in any direction until there is no discomfort. The pulses are repeated for a total of 5 runs with 5-minute rest intervals between runs.

Individuals assigned to rTMS will follow the standard US Food and Drug Administration (FDA) approved protocol. The site of stimulation will be determined using structural and fMRI utilising neuronavigation to compute the F3 electrode site over the left DLPFC for TMS stimulation from the same head model and three fiducial points. Stimulation is at 120% motor threshold with 75 x 4-second trains of 10Hz interspersed by 26-second intertrain intervals.

Primary Clinical Objective

Our primary hypothesis is that cgiTBS is more efficacious at 16 weeks than standard rTMS in patients with TRD as assessed by the proportion of patients who show a response (50% reduction in depression symptoms from baseline on the HDRS-17).

Secondary Clinical Objectives

For peer review only - http://bmjopen.bmj.com/site/about/guidelines.xhtml

- To explore secondary efficacy outcomes of the mean change in HDRS-17 scores between the two treatment arms and the proportion of patients meeting criteria for remission (HDRS-17 score ≤7) at 16 weeks.
- To explore secondary clinical outcomes of importance to patients and clinicians namely cognition, anxiety, social function and quality of life.
- To examine cost effectiveness of cgiTBS versus rTMS in a UK NHS population.
- To examine the patient acceptability and patient experience of cgiTBS and rTMS.

Mechanistic Objectives

- To investigate the neural mechanism of efficacy in cgiTBS and rTMS.
- To develop response prediction models from brain biotypes and clinical features.

Protocols for the study will be published separately describing the mechanistic objectives and localisation of sites of stimulation in greater detail.

Study Schedule

Trained researchers under the supervision of clinically trained investigators will obtain informed consent and undertake all assessments. A pre-screening questionnaire will be used to telephone screen interested participants first, with potentially eligible participants invited to attend a baseline assessment. Written informed consent will be obtained and study eligibility determined at the baseline assessment.

Information on socio-demographics, past medical and psychiatric history including a detailed assessment of treatment resistance will be obtained from case files and primary care notes. Diagnosis will be assessed by the Structured Clinical Interview (SCID-5)[24] for the Diagnostic and Statistical Manual of Mental Disorders, Fifth Edition (DSM-5).[25]

Treatment resistance will be measured by the Massachusetts General Hospital (MGH) TRD staging score (adapted for new treatment options - see supplementary material, appendix 1).[26]

Depression severity and the primary outcome measure will be assessed using the GRID version of HDRS-17.[27] The HDRS-17 and secondary outcome measures will be assessed in eligible patients at baseline, 8, 16 and 26 weeks post randomisation (Table 2). The primary outcome measure is a binary variable of responder or non-responder at 16 weeks. Individuals observed to have a 50% drop or greater in HDRS-17 from baseline to 16 weeks are defined as responders. In contrast, individuals with <50% reduction or a null value are classified as non-responders. The Childhood Trauma

For peer review only - http://bmjopen.bmj.com/site/about/guidelines.xhtml

Questionnaire (CTQ)[28] will be assessed at baseline as a moderator of treatment response at 16 weeks.

Secondary outcome measures are the Beck Depression Inventory-II (BDI-II),[29] Patient Health Questionnaire (PHQ-9),[30] Generalised Anxiety Disorder Assessment (GAD-7),[31] Work and Social Adjustment Scale (WSAS),[32] EuroQol-5D-5L,[33] and cognitive functioning as measured by the THINC-Integrated Tool (THINC-it) (THINC-it Task Force, http://thinc.progress.im/en). Quick Inventory of Depressive Symptomatology (QIDS-SR) [34] is completed for a mechanism study (see supplementary material – Appendix 2).

In addition, a purposely designed patient proforma will be used to collect patient NHS service utilisation information at the 16 and 26 week follow up time points. This will cover relevant items listed in the Client Service Receipt Inventory (CSRI),[35] and tailor the resources items measured following good practice approaches used by the health economics DiRUM (database of instruments for resource use measurement) group to estimate costs. Patient acceptability will be measured on a 1-5 scale from unacceptable to acceptable and patient experience of overall improvement will be assessed using the Patient Global Impression of Change (1-5 scale much worse to much better)[36] after each TMS session and at each follow up time point. A side effect checklist will be completed after each TMS session and 8 weeks follow up assessment. We will note any changes in medication or other forms of treatment.

For peer review only

Table 2. Study Schedule and Assessments

**Outcome measures**	**Baseline Assessment (Consent to the study)**	**Baseline MRI Scan**	**Treatment Mon-Fri for 4-6 weeks**	**8 Week Follow up Assessment**	**16 Week Follow Up Assessment**	**16 Week MRI Scan**	**26 Week Follow Up Assessment**
Visit Window		Within 14 days of Baseline Assessment	+ 14 days of MRI Scan	+/- 1 week from Randomisation	+/- 1 week from Randomisation	Within 14 days of 16 Week Follow Up Assessment	+/- 1 week from Randomisation
HDRS-17	✓		✓Only if Baseline assessment exceeds 4 weeks	✓	✓		✓
MGH	✓		✓ Only if Baseline assessment exceeds 4 weeks				
BDI-II	✓			✓	✓		✓
PHQ-9	✓			✓	✓		✓
WSAS	✓			✓	✓		✓
GAD7	✓			✓	✓		✓
EQ-5D-5L	✓			✓	✓		✓
THINC-it	✓			✓	✓		✓
SCID-5	✓						
CTQ	✓						
QIDS-SR	✓			✓	✓		✓
Client	✓				✓		✓

Resource Questionnaire							
Patient Acceptability			✓	✓	✓		✓
Side Effects Checklist (Adverse Events)			✓	✓			
fMRI		✓				✓	
MRI		✓				✓	
rsfMRI)		✓				✓	
MRS		✓				✓	
Diffusion weighted imaging		✓				✓	

Participants will undergo baseline structural MRI, resting state fMRI, diffusion weighted imaging scans to assess structural connections between relevant brain regions, and MRS scans (not in London where only fMRI and structural MRI scans will be performed). Treatment target identification will be analysed centrally in Nottingham and communicated to treatment sites. Using GCA of fMRI scans in each subject, coordinates for the stimulation target within the left DLPFC that shows maximal effective connectivity with the right anterior insula will be identified. For the MRS scans, voxels will be placed in the DLPFC and anterior cingulate cortex (ACC), using a similar method to prior work with the MEGA-PRESS sequence for GABA-edited MRS. [22] The MRI scans are repeated at 16 weeks to assess functional connectivity, effective connectivity and GABA.

Randomisation

Randomisation will take place immediately prior to the start of the first treatment session, with participants randomised to either rTMS or cgiTBS treatments. Randomisation will be conducted via a web-based randomisation system (Sealed Envelope, www.sealedevelope.com) by a named TMS nurse practitioner and health care assistant delivering TMS at each centre who will remain un-blinded. Participants will be randomly assigned in a 1:1 ratio into the rTMS and cgiTBS arms using blocks of varying size. Randomisation will be stratified by centre and minimised on severity of depression at baseline and degree of treatment resistance. Baseline depression will be measured by HDRS-17 score and classified as moderate (16-23) or severe (≥24). [37]. Treatment resistance will be measured by the MGH TRD staging score and, based on distributions of treatment resistance in the ADD study,[38], degree of treatment resistance will be classified as low (2-3.5), medium (4-6) and high (≥6.5).

For peer review only

For peer review only - http://bmjopen.bmj.com/site/about/guidelines.xhtml

Statistical Analyses

Efficacy analysis

The primary analysis will test the null hypothesis that treatment with cgiTBS does not increase response rate as measured by HDRS-17 compared to rTMS at 16 weeks on an intent to treat population with any missing response data at 16 weeks being imputed as no response having been achieved. Response at 16 weeks was selected as the primary outcome because of the much larger response rate with cgiTBS than rTMS in our pilot study. [21] A logistic regression will be fitted for the outcome of response with treatment arm, centre, baseline HDRS-17 score and MGH TRD score.

As a secondary analysis a logistic regression model will be repeated in completers (those with 10 or more rTMS or cgiTBS treatments, assessed at baseline and 16 weeks) and a per protocol analysis. Secondary outcomes are the and proportion of responders and remitters at 8, 16 and 26 weeks, and sustained responders at both 16 and 26 weeks will be compared between groups using logistic regression adjusted for treatment centre, baseline HDRS-17 score and MGH TRD score (5% significance). All outcomes are shown in Table 3. Repeated measure linear models will be used for continuous outcomes. Patient acceptability (5-point scale) and safety of both cgiTBS and rTMS (side-effects checklist) will be reported descriptively.

We will explore moderators of depression response at 16 weeks, namely severity of depression by baseline HDRS-17 score, degree of treatment resistance, childhood trauma and age. The number of TMS sessions attended will be explored as a mediator of outcome in exploratory sub-group analyses of the depression response at 16 weeks.

Table 3. Study efficacy outcome measures

Outcomes	Measure	Time Points
Primary Outcome	17-Item Hamilton Depression Rating Scale (HDRS-17)	Response at 16 weeks (defined as by a 50% or greater reduction in HDRS-17 score from baseline)
Secondary Outcomes	HDRS-17	Mean change in HDRS-17 score compared with baseline at 8, 16 and 26 weeks
		Response (as defined above) at 8 weeks and at 26 weeks
		Sustained response at 16 and 26 weeks (defined as a continuing response as defined above following a

For peer review only - http://bmjopen.bmj.com/site/about/guidelines.xhtml

	response at the previous time point)
	Remitters at 8, 16 and 26 weeks (defined as a score of 7 or less on the HDRS-17)
Beck Depression Inventory-II (BDI-II)	At 8, 16 and 26 weeks
Patient Health Questionnaire (PHQ-9)	At 8, 16 and 26 weeks
Quick Inventory of Depressive Symptomatology (QIDS SR)	At 8, 16 and 26 weeks
Generalised Anxiety Disorder Assessment (GAD-7)	At 8, 16 and 26 weeks
THINC Integrated Tool (THINC-it)	At 8, 16 and 26 weeks
EuroQol-5D-5L	At 8, 16 and 26 weeks
Work and Social Adjustment Scale (WSAS)	At 8, 16 and 26 weeks
Patient Acceptability (1-5 scale)	After each TMS session and at 8, 16 and 26 weeks
Adverse Events Checklist	After each TMS session and 8 weeks
MRI scan (structural [T1], diffusion weighted imaging [DWI] for structural connectivity, resting state fMRI for functional connectivity [FC] and effective connectivity [eFC], and GABA MRS of the DLPFC and insula	T1, DWI, FC, eFC at baseline (all centres)<br>GABA at baseline and T1, DWI, FC, eFC, GABA at 16 weeks (all sites except London).

Economic analysis

The economics analysis will take an NHS and personal social services cost perspective in accordance with NICE guidance, including medication, inpatient and outpatient hospital visits and primary and community care use. It will also take a wider societal perspective to capture the broader effects of rTMS and cgiTBS on depression such as paid employment, caring responsibilities and other implications for friends and family. Resource data will then form the units on which cost data, using sources such as the Unit Cost of Health and Social Care\PSSRU,[39] the British National Formulary (BNF), and national reference costs can be attached. The nurse and health care assistant at each centre will complete a diary of time spent managing each participant in the RCT to derive treatment costs for rTMS and cgiTBS. The number of treatment sessions for each treatment arm will be

For peer review only - http://bmjopen.bmj.com/site/about/guidelines.xhtml

For peer review only

carefully recorded and a separate intervention cost assigned to each of the therapies. We will delineate the time spent delivering cgiTBS or rTMS from time spent on research only procedures, such as imaging for delivery of rTMS and additional mechanistic scans in both treatment arms or the extended length of the TBS session for blinding, that would not be used in the real clinical world. The outcome measure for the economic evaluation will be the number of quality-adjusted life year (QALYs) based on a six-month time horizon with no discounting for costs or outcomes as they accrue within a 12-month period. An incremental analysis will be used between the two groups and where appropriate an incremental cost effectiveness ratio (ICER) will be reported between rTMS and cgiTBS. We will use the net monetary benefit framework and implement a net benefit regression,[40] to estimate the extent to which, and the probability that, the cgiTBS intervention is cost-effective compared to standard rTMS at a range of threshold values for the willingness to pay per QALY, generating cost effectiveness acceptability curves (CEACs). Data will be analysed for baseline and centre effects. Key cost drivers will be examined using probabilistic sensitivity analysis.

Qualitative analysis

A purposive sample of 25-30 participants from both arms and all centres, reflecting a mix of demographic characteristics, consent or non-consent to participate, adherence and non-adherence to treatment and follow up will be selected for qualitative interviews, each lasting for up to an hour, after the 16-week assessment (the primary outcome). We will ask about their general views of TMS, benefits from, disadvantages from or dislikes about receiving TMS. Interviews will continue until theme saturation is achieved, i.e. no more themes emerge in subsequent interviews. Qualitative interviews of barriers to recruitment, with staff members involved in recruitment, will also be completed to optimise strategies to improve recruitment as the RCT proceeds. All interviews will be recorded and transcribed verbatim. Inductive thematic analysis using a grounded approach will be adopted.

Sample Size

Sample size is calculated on the minimum clinically important difference in responder rates from baseline to 16 weeks of 15% (equivalent to a number needed to treat (NNT) of 7) between two active treatments (cgiTBS versus rTMS) in favour of the experimental condition cgiTBS. This is an effect size regarded in the literature as a clinically important difference in studies using invasive approaches to manage TRD such as vagal nerve stimulation.[40] The most recent meta-analysis of 16 RCTs in 977 participants with TRD reports a response rate on the HDRS-17 and the Montgomery–

For peer review only - http://bmjopen.bmj.com/site/about/guidelines.xhtml

Åsberg Depression Rating Scale of 26.5% for rTMS at the end of treatment versus 13.1% for sham TMS.[6] Assuming a response rate on the HDRS-17 of 26.5% with rTMS and 41.5% with cgiTBS, 368 patients in total (184 per arm) will provide a power of 80% to detect the difference in response rates at a 5% significance level (two-tailed), allowing for 15% loss to follow-up at 16 weeks. [6]

Blinding

Patients, referring clinical teams and the outcomes assessors will be kept blind with respect to the treatment protocol assigned and administered. Any unintended unblinding of outcome assessors will be recorded and another assessor will complete all further assessments for that participant. At each assessment, the outcomes assessor will be asked to guess the treatment allocation of the participant.

Ethics and dissemination

Ethical Considerations

Ethical approval was granted by East Midlands Leicester Central Research Ethics Committee (ref: 18/EM/0232) on 30/08/2018. The study was registered on 02/10/2018 (ISRCTN19674644) under the public title "BRIGhTMIND: brain imaging guided transcranial magnetic stimulation in depression". The first participant was randomised on 22/01/19. Study finish is scheduled for September 2021. Overview of the study is performed by an external independent Data Monitoring Ethics Committee reporting to a Trial Steering Committee.

Expenses will be covered for participation in the study along with a £10.00 shopping voucher at 16 and 26 Week follow up assessments as a mark of respect and gratitude for the time and input of the participants to the follow up aspects of the trial.

Safety Considerations

Internationally agreed definitions of adverse events (any untoward medical occurrence in a clinical trial subject administered TMS whether or not it has a causal relationship with TMS) and serious

For peer review only - http://bmjopen.bmj.com/site/about/guidelines.xhtml

adverse events (any adverse event or adverse reaction that results in death, is life-threatening, requires hospitalisation or prolongation of existing hospitalisation, results in persistent or significant disability or incapacity, or is a congenital anomaly or birth defect). All participants will be asked about adverse events after every treatment (immediately and a maximum of 72 hours later) until they are resolved. Any participant found to be at risk to themselves (suicide, neglect) or others, or developing a serious adverse event will be referred to relevant clinical services. A review by a clinical expert in TRD will be offered to any participant whose depression has become more severe at 16 and 26 weeks for safety reasons.

Dissemination

All participants will be sent a report summary of the results. Results of the study will be published in peer-reviewed academic journals and communicated widely to academic, profession and public audiences through conferences, media and social media.

Patient and public involvement.

Regarding this study a co-production approach has been taken. We worked with the Involvement Centre at Nottinghamshire Healthcare Trust to create a Magnetic Stimulation Advisory Group who co-produced the treatment pathway for prior pilot work [21] and the current study.

During the study, each centre will be invited to contribute at least two Patient and Public Involvement (PPI) representatives to form a Lived Experience Advisory Panel (LEAP) who will contribute to all study meetings and a representative will be invited to attend all research meetings. At each site PPI representatives will seek opinions, test ideas and gain support for the study. Participant information sheets and other study materials will be co-designed. They will see oversee the interpretation of our findings, particularly the emerging qualitative analysis on barriers to recruitment and patient acceptability of TMS. The PPI process will be overseen by an experienced PPI lead who has trained supported and mentored many PPI groups over recent years. The voice of experts by experience will be heard in all dissemination activities, including presentations or publications.

Discussion

For peer review only - http://bmjopen.bmj.com/site/about/guidelines.xhtml

Although TMS is an established efficacious and safe treatment approach for TRD, research is required to establish if it is possible to increase the proportion of people with TRD who derive a sustained improvement in depression response. [8] Based on theoretical and preliminary empirical evidence, this study explores the potential of using precise personalised neuroanatomical stimulation at a site where affective, cognitive control and default mode networks overlap in neuroanatomical space as determined by MRI derived maximal strength of connectivity between the right anterior insula and the left DLPFC. It will be compared with standard rTMS in a large multicentre randomised controlled trial. Both rTMS and iTBS are effective against sham treatments [6, 8, 10] and recommended for clinical practice in the UK. [8] Furthermore, standard iTBS is non-inferior but not superior to rTMS. [12] Thus, if the current RCT showed that cgiTBS was superior in efficacy, it would be reasonable to conclude that the increased efficacy was due to the personalisation of the site of iTBS treatment.

The design of the trial aims to have high external validity and generalisability to inform routine clinical care. Therefore, inclusion and exclusion criteria are broad and confined to ensuring that safety, efficacy and the investigation of mechanism of action are not compromised. Our definition of TRD [26] is one of the most widely used but it does not consider psychological treatment and underestimates treatment resistance in those who refuse or are not offered antidepressants or ECT in the current episode because of lack of effectiveness or intolerance in previous depression episodes. The broad inclusion and exclusion criteria, large sample size, inclusion of clinical and MRI variables and long duration of follow up will permit a wide variation in potential predictors and non-predictors of response for clinical practice and further research.

Compromises have been made to the design of the study to improve blinding. cgiTBS and rTMS have been matched procedurally so both use the same MRI and neuronavigation system, although these are not currently being used in clinical practice in UK. TMS treatments have also been matched in terms of pulses received and duration of treatment, although the duration of cgiTBS is delivered over a much longer duration but at a lower motor threshold than the United States Federal Drug Agency protocol used in the Three-D study. [12] We decided to use the motor threshold that was well tolerated in our pilot study. [21] We also did not include a third arm of connectivity guided rTMS because our pilot study showed a short duration of response similar to standard rTMS [21]. The use of sham coils to mask the sound and sensation of iTBS and rTMS was explored but effective shams were not available. As a result, entry into the study is restricted to people who had never previously received TMS.

For peer review only

For peer review only - http://bmjopen.bmj.com/site/about/guidelines.xhtml

A co-productive approach has been taken with our patient and public LEAP group and this has been particularly important around informing participants accurately about the study and managing participant expectations. By doing this we hope to optimise recruitment and retention to the study.

In many countries, 3 Tesla MRI is increasingly available so that cgiTBS could be implemented into routine care for TRD. Should the RCT demonstrate that cgiTBS is clinically effective at 16 weeks and cost effective compared to rTMS, then the possibility of maintaining participants with one full course of cgiTBS with booster sessions two or three times per year using the same site of stimulation from a baseline MRI might be feasible. If it is not effective, then other parameters of delivering TMS should be the focus of research and development.

Abbreviations

ACC: anterior cingulate cortex; BDI: Beck Depression Inventory; BNF: British National Formulary; CEACs; Cost effectiveness acceptability curves; cgiTBS: Connectivity Guided Intermitted Theta Burst Stimulation; cgrTMS: Connectivity Guided repetitive Transcranial Magnetic Stimulation; CRF: Case Report Forms; CSRI: Client Service Receipt Inventory; cTBS: continuous Theta Burst Stimulation; CTQ: Childhood Trauma Questionnaire; DiRUM: Data of instrument for resource use measurement; DLPFC: Dorsolateral Prefrontal Cortex; DMPFC: Dorsomedial Prefrontal Cortex; DSM-5: The Diagnostic and Statistical Manual of Mental Disorders, Fifth Edition; ECT: Electroconvulsive Therapy; eFC: Effective Functional Connectivity; FC: Functional Connectivity; FDA: Food and Drug Administration; fMRI: Functional Magnetic Resonance Imaging; GABA: Gamma-amino butyric acid; GAD-7: Generalised Anxiety Disorder Assessment; GCA: Granger Causality Analysis; glx: glutamate and glutamine; GRID-HAMD: 17-Item Hamilton Depression Rating Scale GRID form; HDRS-17: 17-Item Hamilton Depression Rating Scale; ICER; Incremental cost effectiveness ratio; iTBS: intermittent Theta Burst Stimulation; MADRS: The Montgomery–Åsberg Depression Rating Scale; MDD: Major Depressive Disorder; MGH: Massachusetts General Hospital; MRI: Magnetic Resonance Imaging; MRS: Magnetic resonance spectroscopy; NHS: National Health Services; NICE: National Institute of Health and Care Excellence; NNT: Number need to treat; PHQ-9: Patient Health Questionnaire; QALY's: Quality-adjusted life year; rAI: right anterior insula; RCT: Randomised Controlled Trials; rsfMRI: resting state Functional magnetic resonance imaging; rTMS: repetitive Transcranial Magnetic Stimulation; SCID-5: Structured Clinical Interview for DSM-5; TBS: Theta Burst Stimulation; THINC-it: THINC Integrated Tool; TMS: Transcranial Magnetic Stimulation; TRD: Treatment Resistant Depression; WSAS: Work and Social Adjustment Scale

Acknowledgements

We thank the ongoing support of the Funders, the Patient and Public Involvement representatives, participants, research and treatment staff at the four centres, the NIHR Clinical Research Network in East Midlands, North East and North Thames, the clinical, methodological and lay members of the

For peer review only - http://bmjopen.bmj.com/site/about/guidelines.xhtml

independent Data Monitoring Ethics Committee and Trial Steering Committees, the Leicester Clinical Trials Unit and all other staff whom are involved in the study. RM is supported by the Nottingham National Institute for Health Research (NIHR) Biomedical Research Centre, NIHR MindTech Med Tech and in Vitro Centre and NIHR Applied Research Centre East Midlands.

Authors' contributions

All authors approved the final manuscript.

Richard Morriss is the chief investigator and guarantor of the project. He designed the study, obtained funding and wrote the final draft of the protocol.

Lucy Webster wrote operational guidelines for the project, qualitative analysis protocols and the first draft on this manuscript.

Mohammed Abdelghani is the principal investigator for the London site, obtained funding, and designed the TMS schedules.

Dorothee Auer is the neuroimaging lead, led some of the underpinning work designed the neuroimaging and obtained grant funding.

Shaun Barber carried out power calculations for the study and designed the statistical analysis

Peter Bates has led the patient and public involvement aspects of the project and their contributions to the operation of the project.

Andrew Blamire designed the neuroimaging and obtained grant funding.

Paul Briley designed some of the neuroimaging protocol and is analysing the imaging data

Cassandra Brookes designed the statistical analysis and obtained funding for the study.

Sarina Iwabuchi led some of the underpinning work, designed the neuroimaging and TMS parts of the protocol and obtained funding.

Marilyn James designed the health economic analysis, the health economic data collection forms and will be responsible for the health economic management of the trial and oversee all the health economic analysis.

Catherine Kaylor-Hughes wrote protocols for TMS in the study and carried out some of the neuroimaging analysis

Alex Kerr is the principal investigator for the Northampton site, obtained funding, and designed the TMS schedules.

Sudheer Lankappa, the principal investigator for the Nottingham site, carried out some of the underpinning work, obtained funding, and designed the TMS schedules.

For peer review only - http://bmjopen.bmj.com/site/about/guidelines.xhtml

Peter Liddle carried out some of the underpinning work and designed some of the neuroimaging and TMS work, and obtained funding.

Hamish Mc-Allister-Williams is the principal investigator for the Newcastle site, designed the cognitive assessment and assessment of treatment resistance and obtained funding

Stefan Pszczolkowski designed and carried out the computer programming for TMS site location, helped design the Neuronavigation and TMS protocols, and is carrying out neuroimaging analysis.

Ana Suazo Di Paola designed the Statistical Analysis Plan and is carrying out the statistical analysis.

Louise Thomson is the qualitative research lead, designed the qualitative parts of the project and obtained funding.

Yvette Walters is the trial manager of the study and has led the writing of study operating procedures, ethics and governance protocols for the study.

Protocol version

Version 4.0 22/01/2020

Funding

This project (project reference 16/44/22) is funded by the Efficacy and Mechanism Evaluation (EME) Programme, an MRC and NIHR partnership. The views expressed in this publication are those of the author(s) and not necessarily those of the MRC, NIHR or the Department of Health and Social Care. Equipment for the study was provided by Magstim Company Ltd

Sponsor

Research and Evidence Team, Nottinghamshire Healthcare NHS Foundation Trust. Contact e-mail: research@nottshc.nhs.uk. Neither the study sponsor nor funder has or will have any role in designing, collecting, analysing or interpreting the data or any study reports.

Competing Interests

Alex Kerr has received fees for consultancy with Magstim. No other competing interests declared.

References

1 Kessler RC, Bromet EJ. The epidemiology of depression across cultures. *Annu Rev Public Health.* 2013;34:119-38. doi: 10.1146/annurev-publhealth-031912-114409.

2 World Health Organisation. *Depression and Other Common Mental Disorders: Global Health Estimates.* Geneva: World Health Organization; 2017. Licence: CC BY-NC-SA 3.0

3 Rush AJ, Trivedi MH, Wisniewski SR, *et al.* Acute and longer-term outcomes in depressed outpatients requiring one or several treatment steps: A STAR*D report. *Am J Psychiatry* 2006;**163**:1905–17. doi:10.1176/ajp.2006.163.11.1905

For peer review only - http://bmjopen.bmj.com/site/about/guidelines.xhtml

4 Rizvi SJ, Grima E, Tan M, *et al.* Treatment-resistant depression in primary care across Canada. *Can J Psychiatry* 2014;**59**:349–57. doi:10.1177/070674371405900702

5 Khalid N, Atkins M, Tredget J, *et al.* The effectiveness of electroconvulsive therapy in treatment-resistant depression: A naturalistic study. *J ECT* 2008;**24**:141–5. doi:10.1097/YCT.0b013e318157ac58

6 Health Quality Ontario. Repetitive Transcranial Magnetic Stimulation for Treatment-Resistant Depression: A Systematic Review and Meta-Analysis of Randomized Controlled Trials. *Ont Health Technol Assess Ser.* 2016;**16**:1–66.

7 Simpson KN, Welch MJ, Kozel FA, *et al.* Cost-effectiveness of transcranial magnetic stimulation in the treatment of major depression: A health economics analysis. *Adv Ther* 2009;**26**:346–68. doi:10.1007/s12325-009-0013-x

8 NICE:IPG542. *Repetitive transcranial magnetic stimulation for depression*.2015.https://www.nice.org.uk/guidance/ipg542

9 Larson J, Munkácsy E. Theta-burst LTP. Brain Res. 2015;**1621**:38–50. doi:10.1016/j.brainres.2014.10.034

10 Berlim MT, McGirr A, Rodrigues Dos Santos N, *et al.* Efficacy of theta burst stimulation (TBS) for major depression: An exploratory meta-analysis of randomized and sham-controlled trials. *J Psychiatr Res* 2017;**90**:102–9. doi:10.1016/j.jpsychires.2017.02.015

11 Li C-T, Chen M-H, Juan C-H, *et al.* Efficacy of prefrontal theta-burst stimulation in refractory depression: a randomized sham-controlled study. *Brain* 2014;**137**:2088–98. doi:10.1093/brain/awu109

12 Blumberger DM, Vila-Rodriguez F, Thorpe KE, *et al.* Effectiveness of theta burst versus high-frequency repetitive transcranial magnetic stimulation in patients with depression (THREE-D): a randomised non-inferiority trial. *Lancet* 2018;**391**:1683–92. doi:10.1016/S0140-6736(18)30295-2.

13 Fox MD, Liu H, Pascual-Leone A. Identification of reproducible individualized targets for treatment of depression with TMS based on intrinsic connectivity. *Neuroimage.* 2013;**66**:151-60. doi: 10.1016/j.neuroimage.2012.10.082.

14. Iwabuchi SJ, Peng D, Fang Y, *et al.* Alterations in effective connectivity anchored on the insula in major depressive disorder. *Eur Neuropsychopharmacol* 2014;**24**:1784–92. doi:10.1016/j.euroneuro.2014.08.005

15 Mayberg HS. Limbic-cortical dysregulation: A proposed model of depression. *J. Neuropsychiatry Clin. Neurosci.* 1997;**9**:471–81. doi:10.1176/jnp.9.3.471

16 Sheline YI, Price JL, Yan Z, *et al.* Resting-state functional MRI in depression unmasks increased connectivity between networks via the dorsal nexus. *Proc Natl Acad Sci U S A* 2010;**107**:11020–5. doi:10.1073/pnas.1000446107

17 Mulders PC, van Eijndhoven PF, Schene AH, Beckmann CF, Tendolkar I. Resting-state functional connectivity in major depressive disorder: A review. *Neurosci Biobehav Rev.* 2015;**56**:330-44. doi: 10.1016/j.neubiorev.2015.07.014.

18 Lan MJ, Chhetry BT, Liston C, Mann JJ, Dubin M. Transcranial magnetic stimulation of left dorsolateral prefrontal cortex induces brain morphological changes in regions associated with a treatment resistant major depressive episode: an exploratory analysis. *Brain Stimul.* 2016;**9**:577-83. doi: 10.1016/j.brs.2016.02.011.

For peer review only

For peer review only - http://bmjopen.bmj.com/site/about/guidelines.xhtml

19 McGrath CL, Kelley ME, Holtzheimer PE *et al.* Toward a neuroimaging treatment selection biomarker for major depressive disorder. *JAMA Psychiatry*. 2013;**70**:821-9. doi: 10.1001/jamapsychiatry.2013.143.

20 Drysdale AT, Grosenick L, Downar J, *et al.* Resting-state connectivity biomarkers define neurophysiological subtypes of depression. *Nat Med* 2017;**23**:28–38. doi:10.1038/nm.4246

21 Iwabuchi SJ, Auer DP, Lankappa ST, *et al.* Baseline effective connectivity predicts response to repetitive transcranial magnetic stimulation in patients with treatment-resistant depression. *Eur Neuropsychopharmacol* 2019;**29**:681–90. doi:10.1016/j.euroneuro.2019.02.012

22 Iwabuchi SJ, Raschke F, Auer DP, *et al.* Targeted transcranial theta-burst stimulation alters fronto-insular network and prefrontal GABA. *Neuroimage* 2017;**146**:395–403. doi:10.1016/j.neuroimage.2016.09.043

23 Liston C, Chen AC, Zebley BD, *et al.* Default mode network mechanisms of transcranial magnetic stimulation in depression. *Biol Psychiatry* 2014;**76**:517–26. doi:10.1016/j.biopsych.2014.01.023

24 First MB, Williams JBW, Karg RS, *et al*. Structured clinical interview for DSM-5—Research version (SCID-5 for DSM-5, research version; SCID-5-RV). Arlington, Va, American Psychiatric Association 2015

25 American Psychiatric Association. *Diagnostic and statistical manual of mental disorders (DSM-5. 5th edn).* Arlington, VA: American Psychiatric Association Publishing, 2013

26 Fava M. Diagnosis and definition of treatment-resistant depression. *Biol Psychiatry* 2003;**53**:649–59. doi:10.1016/S0006-3223(03)00231-2

27. Williams JBW, Kobak KA, Bech P, *et al.* The GRID-HAMD: standardization of the Hamilton Depression Rating Scale. *Int Clin Psychopharmacol* 2008;**23**:120–9. doi:10.1097/YIC.0b013e3282f948f5

28. Bernstein DP, Fink L, Handelsman L, *et al.* Initial reliability and validity of a new retrospective measure of child abuse and neglect. *Am J Psychiatry* 1994;**151**:1132–6. doi:10.1176/ajp.151.8.1132

29 Beck AT, Steer RA, Brown GK. Manual for Beck Depression Inventory-II. San Antonio, TX: Psychological Corporation, 1996

30 Kroenke K, Spitzer RL, Williams JBW. The PHQ-9: Validity of a brief depression severity measure. *J Gen Intern Med* 2001;**16**:606–13. doi:10.1046/j.1525-1497.2001.016009606.x

31 Spitzer RL, Kroenke K, Williams JBW, *et al.* A brief measure for assessing generalized anxiety disorder: The GAD-7. *Arch Intern Med* 2006;**166**:1092–7. doi:10.1001/archinte.166.10.1092

32 Mundt JC, Marks IM, Shear MK, et al. The Work and Social Adjustment Scale: A simple measure of impairment in functioning. Br J Psychiatry 2002;180:461–4. doi:10.1192/bjp.180.5.461

33 Van Hout B, Janssen MF, Feng Y-F et al. Interim Scoring for the EQ-5D-5L: Mapping the EQ-5D-5L to EQ-5D-3L. Value Sets. Value in Health 2012; 15: 708-715. doi: 10.1016/j.jval.2012.02.008

34 Rush AJ, Trivedi MH, Ibrahim HM, et al. The 16-item Quick Inventory of Depressive Symptomatology (QIDS) Clinician Rating (QIDS-C) and Self-Report (QIDS-SR): A psychometric evaluation in patients with chronic major depression. Biol Psychiatry 2003; 54: 573-583. doi:

For peer review only

For peer review only - http://bmjopen.bmj.com/site/about/guidelines.xhtml

10.1016/s0006-3223(02)01866-8

35 Beecham J, Knapp MR. Costing psychiatric intervention. In: Thornicroft GJ ed. Measuring mental health needs. Gaskell, 2001

36 Demyttenaere K, Desaiah D, Petit C, et al. Patient-assessed versus physician-assessed disease severity and outcome in patients with nonspecific pain associated with major depressive disorder. Prim Care Companion J Clin Psychiatry 2009;11:8–15. doi:10.4088/pcc.08m00670

37 Zimmerman M, Martinez JH, Young D, *et al.* Severity classification on the Hamilton depression rating scale. *J Affect Disord* 2013;**150**:384–8. doi:10.1016/j.jad.2013.04.028

38 McAllister-Williams RH, Anderson IM, Finkelmeyer A, *et al.* Antidepressant augmentation with metyrapone for treatment-resistant depression (the ADD study): A double-blind, randomised, placebo-controlled trial. *The Lancet Psychiatry* 2016;**3**:117–27. doi:10.1016/S2215-0366(15)00436-8

39 Curtis, L., Burns, A. (2017) Unit Costs of Health and Social Care 2017, Personal Social Services Research Unit, University of Kent, Canterbury. https://doi.org/10.22024/UniKent/01.02/65559

40 Hoch JS, Briggs AH, Willan AR. Something old, something new, something borrowed, something blue: a framework for the marriage of health econometrics and cost-effectiveness analysis. *Health Econ* 2002;**11**:415–30. doi:10.1002/hec.678

For peer review only

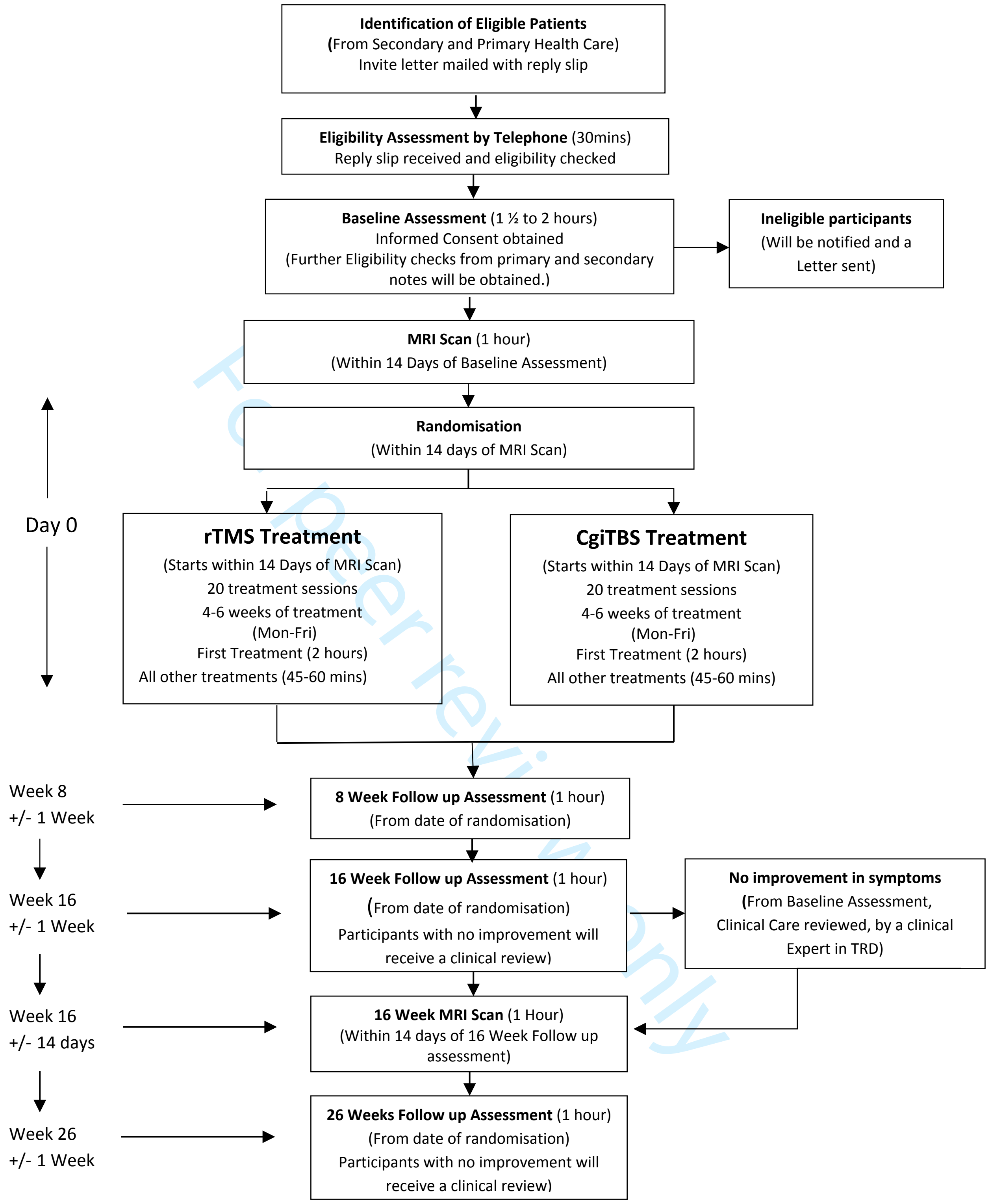

Figure 1. Study Flow Chart

Appendix 1: Assessment of degree of treatment resistance.

The definition and assessment of the degree of treatment resistance depression (TRD) in the BRIGhTMIND study is operationalised using the Massachusetts General Hospital (MGH) TRD scoring system (Fava et al, 2003). This assesses the number of previous biological treatments for depression, given as an adequate treatment trial, in the current episode of depression. The use of psychological treatments is not assessed by this tool because it can be challenging to obtain reliable accounts of structured psychotherapy from participant's accounts (Lobban et al, 2017). To date, the relationship between non-response to psychotherapy and response to TMS has not been investigated. This is in contrast to evidence that the degree of treatment resistance to biological treatments may moderate treatment outcome to TMS (Lee et al, 2012). The use of, and response to, psychological therapy is recorded separately to the MGH TRD score in the BRIGhTMIND study for use as an exploratory variable in relation to predictors of response.

The MGH TRD score is based on one point being scored for every different antidepressant prescribed at the minimum effective dose for a minimum of 6 weeks, without response. An extra 0.5 points is scored if the trial has been at least 10 weeks and if the dose of the antidepressant has been optimised to a defined level. A further 0.5 can be scored if the antidepressant has been augmented by a second drug. Any course of electroconvulsive therapy lasting a minimum of 4 treatment sessions is given a score of 3 points.

The MGH TRD scoring tool used in BRIGhTMIND is an updated version of that published by Fava et al. in 2003, with medications not available at that time added and ones that are no longer, or where never, available in the UK removed (see supplementary table 1). Specifically, the tricyclic antidepressants amoxapine, desipramine, maprotiline and protriptyline, and the "other" antidepressant nefazadone, that were included in the scoring table in Fava et al. (2003), were removed. The tricyclic antidepressants dosulepin (75 and 150mg) lofepramine (140 and 210mg), monoamine oxidase inhibitor moclobemide (300 and 600mg) and "other" antidepressants reboxetine (8 and 12mg), agomelatine (25 and 50mg) and vortioxetine (10 and 20mg) were added with minimum and optimised doses indicated in brackets. These doses were based on the various drugs' Summary of Product Characteristics (SPC) and consensus of the clinicians involved in the setup of BRIGhTMIND.

In the scoring table published by Fava et al. (2003), the definition of which drugs could be counted towards augmenting an antidepressant is vague, simply presenting examples: "e.g., buspirone (Buspar), lithium, psychostimulants such as methylphenidate (Ritalin), atypical antipsychotics such as olanzapine (Zyprexa). It was decided that more precise guidance should be provided. The drugs included are based on consensus of BRIGhTMIND clinicians and British Association for Psychopharmacology (BAP) Guidelines (Cleare et al, 2015). All first and second line BAP options were included (lithium, aripiprazole, quetiapine, risperidone, olanzapine and tri-iodothyronine (T3)). BAP guidelines also include mirtazapine augmentation of another antidepressant. Because of the diverse range of antidepressant combinations sometimes used in clinical practice, "mirtazapine" was broadened to "any second antidepressant". The two remaining drugs specifically mentioned by Fava et al. (2003), buspirone and methylphenidate were retained (i.e. all drugs specifically mentioned

For peer review only

by Fava et al. are included). Two extra drugs were then added by consensus on the basis of awareness of their use in the centres involved in the BRIGhTMIND study: modafinil and pramipexole. These both have some RCT evidence supporting their use as augmentation agents (Goss et al. 2013; Tundo et al. 2019).

In BRIGhTMIND each patient is scored on the adapted MGH TRD scoring system. The inclusion criterion for the RCT is a score of 2 points or more. This is broadly in line with the conventional definition of TRD as being failure to respond to two adequate courses of different antidepressants (Brown et al. 2019): failure to respond to two different antidepressants prescribed at the minimum dose for 6 weeks each would score 2 X 1 point. However, it should be noted that dose optimisation and a treatment trial of a single antidepressant could lead to the same score (1 + 0.5 + 0.5 points).

Scoring the MGD TRD scale is dependent on having information regarding the patient's past treatments during this current episode. In BRIGhTMIND, this is established through a combination of interview with the patient and examination of primary and secondary care case notes. Patients are interviewed using timeline follow back techniques to identify when the current episode of depression started. However, the MGH TRD scores are very susceptible to error due to mis-remembered details and inadequate, inaccurate or inaccessible case notes. Rather than focusing on the specific MGH TRD score, patients in BRIGhTMIND are allocated to three categories of degree of treatment resistance: low, medium and high. These are defined on the basis of data collected in a previous RCT in patients with TRD, the ADD study (McAllister-Williams et al. 2016), by scores of 2-3.5, 4-6 and ≥6.5 respectively. If there is any concern that there is incomplete data on past treatments, then a patient is allocated to the high resistance group.

The specific guidance on using the MGH TRD scoring system in BRIGhTMIND is as follows:

1. The scale assesses the degree of treatment resistance in the CURRENT EPISODE. For some patients with long histories of depression it can be difficult to determine the beginning of an episode. Count a new episode of depression from the end of any period of substantially better mood for a minimum of 2 months.
2. Confirm if ANY antidepressants have been taken (not just prescribed) in the current episode. If not, then the patient is excluded.
3. If the participant has taken any antidepressants in the current episode then collect information regarding WHAT has been taken (all psychotropics), at what DOSE and for how LONG at the minimum dose or greater. Then, using supplementary table 1:
    a. in column A, tick an antidepressant the patient has taken at the minimum dose for at least 6 weeks during THIS episode of depression.
    b. for antidepressants ticked in column A, put another tick in column B if the treatment continued for at least 10 weeks.
    c. tick column C if the patient has taken the drug at a dose equal to or greater than the maximum dosage listed for that medication. *(There is no extra score for doses higher than the maximum)*
    d. If the patient has been prescribed any of the drugs listed here (taken for at least 4 weeks) during the same time period to boost the antidepressant effect, write the name in column D.

For peer review only

i. **NB** – if an antidepressant combination is used, then only score for one of these with the second antidepressant being the augmentation agent. For example if a patient on venlafaxine has mirtazapine added, put a tick in the venlafaxine row and write 'mirtazapine' in Column D. Don't tick the Mirtazapine row (unless this was also used in monotherapy).

ii. **NB** – augmentation agents should in theory be used at minimum effective doses. However, there is a lack of consensus as to what these should be. If in any doubt seek a view of the local Principal Investigators or Chief Investigator.

4. Add the scores:
   a. For each antidepressant add a score to column E. This is +1 for the antidepressant, +0.5 if used for at least 10 weeks (at minimum dose or above), +0.5 if the Maximum dose or greater was used and +0.5 for EACH augmentation agent used.
   b. If the patient has received ECT in this episode, then add 3 into column E on the ECT row at the bottom
   c. Calculate the total MGH TRD score adding up all scores in column E.

For peer review only

Supplementary table 1

Adapted Massachusetts General Hospitals Staging Method to classify treatment resistant depression for the BRIGhTMIND Study.

Has the patient received any treatment with ANTIDEPRESSANT medications since the beginning of THIS CURRENT episode or period of depression?

1 = Yes 2 = No ☐ If NO – Exclude from Study

If Yes

1) On the Grid below in column A, tick an antidepressants the patient has taken at the minimum dose for at least 6 weeks during THIS episode of depression.
2) For antidepressants ticked in column A, put another tick in column B if the treatment continued for at least 10 weeks.
3) Tick column C if the patient has taken the drug at a dose equal to or greater than the maximum dosage listed for that medication. *(There is no extra score for doses higher than the maximum.)*
4) If the patient has been prescribed any of the drugs listed here during the same time period to boost the antidepressant effect, write the name in column D, *Amisulpride, Aripiprazole, Buspirone, Lithium, Methylphenidate, Modafinil, Olanzapine, Pramipexole, Quetiapine, Risperidone, triiodothyronine (T3), and any second Antidepressant.*

	**If ticked score**		1	0.5		0.5	0.5	
			A	B		C	D	
	**Generic name**	**Min dose (mg/day)**	**At least 6 wks**	**At least 10 wks**	**Max dose (mg/day)**	**Equal or greater**	**Name of drug added to augment this antidepressant**	**Score**
**Tricyclic antidepressants**	Doxepin	150			250			
	Clomipramine	150			250			
	Amitriptyline	150			250			
	Nortriptyline	75			125			
	Trimipramine	150			250			
	Imipramine	150			250			
	Dosulepin	75			150			
	Lofepramine	140			210			
**MAOIs**	Isocarboxazid	30			60			
	Phenelzine	45			90			
	Tranylcypromine	30			60			
	Moclobemide	300			600			

SSRIs	Fluvoxamine	50			150			
	Paroxetine	20			60			
	Fluoxetine	20			60			
	Sertraline	50			150			
	Citalopram	20			60			
	Escitalopram	10			30			
SNRI	Venlafaxine	125			250			
	Duloxetine	60			100			
Other	Trazodone	300			600			
	Bupropion	300			450			
	Mirtazapine	15			45			
	Reboxetine	8			12			
	Agomelatin	25			50			
	Vortioxetine	10			20			
Did the patient receive electroconvulsive treatment (ECT) during the current episode (Score 3)?								
							Total MGH score	

Appendix 2 Studies on Cognition, depression and effective connectivity

Background

Depression is associated with marked impairments in attention and executive function (Austin et al., 2001; McDermott and Ebmeier, 2009). Part of these impairments could be explained by increased intraindividual variability (IIV) in attentional performance – patients with depression show a skewed distribution of response times on sustained attention tasks, with an increased frequency of slow responses consistent with attentional lapses (Gallagher et al., 2015). This can be analysed by decomposing the distribution of response times into a Gaussian component and an exponential component (Schmiedek et al., 2007) – Gallagher et al. (2015) found the time constant of the exponential component to be a more sensitive measure for differentiating patients from controls than simple mean reaction times.

It may be that attentional lapses reflect interference from brain areas involved in internally directed mental activity and rumination (the so-called "default mode network" of areas, DMN) on areas involved in executive function (the "frontoparietal network" of areas, FPN). Previous work has found that executive function in non-clinical populations is related to the degree of anticorrelation between the activities of the FPN and DMN (Kelly et al., 2008; Keller et al., 2015), suggesting that the ability to separate the activities of these networks may be critical to task performance. One of the key findings of a meta-analysis of resting-state fMRI studies in patients with depression was an increase in connectivity between the FPN and the DMN, suggesting a breakdown of this separability (Kaiser et al., 2015).

It is unclear the extent to which attentional and executive impairments are independent components of the pathophysiology of depression or a secondary consequence of impairments in mood (Bora et al., 2013; Rock et al., 2014). Independence could be demonstrated in a dissociation between improvements in mood and improvements in cognition during treatment. Cheng et al. (2016) provide preliminary evidence for such a dissociation with a selective improvement in executive function in a group receiving intermittent TBS compared to those receiving continuous TBS or a combination of intermittent and continuous TBS.

Methods

Embedded within the BRIGhTMIND study, we will explore correlations between IIV on the THINC-it battery of cognitive tests (measured with the method of Gallagher et al., 2015) and resting-state functional connectivity between the FPN and the DMN. Separate correlations using baseline measures of each variable and using change in each variable from baseline to 16 weeks will be calculated. Analyses will be performed without unblinding TMS treatment allocation group. We predict a positive correlation between IIV and FPN-DMN functional connectivity at baseline, and a positive correlation between change in IIV and change in FPN-DMN connectivity. We will explore the mediating effects of low mood (measured with the QIDS-SR, Rush et al., 2013), and improvements in mood, on these relationships. Additionally, we will examine the longitudinal relationship between mood and IIV in a within-subject design using unblinded QIDS-SR and THINC-it data, to ascertain whether increased IIV is a state or trait property of depression.

For peer review only

References

Austin MP, Mitchell P, Goodwin GM. Cognitive deficits in depression: possible implications for functional neuropathology. *Br J Psychiatry* 2001; **178**:200-6.

Bora E, Harrison BJ, Yücel M, et al. Neurocognitive intra-individual variability in mood disorders: effects on attentional response time distributions. *Psychol Med* 2015; **45**: 2985-97.

Brown S, Rittenbach K, Cheung S, McKean G, MacMaster FP, Clement F. Current and Common Definitions of Treatment-Resistant Depression: Findings from a Systematic Review and Qualitative Interviews. *Can. J. Psychiatry* 2019; **64**: 380–387. https://doi.org/10.1177/0706743719828965

Cheng C-M, Juan C-H, Chen M-H, et al. Different forms of prefrontal theta burst stimulation for executive function of medication-resistant depression: evidence from a randomized sham-controlled study. *Prog Neuro-Psychopharmacol Biol Psychiatry* 2016; **66**: 35-40.

Cleare A, Pariante C, Young A, et al. Evidenced-based guidelines for treating depressive disorders with antidepressants: A revision of the 2008 British Association Psychopharmacology guidelines. J Psychopharmacol 2015;29:459-525.

Fava M. Diagnosis and definition of treatment-resistant depression. *Biol Psychiatry* 2003;**53**:649–59. doi:10.1016/S0006-3223(03)00231-2.

Gallagher P, Nilsson J, Finkelmeyer A et al. Neurocognitive intra-individual variability in mood disorders: effects on attentional response time distributions. *Psychol Med.* 2015;**45**:2985-97. doi: 10.1017/S0033291715000926.

Goss AJ, Kaser M, Costafreda SG, Sahakian BJ, Fu CH. Modafinil augmentation therapy in unipolar and bipolar depression: a systematic review and meta-analysis of randomized controlled trials. *J Clin Psychiatry* 2013; **74**:1101-7. doi: 10.4088/JCP.13r08560.

Kaiser RH, Andrews-Hanna JR, Wager TD, Pizzagalli DA. Large-Scale network dysfunction in major depressive disorder: a meta-analysis of resting-state functional connectivity. *JAMA Psychiatry.* 2015;**72**:603-11. doi: 10.1001/jamapsychiatry.2015.0071.

Keller JB, Hedden T, Thompson TW, Anteraper SA, Gabrieli JD, Whitfield-Gabrieli S. Resting-state anticorrelations between medial and lateral prefrontal cortex: association with working memory, aging, and individual differences. *Cortex.* 2015;**64**:271-80. doi: 10.1016/j.cortex.2014.12.001

Kelly AM, Uddin LQ, Biswal BB et al. Competition between functional brain networks mediates behavioral variability. *Neuroimage.* 2008;**39**:527-37.

Lee JC, Blumberger DM, Fitzgerald PB, Daskalakis ZJ, Levinson AJ. The role of transcranial magnetic stimulation in treatment-resistant depression: a review. Curr Pharm Des. 2012;18:5846-52.

Lobban F, Dodd AL, Sawczuk AP et al. Assessing Feasibility and Acceptability of Web-Based Enhanced Relapse Prevention for Bipolar Disorder (ERPonline): A Randomized Controlled Trial. J Med Internet Res. 2017 Mar 24;19(3):e85. doi: 10.2196/jmir.7008.

McAllister-Williams RH, Anderson IM, Finkelmeyer A, et al. Antidepressant augmentation with metyrapone for treatment-resistant depression (the ADD study): A double-blind, randomised, placebo-controlled trial. *Lancet Psychiatry* 2016; **3**:117–27. doi:10.1016/S2215-0366(15)00436-8

For peer review only

McDermott LM, Ebmeier KP. A meta-analysis of depression severity and cognitive function. *J Affect Disord.* 2009;**119**:1-8. doi: 10.1016/j.jad.2009.04.022

Rock PL, Roiser JP, Riedel WJ, Blackwell AD. Cognitive impairment in depression: a systematic review and meta-analysis. *Psychol Med.* 2014;**44**:2029-40. doi: 10.1017/S0033291713002535.

Rush AJ, Trivedi MH, Ibrahim HM, et al. The 16-item Quick Inventory of Depressive Symptomatology (QIDS) Clinician Rating (QIDS-C) and Self-Report (QIDS-SR): A psychometric evaluation in patients with chronic major depression. *Biol Psychiatry* 2003; **54**: 573-583. doi: 10.1016/s0006-3223(02)01866.

Schmiedek F, Oberauer K, Wilhelm O, et al. Individual differences in components of reaction time distributions and their relations to working memory and intelligence. *J Exp Psychol General* 2007; **136**: 414-429.

Tundo A, de Filippis R, De Crescenzo F. Pramipexole in the treatment of unipolar and bipolar depression. A systematic review and meta-analysis. *Acta Psychiatr Scand* 2019; **140**:116-125. doi: 10.1111/acps.13055.

For peer review only

STANDARD PROTOCOL ITEMS: RECOMMENDATIONS FOR INTERVENTIONAL TRIALS

SPIRIT 2013 Checklist: Recommended items to address in a clinical trial protocol and related documents*

Section/item	Item No	Description	Addressed on page number
**Administrative information**			
Title	1	Descriptive title identifying the study design, population, interventions, and, if applicable, trial acronym	______1_______
Trial registration	2a	Trial identifier and registry name. If not yet registered, name of intended registry	______2_______
	2b	All items from the World Health Organization Trial Registration Data Set	ISRCTN19674644
Protocol version	3	Date and version identifier	_____18________
Funding	4	Sources and types of financial, material, and other support	_____18_______
Roles and responsibilities	5a	Names, affiliations, and roles of protocol contributors	____1,17,18_____
	5b	Name and contact information for the trial sponsor	_____18________
	5c	Role of study sponsor and funders, if any, in study design; collection, management, analysis, and interpretation of data; writing of the report; and the decision to submit the report for publication, including whether they will have ultimate authority over any of these activities	_____18________
	5d	Composition, roles, and responsibilities of the coordinating centre, steering committee, endpoint adjudication committee, data management team, and other individuals or groups overseeing the trial, if applicable (see Item 21a for data monitoring committee)	_____14________

For peer review only

**Introduction**			
Background and rationale	6a	Description of research question and justification for undertaking the trial, including summary of relevant studies (published and unpublished) examining benefits and harms for each intervention	____3,4________
	6b	Explanation for choice of comparators	____3,4________
Objectives	7	Specific objectives or hypotheses	_____4,7_______
Trial design	8	Description of trial design including type of trial (eg, parallel group, crossover, factorial, single group), allocation ratio, and framework (eg, superiority, equivalence, noninferiority, exploratory)	______5_______
**Methods: Participants, interventions, and outcomes**			
Study setting	9	Description of study settings (eg, community clinic, academic hospital) and list of countries where data will be collected. Reference to where list of study sites can be obtained	______5_______
Eligibility criteria	10	Inclusion and exclusion criteria for participants. If applicable, eligibility criteria for study centres and individuals who will perform the interventions (eg, surgeons, psychotherapists)	______5,6______
Interventions	11a	Interventions for each group with sufficient detail to allow replication, including how and when they will be administered	______6,7______
	11b	Criteria for discontinuing or modifying allocated interventions for a given trial participant (eg, drug dose change in response to harms, participant request, or improving/worsening disease)	_____6,7_______
	11c	Strategies to improve adherence to intervention protocols, and any procedures for monitoring adherence (eg, drug tablet return, laboratory tests)	_____6,7_______
	11d	Relevant concomitant care and interventions that are permitted or prohibited during the trial	______7_______
Outcomes	12	Primary, secondary, and other outcomes, including the specific measurement variable (eg, systolic blood pressure), analysis metric (eg, change from baseline, final value, time to event), method of aggregation (eg, median, proportion), and time point for each outcome. Explanation of the clinical relevance of chosen efficacy and harm outcomes is strongly recommended	______8,9, 11___
Participant timeline	13	Time schedule of enrolment, interventions (including any run-ins and washouts), assessments, and visits for participants. A schematic diagram is highly recommended (see Figure)	______9, Figure 1

For peer review only - http://bmjopen.bmj.com/site/about/guidelines.xhtml

Sample size	14	Estimated number of participants needed to achieve study objectives and how it was determined, including clinical and statistical assumptions supporting any sample size calculations	___13_______
Recruitment	15	Strategies for achieving adequate participant enrolment to reach target sample size	_____8________

Methods: Assignment of interventions (for controlled trials)

Allocation:

Sequence generation	16a	Method of generating the allocation sequence (eg, computer-generated random numbers), and list of any factors for stratification. To reduce predictability of a random sequence, details of any planned restriction (eg, blocking) should be provided in a separate document that is unavailable to those who enrol participants or assign interventions	______10_______
Allocation concealment mechanism	16b	Mechanism of implementing the allocation sequence (eg, central telephone; sequentially numbered, opaque, sealed envelopes), describing any steps to conceal the sequence until interventions are assigned	______10_______
Implementation	16c	Who will generate the allocation sequence, who will enrol participants, and who will assign participants to interventions	______10_______
Blinding (masking)	17a	Who will be blinded after assignment to interventions (eg, trial participants, care providers, outcome assessors, data analysts), and how	______13_______
	17b	If blinded, circumstances under which unblinding is permissible, and procedure for revealing a participant's allocated intervention during the trial	______13_______

Methods: Data collection, management, and analysis

Data collection methods	18a	Plans for assessment and collection of outcome, baseline, and other trial data, including any related processes to promote data quality (eg, duplicate measurements, training of assessors) and a description of study instruments (eg, questionnaires, laboratory tests) along with their reliability and validity, if known. Reference to where data collection forms can be found, if not in the protocol	____8-10______
	18b	Plans to promote participant retention and complete follow-up, including list of any outcome data to be collected for participants who discontinue or deviate from intervention protocols	_______14_____

Data management	19	Plans for data entry, coding, security, and storage, including any related processes to promote data quality (eg, double data entry; range checks for data values). Reference to where details of data management procedures can be found, if not in the protocol	Statistical Analysis Plan___________
Statistical methods	20a	Statistical methods for analysing primary and secondary outcomes. Reference to where other details of the statistical analysis plan can be found, if not in the protocol	__10-12_______
	20b	Methods for any additional analyses (eg, subgroup and adjusted analyses)	___11-12_______
	20c	Definition of analysis population relating to protocol non-adherence (eg, as randomised analysis), and any statistical methods to handle missing data (eg, multiple imputation)	___10-12_______
**Methods: Monitoring**			
Data monitoring	21a	Composition of data monitoring committee (DMC); summary of its role and reporting structure; statement of whether it is independent from the sponsor and competing interests; and reference to where further details about its charter can be found, if not in the protocol. Alternatively, an explanation of why a DMC is not needed	_____14,17_____
	21b	Description of any interim analyses and stopping guidelines, including who will have access to these interim results and make the final decision to terminate the trial	_____None______
Harms	22	Plans for collecting, assessing, reporting, and managing solicited and spontaneously reported adverse events and other unintended effects of trial interventions or trial conduct	_____14________
Auditing	23	Frequency and procedures for auditing trial conduct, if any, and whether the process will be independent from investigators and the sponsor	___By sponsor__
**Ethics and dissemination**			
Research ethics approval	24	Plans for seeking research ethics committee/institutional review board (REC/IRB) approval	______14_______
Protocol amendments	25	Plans for communicating important protocol modifications (eg, changes to eligibility criteria, outcomes, analyses) to relevant parties (eg, investigators, REC/IRBs, trial participants, trial registries, journals, regulators)	___No changes_

For peer review only - http://bmjopen.bmj.com/site/about/guidelines.xhtml

Consent or assent	26a	Who will obtain informed consent or assent from potential trial participants or authorised surrogates, and how (see Item 32)	_______8_______
	26b	Additional consent provisions for collection and use of participant data and biological specimens in ancillary studies, if applicable	_____N/A_________
Confidentiality	27	How personal information about potential and enrolled participants will be collected, shared, and maintained in order to protect confidentiality before, during, and after the trial	___Under GCP and Data Protection Act___
Declaration of interests	28	Financial and other competing interests for principal investigators for the overall trial and each study site	_____19__________
Access to data	29	Statement of who will have access to the final trial dataset, and disclosure of contractual agreements that limit such access for investigators	_Statistical Analysis Plan_
Ancillary and post-trial care	30	Provisions, if any, for ancillary and post-trial care, and for compensation to those who suffer harm from trial participation	___None_________
Dissemination policy	31a	Plans for investigators and sponsor to communicate trial results to participants, healthcare professionals, the public, and other relevant groups (eg, via publication, reporting in results databases, or other data sharing arrangements), including any publication restrictions	_____14__________
	31b	Authorship eligibility guidelines and any intended use of professional writers	___17,18________
	31c	Plans, if any, for granting public access to the full protocol, participant-level dataset, and statistical code	Publication of protocol paper and Statistical Analysis Plan
**Appendices**			
Informed consent materials	32	Model consent form and other related documentation given to participants and authorised surrogates	From sponsor
Biological specimens	33	Plans for collection, laboratory evaluation, and storage of biological specimens for genetic or molecular analysis in the current trial and for future use in ancillary studies, if applicable	______N/A_______

For peer review only - http://bmjopen.bmj.com/site/about/guidelines.xhtml

*It is strongly recommended that this checklist be read in conjunction with the SPIRIT 2013 Explanation & Elaboration for important clarification on the items. Amendments to the protocol should be tracked and dated. The SPIRIT checklist is copyrighted by the SPIRIT Group under the Creative Commons "Attribution-NonCommercial-NoDerivs 3.0 Unported" license.

For peer review only

BMJ Open

Connectivity guided theta burst transcranial magnetic stimulation versus repetitive transcranial magnetic stimulation for treatment resistant moderate to severe depression: study protocol for a randomised double-blind controlled trial (BRIGhTMIND).

Journal:	*BMJ Open*
Manuscript ID	bmjopen-2020-038430.R2
Article Type:	Protocol
Date Submitted by the Author:	21-May-2020

Complete List of Authors:	Morriss, Richard; University of Nottingham, Psychiatry<br>Webster, Lucy; Nottinghamshire Healthcare NHS Foundation Trust<br>Abdelghani, Mohamed; Camden and Islington NHS Foundation Trust<br>Auer, Dorothee; University of Nottingham, Arthritis Research UK Pain Centre; University of Nottingham, Sir Peter Mansfield Imaging Centre<br>Barber, Shaun; University of Leicester<br>Bates, Peter<br>Blamire, Andrew; University of Newcastle upon Tyne<br>Briley, Paul; University of Nottingham<br>Brookes, Cassandra; University of Leicester, Leicester Clinical Trials Unit<br>Iwabuchi, Sarina; University of Nottingham<br>James, Marilyn; University of Nottingham, School of Medicine<br>Kaylor-Hughes, Catherine; University of Nottingham<br>Lankappa, Sudheer; Nottinghamshire Healthcare NHS Foundation Trust<br>Liddle, Peter; University of Nottingham<br>McAllister-Williams, Hamish; University of Newcastle upon Tyne<br>O'Neill-Kerr, Alex; Northamptonshire Healthcare NHS Foundation Trust<br>Pszczolkowski, Stefan; University of Nottingham, Precision Imaging Beacon<br>Suazo Di Paola, Ana; University of Leicester, Leicester Clinical Trials Unit<br>Thomson, Louise; University of Nottingham, Psychiatry<br>Walters, Yvette; University of Leicester, Leicester Clinical Trials Unit

**Primary Subject Heading**:	Mental health
Secondary Subject Heading:	Radiology and imaging, Health economics
Keywords:	Depression & mood disorders < PSYCHIATRY, Magnetic resonance imaging < RADIOLOGY & IMAGING, Health economics < HEALTH SERVICES ADMINISTRATION & MANAGEMENT, Adult psychiatry < PSYCHIATRY, Clinical trials < THERAPEUTICS

For peer review only - http://bmjopen.bmj.com/site/about/guidelines.xhtml

I, the Submitting Author has the right to grant and does grant on behalf of all authors of the Work (as defined in the below author licence), an exclusive licence and/or a non-exclusive licence for contributions from authors who are: i) UK Crown employees; ii) where BMJ has agreed a CC-BY licence shall apply, and/or iii) in accordance with the terms applicable for US Federal Government officers or employees acting as part of their official duties; on a worldwide, perpetual, irrevocable, royalty-free basis to BMJ Publishing Group Ltd ("BMJ") its licensees and where the relevant Journal is co-owned by BMJ to the co-owners of the Journal, to publish the Work in this journal and any other BMJ products and to exploit all rights, as set out in our licence.

The Submitting Author accepts and understands that any supply made under these terms is made by BMJ to the Submitting Author unless you are acting as an employee on behalf of your employer or a postgraduate student of an affiliated institution which is paying any applicable article publishing charge ("APC") for Open Access articles. Where the Submitting Author wishes to make the Work available on an Open Access basis (and intends to pay the relevant APC), the terms of reuse of such Open Access shall be governed by a Creative Commons licence – details of these licences and which Creative Commons licence will apply to this Work are set out in our licence referred to above.

Other than as permitted in any relevant BMJ Author's Self Archiving Policies, I confirm this Work has not been accepted for publication elsewhere, is not being considered for publication elsewhere and does not duplicate material already published. I confirm all authors consent to publication of this Work and authorise the granting of this licence.

For peer review only

Connectivity guided theta burst transcranial magnetic stimulation versus repetitive transcranial magnetic stimulation for treatment resistant moderate to severe depression: study protocol for a randomised double-blind controlled trial (BRIGhTMIND).

Richard Morriss, Professor of Psychiatry, University of Nottingham
Lucy Webster, Research Assistant, Nottinghamshire Healthcare NHS Foundation Trust
Mohamed Abdelghani, Consultant Psychiatrist, Camden and Islington NHS Foundation Trust,
Dorothee Auer, Professor of Neuroimaging, University of Nottingham,
Shaun Barber, medical statistician, University of Leicester
Peter Bates, PPI co-ordinator, University of Nottingham
Andrew Blamire, Professor of Neuroimaging, University of Newcastle
Paul M. Briley, Academic Clinical Fellow, University of Nottingham
Cassandra Brookes, Principal medical statistician, University of Leicester
Sarina Iwabuchi, Research Fellow, University of Nottingham
Marilyn James, Professor of Health Economics, University of Nottingham
Catherine Kaylor-Hughes, Research Fellow, University of Nottingham
Sudheer Lankappa, Consultant Psychiatrist, Nottinghamshire Healthcare NHS Foundation Trust
Peter Liddle, Professor of Psychiatry, University of Nottingham
R. Hamish Mc-Allister-Williams, Professor of Affective Disorders, Newcastle University and Honorary Consultant Psychiatrist, Cumbria, Northumberland, Tyne and Wear NHS Foundation Trust
Alex O'Neill-Kerr, Consultant Psychiatrist, University of Northampton
Stefan Pszczolkowski Parraguez, Rresearch Fellow, University of Nottingham
Ana Suazo Di Paola, Medical Statistician, University of Leicester
Louise Thomson, Senior Research Fellow, University of Nottingham
Yvette Walters, trial manager, University of Leicester
BRIGhTMIND study team- Lorraine Bastick, Rosie Carr, Alison Cartlidge, Harry Clark, William Cottam, Robert De Vai, Linda Davison, John Gledhill, Adele Gregory, Christopher Griffiths, Andrew Hamilton, Delilah Harding, Kelly Heath, Rachel Hobson, Gbeminiyi Ireoluwa, Najat Khalifa, Kate Johnstone, Charlotte Kirkland, Mark Liddle, Jessica Lynch, Neil Nixon, Jehill Parikh, Isabel Reid, Noemi Reiner, Sandra Simpson, Beverley Smith, Tina Sore, Joseph Stone, Carly Taylorson, Rebecca Toney, Claire Turner, Sarah Wilkinson, Andy Willis, Tom Willis

Corresponding author: Professor Richard Morriss, Institute of mental Health, University of Nottingham, Triumph Road, Nottingham, NG7 2TU. E-mail: richard.morriss@nottingham.ac.uk

For peer review only

Abstract

Introduction The BRIGhTMIND study aims to determine the clinical effectiveness, cost effectiveness and mechanism of action of connectivity guided intermittent Theta Burst Stimulation (cgiTBS) versus standard repetitive Transcranial Magnetic Stimulation (rTMS) in adults with moderate to severe treatment resistant depression.

Methods and analysis The study is a randomised double-blind controlled trial with 1:1 allocation to either 20 sessions of (a) cgiTBS or (b) neuronavigated rTMS not using connectivity guidance. A total of 368 eligible participants with a diagnosis of current unipolar major depressive disorder that is both treatment resistant (defined as scoring 2 or more on the Massachusetts General Hospital (MGH) Staging Score) and moderate to severe (scoring $\geq$16 on the 17-item Hamilton Depression Rating Scale (HDRS-17)), will be recruited from primary and secondary care settings at four treatment centres in the United Kingdom. The primary outcome is depression response at 16 weeks (50% or greater reduction in HDRS-17 score from baseline). Secondary outcomes include assessments of self-rated depression, anxiety, psychosocial functioning, cognition and quality of life at 8, 16 and 26 weeks post randomisation. Cost effectiveness, patient acceptability, safety, mechanism of action and predictors of response will also be examined.

Ethics and dissemination Ethical approval was granted by East Midlands Leicester Central Research Ethics Committee (ref: 18/EM/0232) on 30th August 2018. The results of the study will be published in relevant peer-reviewed journals, and then through professional and public conferences and media. Further publications will explore patient experience, moderators and mediators of outcome and mechanism of action.

Trial registration number ISRCTN19674644; Pre-results.

252 words

Strengths and Limitations of the study

- Large multicentre randomised controlled trial of cgiTBS versus standard rTMS.
- Carefully characterised population of patients with treatment resistant moderate to severe unipolar major depression with broad inclusion and exclusion criteria to enhance generalisability to clinical care.

For peer review only - http://bmjopen.bmj.com/site/about/guidelines.xhtml

- Explores efficacy, sustainability of treatment response, acceptability, cost effectiveness cognitive function, mechanism of action using MRI and spectroscopy, and clinical and MRI predictors of response in the same sample.
- Unable to completely blind sound and sensation of the treatments being tested.
- Both nature of magnetic stimulation (TBS vs rTMS) and stimulation site identification vary between treatment arms.

Introduction

The lifetime prevalence of major depressive disorder (MDD) is approximately 13% of the general population,[1] and it is the most disabling health condition in terms of years lived with disability.[2] Antidepressants and psychotherapies are effective in treating MDD. However, at least 33% of patients in specialist care,[3] and 22% in general primary care,[4] fail to respond to adequate trials of at least two antidepressants. Therefore, it is essential to find alternative treatments for such "treatment-resistant depression" (TRD).

Neuromodulation techniques that attempt to directly modulate the function of targeted brain regions, such as electroconvulsive therapy (ECT),[5] and repetitive transcranial magnetic stimulation (rTMS),[6] can be effective treatments for MDD. rTMS uses powerful magnetic pulses that can be focussed usually on the left dorsolateral prefrontal cortex (DLPFC) to produce changes in brain activity if it is given as a course of treatments. Unlike ECT, rTMS does not require anaesthesia nor produce potential cognitive deficits, and is cost effective in TRD compared with current standard care.[7]

A recent meta-analysis in TRD demonstrated the acute effectiveness of rTMS when compared with sham TMS, showing significant reductions in depression symptoms, and increases in response and remission and response rates, after treatment. [6] However, the beneficial effects of rTMS on mood in TRD may be relatively short, lasting only 1-3 months. [6,8]

Theta burst stimulation (TBS) is an alternative patterned form of TMS, which employs high frequency stimulation, with each treatment administration requiring less time. Unlike rTMS, TBS uses bursts of magnetic pulses that mimic endogenous theta rhythms and it is associated with cortical long-term potentiation that may induce plasticity in more distal brain areas, such as the hippocampus, potentially leading to a higher proportion of responders and a longer duration of effect in TRD.[9] A meta-analysis of five randomised controlled trials (RCTs) in 221 patients with MDD,[10] found unilateral intermittent TBS (iTBS) applied to the left DLPFC and consecutive iTBS applied to the left DLPFC and then continuous TBS applied to the right DLPFC to be robustly effective versus sham TBS

For peer review only - http://bmjopen.bmj.com/site/about/guidelines.xhtml

on depression symptoms and response. One RCT demonstrating 83% of TRD patients given unilateral iTBS maintained their response at 14 weeks unlike sham and bilateral TBS.[11]. A large RCT of 414 TRD patients comparing iTBS versus rTMS to the left dorsolateral prefrontal cortex (DLPFC) showed a non-inferior reduction in depression symptoms after 4-6 weeks of treatment and at 1, 4 and 12 weeks after treatment.[12] Thus, TBS requires a shorter duration of administration than rTMS without compromising clinical effectiveness. Further studies are required to maximise efficacy and durability of effect of TBS, identify its mechanisms of action and predictors of response.

Brain connectivity changes as detected by task-free, resting state functional magnetic resonance imaging (rsfMRI) may individualise neurostimulation therapy of MDD,[13] with the insula suggested as a target for neuromodulation.[14] In depression, an altered network communication exists within and between affective, cognitive control and default mode networks,[15-18] with a disruption of the reciprocal loop between the DLPFC and insula extending to the sensory regions.[16] In addition, hypometabolism at the right anterior insula predicts remission with cognitive behaviour therapy and similarly, hypermetabolism at the right anterior insula predicts remission with antidepressant treatment in patients with MDD.[19] One biotype of MDD, characterised by strong connectivity between the insula and other regions of the brain, was related to partial treatment response to rTMS of at least 25% improvement on depression symptoms in 82.5% participants.[20]

Therefore, in a small pilot study of 27 TRD patients,[21] individualised left DLPFC targets were determined using Granger causality analysis (GCA) to provide a measure of effective functional connectivity (eFC) seeded from the right anterior insula (rAI). Of the 18 participants who completed all follow-up in the two treatment arms (connectivity guided rTMS (cgrTMS) and connectivity guided iTBS (cgiTBS)) higher response rates were found at three-month follow-up favouring cgiTBS (89%) over cgrTMS (44%), indicating the potential for longer lasting efficacy of cgiTBS.

In addition, rsfMRI and magnetic resonance spectroscopy (MRS) may advance neuromodulation therapy through mechanistic evaluation of effects and response predictions. iTBS may dampen fronto-insular eFC in responders with TRD.[21] Left DLPFC GABA levels are increased significantly in rTMS responders versus non-responders.[22] In addition, DLPFC targeted TMS normalises dysfunctional fronto-limbic networks in TRD, with decreased hyperconnectivity between the subgenual anterior cingulate cortex, ventromedial and dorsomedial prefrontal cortex and DLPFC.[23]

iTBS may induce widespread and longer-term network change but precise anatomical localisation of the target circuitry may be required for maximal efficacy of iTBS to normalise dysfunctional fronto-limbic circuitry. Such changes may increase the proportion of people with TRD who obtain a

For peer review only - http://bmjopen.bmj.com/site/about/guidelines.xhtml

sustained response compared to neuronavigated rTMS. Thus, this multi-centre RCT will examine the clinical and cost effectiveness of cgiTBS in comparison with neuronavigated rTMS, in treatment resistant moderate to severe MDD.

Methods and analysis

Study Design

The study is a multicentre parallel group, double blind, randomised, controlled trial of the efficacy of cgiTBS versus neuronavigated rTMS without connectivity guidance, in patients with a primary diagnosis of moderate to severe MDD, which is treatment resistant in their current episode (TRD). The study will be carried out at four sites across UK National Health Services (NHS): Nottinghamshire Healthcare Foundation NHS Trust, Northamptonshire Healthcare NHS Foundation Trust, Cumbria, Northumberland, Tyne and Wear NHS Foundation Trust and Camden and Islington NHS Foundation Trust. The study flow chart (Figure 1) provides an overview of the study design and procedures.

[Insert Figure 1]

Study population and recruitment

368 participants will be recruited in total (184 per treatment arm). Recruitment of the first patient started 22nd January 2019 but was suspended on 23rd April 2020 because of the Covid-19 pandemic lockdown. The study is due to complete on 30th September 2021. Table 1 shows the detailed inclusion/exclusion criteria. Participant identification will occur through primary and secondary care NHS settings across the four sites.

Table 1. Inclusion and Exclusion Criteria

Inclusion Criteria	Exclusion Criteria
• Adults $\geq$ 18 years<br>• Diagnosis of current MDD (defined according to the Diagnostic and Statistical Manual of Mental Disorders, Fifth Edition), that is treatment resistant defined as scoring 2 or	• History of bipolar disorder (due to risk of mania) or depression secondary to other mental disorder.<br>• Neurological conditions e.g. brain neoplasm, cerebrovascular events, epilepsy,

For peer review only - http://bmjopen.bmj.com/site/about/guidelines.xhtml

more on the Massachusetts General Hospital Treatment Resistant Depression staging score (appendix 1).<br>• Have a 17-Item Hamilton Depression Rating Scale score of 16 or more (moderate to severe depression).<br>• Capacity to provide informed consent before any trial related activities.	neurodegenerative disorders, and prior brain surgery.<br>• Standard contraindications to MRI i.e. irremovable metal objects in and around body e.g. cardiac pacemaker, implanted medication pump, and pregnancy (any doubt resolved by pregnancy test, women of childbearing age taking precautions against pregnancy) This will include other potential complicated factors such as red tattoos which consist of iron on the head, neck and back and claustrophobia (we offer mock scanner testing and training in some sites).<br>• Major unstable medical illness requiring further investigation or treatment.<br>• Change in prescribed medication 2 weeks before baseline assessment.<br>• Prescription of lamotrigine, gabapentin, pregabalin in the 2 weeks prior to baseline assessment but may be used before then.<br>• Daily prescription of benzodiazepine above 5 mg diazepam equivalents, Zopiclone above 7.5mg, zolpidem above 10mg or Zaleplon above 10mg. These drugs should not be used intermittently in the 2 weeks before baseline assessment but may be used before then.<br>• Current substance abuse or dependence (defined by DSM-5 criteria).<br>• Prior TMS treatment.<br>• At risk of suicidality.<br>• Potential complicated factors relating to the TMS treatment i.e. Hairstyles which would impair magnetic transmission and piercings. (Participants would only be excluded if they chose to not make the changes required to ensure effective treatment.)<br>• Involved with any other clinical trial at the

For peer review only

time of consent or 6 months prior.

- Unable to read or understand English.

Interventions

For all treatments, a 70mm figure-of-eight coil (E-z Cool coil) and a Magstim® Horizon® Performance Stimulator with StimGuide® Navigated TMS Package (Magstim Company, Whitland, United Kingdom) will be used. Participants in both treatment arms will receive neuronavigation guided single coil stimulation for 20 TMS sessions delivered over 4-6 weeks. A total of 3000 pulses will be delivered in each rTMS or cgiTBS session. Each treatment session, irrespective of treatment arm, is designed to last approximately 37.5 minutes in total for the purposes of blinding the participants and assessors of outcome. There must not be a gap of more than 4 days between treatments in the course of 20. Medication will be unchanged over 16 weeks follow up unless there is clinical risk of harm.

Individuals assigned to cgiTBS will receive bursts of 3 pulses (80% motor threshold) at 50Hz applied at a frequency of 5 Hz (i.e. every 200 ms) for 40 seconds duration over a site determined from the assessment of maximal strength of effective connectivity between the right anterior insula and the left DLPFC from functional magnetic resonance imaging (fMRI) and structural magnetic resonance imaging (MRI). Neuronavigation computes the nearest location for TBS stimulus on the scalp from an individualised head model based on structural MRI and three fiducial points, the nasion, left preauricular and right preauricular sites. If this site causes discomfort to the participant, it is moved by 1 cm in any direction until there is no discomfort. The pulses are repeated for a total of 5 runs with 5-minute rest intervals between runs.

Individuals assigned to rTMS will follow the standard US Food and Drug Administration (FDA) approved protocol. The site of stimulation will be determined using structural and fMRI utilising neuronavigation to compute the F3 electrode site over the left DLPFC for TMS stimulation from the same head model and three fiducial points. Stimulation is at 120% motor threshold with 75 x 4-second trains of 10Hz interspersed by 26-second intertrain intervals.

Primary Clinical Objective

Our primary hypothesis is that cgiTBS is more efficacious at 16 weeks than standard rTMS in patients with TRD as assessed by the proportion of patients who show a response (50% reduction in depression symptoms from baseline on the HDRS-17).

For peer review only - http://bmjopen.bmj.com/site/about/guidelines.xhtml

Secondary Clinical Objectives

- To explore secondary efficacy outcomes of the mean change in HDRS-17 scores between the two treatment arms and the proportion of patients meeting criteria for remission (HDRS-17 score ≤7) at 16 weeks.
- To explore secondary clinical outcomes of importance to patients and clinicians namely cognition, anxiety, social function and quality of life.
- To examine cost effectiveness of cgiTBS versus rTMS in a UK NHS population.
- To examine the patient acceptability and patient experience of cgiTBS and rTMS.

Mechanistic Objectives

- To investigate the neural mechanism of efficacy in cgiTBS and rTMS.
- To develop response prediction models from brain biotypes and clinical features.

Protocols for the study will be published separately describing the mechanistic objectives and localisation of sites of stimulation in greater detail.

Study Schedule

Trained researchers under the supervision of clinically trained investigators will obtain informed consent and undertake all assessments. A pre-screening questionnaire will be used to telephone screen interested participants first, with potentially eligible participants invited to attend a baseline assessment. Written informed consent will be obtained and study eligibility determined at the baseline assessment.

Information on socio-demographics, past medical and psychiatric history including a detailed assessment of treatment resistance will be obtained from case files and primary care notes. Diagnosis will be assessed by the Structured Clinical Interview (SCID-5)[24] for the Diagnostic and Statistical Manual of Mental Disorders, Fifth Edition (DSM-5).[25]

Treatment resistance will be measured by the Massachusetts General Hospital (MGH) TRD staging score (adapted for new treatment options - see supplementary material, appendix 1 and table 1).[26]

Depression severity and the primary outcome measure will be assessed using the GRID version of HDRS-17.[27] The HDRS-17 and secondary outcome measures will be assessed in eligible patients at baseline, 8, 16 and 26 weeks post randomisation (Table 2). The primary outcome measure is a binary variable of responder or non-responder at 16 weeks. Individuals observed to have a 50% drop or

For peer review only - http://bmjopen.bmj.com/site/about/guidelines.xhtml

greater in HDRS-17 from baseline to 16 weeks are defined as responders. In contrast, individuals with <50% reduction or a null value are classified as non-responders. The Childhood Trauma Questionnaire (CTQ)[28] will be assessed at baseline as a moderator of treatment response at 16 weeks.

Secondary outcome measures are the Beck Depression Inventory-II (BDI-II),[29] Patient Health Questionnaire (PHQ-9),[30] Generalised Anxiety Disorder Assessment (GAD-7),[31] Work and Social Adjustment Scale (WSAS),[32] EuroQol-5D-5L,[33] and cognitive functioning as measured by the THINC-Integrated Tool (THINC-it) (THINC-it Task Force, http://thinc.progress.im/en). Quick Inventory of Depressive Symptomatology (QIDS-SR) [34] is completed for a mechanism study (see supplementary material – Appendix 2).

In addition, a purposely designed patient proforma will be used to collect patient NHS service utilisation information at the 16 and 26 week follow up time points. This will cover relevant items listed in the Client Service Receipt Inventory (CSRI),[35] and tailor the resources items measured following good practice approaches used by the health economics DiRUM (database of instruments for resource use measurement) group to estimate costs. Patient acceptability will be measured on a 1-5 scale from unacceptable to acceptable and patient experience of overall improvement will be assessed using the Patient Global Impression of Change (1-5 scale much worse to much better)[36] after each TMS session and at each follow up time point. A side effect checklist will be completed after each TMS session and 8 weeks follow up assessment. We will note any changes in medication or other forms of treatment.

Table 2. Study Schedule and Assessments

**Outcome measures**	**Baseline Assessment (Consent to the study)**	**Baseline MRI Scan**	**Treatment Mon-Fri for 4-6 weeks**	**8 Week Follow up Assessment**	**16 Week Follow Up Assessment**	**16 Week MRI Scan**	**26 Week Follow Up Assessment**
Visit Window		Within 14 days of Baseline Assessment	+ 14 days of MRI Scan	+/- 1 week from Randomisation	+/- 1 week from Randomisation	Within 14 days of 16 Week Follow Up Assessment	+/- 1 week from Randomisation
HDRS-17	✓		✓Only if Baseline assessment exceeds 4 weeks	✓	✓		✓
MGH	✓		✓ Only if Baseline assessment exceeds 4 weeks				
BDI-II	✓			✓	✓		✓
PHQ-9	✓			✓	✓		✓
WSAS	✓			✓	✓		✓
GAD7	✓			✓	✓		✓
EQ-5D-5L	✓			✓	✓		✓
THINC-it	✓			✓	✓		✓

For peer review only - http://bmjopen.bmj.com/site/about/guidelines.xhtml

SCID-5	✓						
CTQ	✓						
QIDS-SR	✓			✓	✓		✓
Client Resource Questionnaire	✓				✓		✓
Patient Acceptability			✓	✓	✓		✓
Side Effects Checklist (Adverse Events)			✓	✓			
fMRI		✓				✓	
MRI		✓				✓	
rsfMRI)		✓				✓	
MRS		✓				✓	
Diffusion weighted imaging		✓				✓	

Participants will undergo baseline structural MRI, resting state fMRI, diffusion weighted imaging scans to assess structural connections between relevant brain regions, and MRS scans (not in London where only fMRI and structural MRI scans will be performed). Treatment target identification will be analysed centrally in Nottingham and communicated to treatment sites. Using GCA of fMRI scans in each subject, coordinates for the stimulation target within the left DLPFC that shows maximal effective connectivity with the right anterior insula will be identified. For the MRS scans, voxels will be placed in the DLPFC and anterior cingulate cortex (ACC), using a similar method to prior work with the MEGA-PRESS sequence for GABA-edited MRS. [22] The MRI scans are repeated at 16 weeks to assess functional connectivity, effective connectivity and GABA.

Randomisation

Randomisation will take place immediately prior to the start of the first treatment session, with participants randomised to either rTMS or cgiTBS treatments. Randomisation will be conducted via a web-based randomisation system (Sealed Envelope, www.sealedevelope.com) by a named TMS nurse practitioner and health care assistant delivering TMS at each centre who will remain un-blinded. Participants will be randomly assigned in a 1:1 ratio into the rTMS and cgiTBS arms using blocks of varying size. Randomisation will be stratified by centre and minimised on severity of depression at baseline and degree of treatment resistance. Baseline depression will be measured by HDRS-17 score and classified as moderate (16-23) or severe (≥24). [37]. Treatment resistance will be measured by the MGH TRD staging score and, based on distributions of treatment resistance in the ADD study,[38], degree of treatment resistance will be classified as low (2-3.5), medium (4-6) and high (≥6.5).

For peer review only

For peer review only - http://bmjopen.bmj.com/site/about/guidelines.xhtml

Statistical Analyses

Efficacy analysis

The primary analysis will test the null hypothesis that treatment with cgiTBS does not increase response rate as measured by HDRS-17 compared to rTMS at 16 weeks on an intent to treat population with any missing response data at 16 weeks being imputed as no response having been achieved. Response at 16 weeks was selected as the primary outcome because of the much larger response rate with cgiTBS than rTMS in our pilot study. [21] A logistic regression will be fitted for the outcome of response with treatment arm, centre, baseline HDRS-17 score and MGH TRD score.

As a secondary analysis a logistic regression model will be repeated in completers (those with 10 or more rTMS or cgiTBS treatments, assessed at baseline and 16 weeks) and a per protocol analysis. Secondary outcomes are the and proportion of responders and remitters at 8, 16 and 26 weeks, and sustained responders at both 16 and 26 weeks will be compared between groups using logistic regression adjusted for treatment centre, baseline HDRS-17 score and MGH TRD score (5% significance). All outcomes are shown in Table 3. Repeated measure linear models will be used for continuous outcomes. Patient acceptability (5-point scale) and safety of both cgiTBS and rTMS (side-effects checklist) will be reported descriptively.

We will explore moderators of depression response at 16 weeks, namely severity of depression by baseline HDRS-17 score, degree of treatment resistance, childhood trauma and age. The number of TMS sessions attended will be explored as a mediator of outcome in exploratory sub-group analyses of the depression response at 16 weeks.

Table 3. Study efficacy outcome measures

Outcomes	Measure	Time Points
Primary Outcome	17-Item Hamilton Depression Rating Scale (HDRS-17)	Response at 16 weeks (defined as by a 50% or greater reduction in HDRS-17 score from baseline)
Secondary Outcomes	HDRS-17	Mean change in HDRS-17 score compared with baseline at 8, 16 and 26 weeks
		Response (as defined above) at 8 weeks and at 26 weeks

For peer review only - http://bmjopen.bmj.com/site/about/guidelines.xhtml

		Sustained response at 16 and 26 weeks (defined as a continuing response as defined above following a response at the previous time point)
		Remitters at 8, 16 and 26 weeks (defined as a score of 7 or less on the HDRS-17)
	Beck Depression Inventory-II (BDI-II)	At 8, 16 and 26 weeks
	Patient Health Questionnaire (PHQ-9)	At 8, 16 and 26 weeks
	Quick Inventory of Depressive Symptomatology (QIDS SR)	At 8, 16 and 26 weeks
	Generalised Anxiety Disorder Assessment (GAD-7)	At 8, 16 and 26 weeks
	THINC Integrated Tool (THINC-it)	At 8, 16 and 26 weeks
	EuroQol-5D-5L	At 8, 16 and 26 weeks
	Work and Social Adjustment Scale (WSAS)	At 8, 16 and 26 weeks
	Patient Acceptability (1-5 scale)	After each TMS session and at 8, 16 and 26 weeks
	Adverse Events Checklist	After each TMS session and 8 weeks
	MRI scan (structural [T1], diffusion weighted imaging [DWI] for structural connectivity, resting state fMRI for functional connectivity [FC] and effective connectivity [eFC], and GABA MRS of the DLPFC and insula	T1, DWI, FC, eFC at baseline (all centres)<br>GABA at baseline and T1, DWI, FC, eFC, GABA at 16 weeks (all sites except London).

For peer review only

Economic analysis

The economics analysis will take an NHS and personal social services cost perspective in accordance with NICE guidance, including medication, inpatient and outpatient hospital visits and primary and community care use. It will also take a wider societal perspective to capture the broader effects of rTMS and cgiTBS on depression such as paid employment, caring responsibilities and other implications for friends and family. Resource data will then form the units on which cost data, using sources such as the Unit Cost of Health and Social Care\PSSRU,[39] the British National Formulary (BNF), and national reference costs can be attached. The nurse and health care assistant at each centre will complete a diary of time spent managing each participant in the RCT to derive treatment

For peer review only - http://bmjopen.bmj.com/site/about/guidelines.xhtml

costs for rTMS and cgiTBS. The number of treatment sessions for each treatment arm will be carefully recorded and a separate intervention cost assigned to each of the therapies. We will delineate the time spent delivering cgiTBS or rTMS from time spent on research only procedures, such as imaging for delivery of rTMS and additional mechanistic scans in both treatment arms or the extended length of the TBS session for blinding, that would not be used in the real clinical world. The outcome measure for the economic evaluation will be the number of quality-adjusted life year (QALYs) based on a six-month time horizon with no discounting for costs or outcomes as they accrue within a 12-month period. An incremental analysis will be used between the two groups and where appropriate an incremental cost effectiveness ratio (ICER) will be reported between rTMS and cgiTBS. We will use the net monetary benefit framework and implement a net benefit regression,[40] to estimate the extent to which, and the probability that, the cgiTBS intervention is cost-effective compared to standard rTMS at a range of threshold values for the willingness to pay per QALY, generating cost effectiveness acceptability curves (CEACs). Data will be analysed for baseline and centre effects. Key cost drivers will be examined using probabilistic sensitivity analysis.

Qualitative analysis

A purposive sample of 25-30 participants from both arms and all centres, reflecting a mix of demographic characteristics, consent or non-consent to participate, adherence and non-adherence to treatment and follow up will be selected for qualitative interviews, each lasting for up to an hour, after the 16-week assessment (the primary outcome). We will ask about their general views of TMS, benefits from, disadvantages from or dislikes about receiving TMS. Interviews will continue until theme saturation is achieved, i.e. no more themes emerge in subsequent interviews. Qualitative interviews of barriers to recruitment, with staff members involved in recruitment, will also be completed to optimise strategies to improve recruitment as the RCT proceeds. All interviews will be recorded and transcribed verbatim. Inductive thematic analysis using a grounded approach will be adopted.

Sample Size

Sample size is calculated on the minimum clinically important difference in responder rates from baseline to 16 weeks of 15% (equivalent to a number needed to treat (NNT) of 7) between two active treatments (cgiTBS versus rTMS) in favour of the experimental condition cgiTBS. This is an effect size regarded in the literature as a clinically important difference in studies using invasive approaches to manage TRD such as vagal nerve stimulation.[40] The most recent meta-analysis of 16

For peer review only - http://bmjopen.bmj.com/site/about/guidelines.xhtml

RCTs in 977 participants with TRD reports a response rate on the HDRS-17 and the Montgomery–Åsberg Depression Rating Scale of 26.5% for rTMS at the end of treatment versus 13.1% for sham TMS.[6] Assuming a response rate on the HDRS-17 of 26.5% with rTMS and 41.5% with cgiTBS, 368 patients in total (184 per arm) will provide a power of 80% to detect the difference in response rates at a 5% significance level (two-tailed), allowing for 15% loss to follow-up at 16 weeks. [6]

Blinding

Patients, referring clinical teams and the outcomes assessors will be kept blind with respect to the treatment protocol assigned and administered. Any unintended unblinding of outcome assessors will be recorded and another assessor will complete all further assessments for that participant. At each assessment, the outcomes assessor will be asked to guess the treatment allocation of the participant.

Ethics and dissemination

Ethical Considerations

Ethical approval was granted by East Midlands Leicester Central Research Ethics Committee (ref: 18/EM/0232) on 30/08/2018. The study was registered on 02/10/2018 (ISRCTN19674644) under the public title “BRIGhTMIND: brain imaging guided transcranial magnetic stimulation in depression”. The first participant was randomised on 22/01/19. Study finish is scheduled for September 2021. Overview of the study is performed by an external independent Data Monitoring Ethics Committee reporting to a Trial Steering Committee.

Expenses will be covered for participation in the study along with a £10.00 shopping voucher at 16 and 26 Week follow up assessments as a mark of respect and gratitude for the time and input of the participants to the follow up aspects of the trial.

Safety Considerations

For peer review only - http://bmjopen.bmj.com/site/about/guidelines.xhtml

Internationally agreed definitions of adverse events (any untoward medical occurrence in a clinical trial subject administered TMS whether or not it has a causal relationship with TMS) and serious adverse events (any adverse event or adverse reaction that results in death, is life-threatening, requires hospitalisation or prolongation of existing hospitalisation, results in persistent or significant disability or incapacity, or is a congenital anomaly or birth defect). All participants will be asked about adverse events after every treatment (immediately and a maximum of 72 hours later) until they are resolved. Any participant found to be at risk to themselves (suicide, neglect) or others, or developing a serious adverse event will be referred to relevant clinical services. A review by a clinical expert in TRD will be offered to any participant whose depression has become more severe at 16 and 26 weeks for safety reasons.

Dissemination

All participants will be sent a report summary of the results. Results of the study will be published in peer-reviewed academic journals and communicated widely to academic, profession and public audiences through conferences, media and social media.

Patient and public involvement.

Regarding this study a co-production approach has been taken. We worked with the Involvement Centre at Nottinghamshire Healthcare Trust to create a Magnetic Stimulation Advisory Group who co-produced the treatment pathway for prior pilot work [21] and the current study.

During the study, each centre will be invited to contribute at least two Patient and Public Involvement (PPI) representatives to form a Lived Experience Advisory Panel (LEAP) who will contribute to all study meetings and a representative will be invited to attend all research meetings. At each site PPI representatives will seek opinions, test ideas and gain support for the study. Participant information sheets and other study materials will be co-designed. They will see oversee the interpretation of our findings, particularly the emerging qualitative analysis on barriers to recruitment and patient acceptability of TMS. The PPI process will be overseen by an experienced PPI lead who has trained supported and mentored many PPI groups over recent years. The voice of experts by experience will be heard in all dissemination activities, including presentations or publications.

For peer review only - http://bmjopen.bmj.com/site/about/guidelines.xhtml

Discussion

Although TMS is an established efficacious and safe treatment approach for TRD, research is required to establish if it is possible to increase the proportion of people with TRD who derive a sustained improvement in depression response. [8] Based on theoretical and preliminary empirical evidence, this study explores the potential of using precise personalised neuroanatomical stimulation at a site where affective, cognitive control and default mode networks overlap in neuroanatomical space as determined by MRI derived maximal strength of connectivity between the right anterior insula and the left DLPFC. It will be compared with standard rTMS in a large multicentre randomised controlled trial. Both rTMS and iTBS are effective against sham treatments [6, 8, 10] and recommended for clinical practice in the UK. [8] Furthermore, standard iTBS is non-inferior but not superior to rTMS. [12] Thus, if the current RCT showed that cgiTBS was superior in efficacy, it would be reasonable to conclude that the increased efficacy was due to the personalisation of the site of iTBS treatment.

The design of the trial aims to have high external validity and generalisability to inform routine clinical care. Therefore, inclusion and exclusion criteria are broad and confined to ensuring that safety, efficacy and the investigation of mechanism of action are not compromised. Our definition of TRD [26] is one of the most widely used but it does not consider psychological treatment and underestimates treatment resistance in those who refuse or are not offered antidepressants or ECT in the current episode because of lack of effectiveness or intolerance in previous depression episodes. The broad inclusion and exclusion criteria, large sample size, inclusion of clinical and MRI variables and long duration of follow up will permit a wide variation in potential predictors and non-predictors of response for clinical practice and further research.

Compromises have been made to the design of the study to improve blinding. cgiTBS and rTMS have been matched procedurally so both use the same MRI and neuronavigation system, although these are not currently being used in clinical practice in UK. TMS treatments have also been matched in terms of pulses received and duration of treatment, although the duration of cgiTBS is delivered over a much longer duration but at a lower motor threshold than the United States Federal Drug Agency protocol used in the Three-D study. [12] We decided to use the motor threshold that was well tolerated in our pilot study. [21] We also did not include a third arm of connectivity guided rTMS because our pilot study showed a short duration of response similar to standard rTMS [21]. The use of sham coils to mask the sound and sensation of iTBS and rTMS was explored but effective

For peer review only - http://bmjopen.bmj.com/site/about/guidelines.xhtml

shams were not available. As a result, entry into the study is restricted to people who had never previously received TMS.

A co-productive approach has been taken with our patient and public LEAP group and this has been particularly important around informing participants accurately about the study and managing participant expectations. By doing this we hope to optimise recruitment and retention to the study.

In many countries, 3 Tesla MRI is increasingly available so that cgiTBS could be implemented into routine care for TRD. Should the RCT demonstrate that cgiTBS is clinically effective at 16 weeks and cost effective compared to rTMS, then the possibility of maintaining participants with one full course of cgiTBS with booster sessions two or three times per year using the same site of stimulation from a baseline MRI might be feasible. If it is not effective, then other parameters of delivering TMS should be the focus of research and development.

Figure legend

Figure 1. Study Flow Chart

Abbreviations

ACC: anterior cingulate cortex; BDI: Beck Depression Inventory; BNF: British National Formulary; CEACs; Cost effectiveness acceptability curves; cgiTBS: Connectivity Guided Intermitted Theta Burst Stimulation; cgrTMS: Connectivity Guided repetitive Transcranial Magnetic Stimulation; CRF: Case Report Forms; CSRI: Client Service Receipt Inventory; cTBS: continuous Theta Burst Stimulation; CTQ: Childhood Trauma Questionnaire; DiRUM: Data of instrument for resource use measurement; DLPFC: Dorsolateral Prefrontal Cortex; DMPFC: Dorsomedial Prefrontal Cortex; DSM-5: The Diagnostic and Statistical Manual of Mental Disorders, Fifth Edition; ECT: Electroconvulsive Therapy; eFC: Effective Functional Connectivity; FC: Functional Connectivity; FDA: Food and Drug Administration; fMRI: Functional Magnetic Resonance Imaging; GABA: Gamma-amino butyric acid; GAD-7: Generalised Anxiety Disorder Assessment; GCA: Granger Causality Analysis; glx: glutamate and glutamine; GRID-HAMD: 17-Item Hamilton Depression Rating Scale GRID form; HDRS-17: 17-Item Hamilton Depression Rating Scale; ICER; Incremental cost effectiveness ratio; iTBS: intermittent Theta Burst Stimulation; MADRS: The Montgomery–Åsberg Depression Rating Scale; MDD: Major Depressive Disorder; MGH: Massachusetts General Hospital; MRI: Magnetic Resonance Imaging; MRS: Magnetic resonance spectroscopy; NHS: National Health Services; NICE: National Institute of Health and Care Excellence; NNT: Number need to treat; PHQ-9: Patient Health Questionnaire; QALY's: Quality-adjusted life year; rAI: right anterior insula; RCT: Randomised Controlled Trials; rsfMRI: resting state Functional magnetic resonance imaging; rTMS: repetitive Transcranial Magnetic Stimulation; SCID-5: Structured Clinical Interview for DSM-5; TBS: Theta Burst Stimulation; THINC-it:

For peer review only - http://bmjopen.bmj.com/site/about/guidelines.xhtml

THINC Integrated Tool; TMS: Transcranial Magnetic Stimulation; TRD: Treatment Resistant Depression; WSAS: Work and Social Adjustment Scale

Acknowledgements

We thank the ongoing support of the Funders, the Patient and Public Involvement representatives, participants, research and treatment staff at the four centres, the NIHR Clinical Research Network in East Midlands, North East and North Thames, the clinical, methodological and lay members of the independent Data Monitoring Ethics Committee and Trial Steering Committees, the Leicester Clinical Trials Unit and all other staff whom are involved in the study. RM is supported by the Nottingham National Institute for Health Research (NIHR) Biomedical Research Centre, NIHR MindTech Med Tech and in Vitro Centre and NIHR Applied Research Centre East Midlands.

Authors' contributions

All authors approved the final manuscript.

Richard Morriss is the chief investigator and guarantor of the project. He designed the study, obtained funding and wrote the final draft of the protocol.

Lucy Webster wrote operational guidelines for the project, qualitative analysis protocols and the first draft on this manuscript.

Mohamed Abdelghani is the principal investigator for the London site, obtained funding, and designed the TMS schedules.

Dorothee Auer is the neuroimaging lead, led some of the underpinning work designed the neuroimaging and obtained grant funding.

Shaun Barber carried out power calculations for the study and designed the statistical analysis

Peter Bates has led the patient and public involvement aspects of the project and their contributions to the operation of the project.

Andrew Blamire designed the neuroimaging and obtained grant funding.

Paul Briley designed some of the neuroimaging protocol and is analysing the imaging data

Cassandra Brookes designed the statistical analysis and obtained funding for the study.

Sarina Iwabuchi led some of the underpinning work, designed the neuroimaging and TMS parts of the protocol and obtained funding.

Marilyn James designed the health economic analysis, the health economic data collection forms and will be responsible for the health economic management of the trial and oversee all the health economic analysis.

Catherine Kaylor-Hughes wrote protocols for TMS in the study and carried out some of the neuroimaging analysis

For peer review only - http://bmjopen.bmj.com/site/about/guidelines.xhtml

Alex Kerr is the principal investigator for the Northampton site, obtained funding, and designed the TMS schedules.

Sudheer Lankappa, the principal investigator for the Nottingham site, carried out some of the underpinning work, obtained funding, and designed the TMS schedules.

Peter Liddle carried out some of the underpinning work and designed some of the neuroimaging and TMS work, and obtained funding.

Hamish Mc-Allister-Williams is the principal investigator for the Newcastle site, designed the cognitive assessment and assessment of treatment resistance and obtained funding

Stefan Pszczolkowski designed and carried out the computer programming for TMS site location, helped design the Neuronavigation and TMS protocols, and is carrying out neuroimaging analysis.

Ana Suazo Di Paola designed the Statistical Analysis Plan and is carrying out the statistical analysis.

Louise Thomson is the qualitative research lead, designed the qualitative parts of the project and obtained funding.

Yvette Walters is the trial manager of the study and has led the writing of study operating procedures, ethics and governance protocols for the study.

Protocol version

Version 4.0 22/01/2020

Funding

This project (project reference 16/44/22) is funded by the Efficacy and Mechanism Evaluation (EME) Programme, an MRC and NIHR partnership. The views expressed in this publication are those of the author(s) and not necessarily those of the MRC, NIHR or the Department of Health and Social Care. Equipment for the study was provided by Magstim Company Ltd

Sponsor

Research and Evidence Team, Nottinghamshire Healthcare NHS Foundation Trust. Contact e-mail: research@nottshc.nhs.uk. Neither the study sponsor nor funder has or will have any role in designing, collecting, analysing or interpreting the data or any study reports.

Competing Interests

Alex Kerr has received fees for consultancy with Magstim. No other competing interests declared.

References

1 Kessler RC, Bromet EJ. The epidemiology of depression across cultures. *Annu Rev Public Health.* 2013;34:119-38. doi: 10.1146/annurev-publhealth-031912-114409.

2 World Health Organisation. *Depression and Other Common Mental Disorders: Global Health*

For peer review only

For peer review only - http://bmjopen.bmj.com/site/about/guidelines.xhtml

Estimates. Geneva: World Health Organization; 2017. Licence: CC BY-NC-SA 3.0

3 Rush AJ, Trivedi MH, Wisniewski SR, *et al.* Acute and longer-term outcomes in depressed outpatients requiring one or several treatment steps: A STAR*D report. *Am J Psychiatry* 2006;**163**:1905–17. doi:10.1176/ajp.2006.163.11.1905

4 Rizvi SJ, Grima E, Tan M, *et al.* Treatment-resistant depression in primary care across Canada. *Can J Psychiatry* 2014;**59**:349–57. doi:10.1177/070674371405900702

5 Khalid N, Atkins M, Tredget J, *et al.* The effectiveness of electroconvulsive therapy in treatment-resistant depression: A naturalistic study. *J ECT* 2008;**24**:141–5. doi:10.1097/YCT.0b013e318157ac58

6 Health Quality Ontario. Repetitive Transcranial Magnetic Stimulation for Treatment-Resistant Depression: A Systematic Review and Meta-Analysis of Randomized Controlled Trials. *Ont Health Technol Assess Ser.* 2016;**16**:1–66.

7 Simpson KN, Welch MJ, Kozel FA, *et al.* Cost-effectiveness of transcranial magnetic stimulation in the treatment of major depression: A health economics analysis. *Adv Ther* 2009;**26**:346–68. doi:10.1007/s12325-009-0013-x

8 NICE:IPG542. *Repetitive transcranial magnetic stimulation for depression*.2015.https://www.nice.org.uk/guidance/ipg542

9 Larson J, Munkácsy E. Theta-burst LTP. Brain Res. 2015;**1621**:38–50. doi:10.1016/j.brainres.2014.10.034

10 Berlim MT, McGirr A, Rodrigues Dos Santos N, *et al.* Efficacy of theta burst stimulation (TBS) for major depression: An exploratory meta-analysis of randomized and sham-controlled trials. *J Psychiatr Res* 2017;**90**:102–9. doi:10.1016/j.jpsychires.2017.02.015

11 Li C-T, Chen M-H, Juan C-H, *et al.* Efficacy of prefrontal theta-burst stimulation in refractory depression: a randomized sham-controlled study. *Brain* 2014;**137**:2088–98. doi:10.1093/brain/awu109

12 Blumberger DM, Vila-Rodriguez F, Thorpe KE, *et al.* Effectiveness of theta burst versus high-frequency repetitive transcranial magnetic stimulation in patients with depression (THREE-D): a randomised non-inferiority trial. *Lancet* 2018;**391**:1683–92. doi:10.1016/S0140-6736(18)30295-2.

13 Fox MD, Liu H, Pascual-Leone A. Identification of reproducible individualized targets for treatment of depression with TMS based on intrinsic connectivity. *Neuroimage.* 2013;**66**:151-60. doi: 10.1016/j.neuroimage.2012.10.082.

14. Iwabuchi SJ, Peng D, Fang Y, *et al.* Alterations in effective connectivity anchored on the insula in major depressive disorder. *Eur Neuropsychopharmacol* 2014;**24**:1784–92. doi:10.1016/j.euroneuro.2014.08.005

15 Mayberg HS. Limbic-cortical dysregulation: A proposed model of depression. *J. Neuropsychiatry Clin. Neurosci.* 1997;**9**:471–81. doi:10.1176/jnp.9.3.471

16 Sheline YI, Price JL, Yan Z, *et al.* Resting-state functional MRI in depression unmasks increased connectivity between networks via the dorsal nexus. *Proc Natl Acad Sci U S A* 2010;**107**:11020–5. doi:10.1073/pnas.1000446107

17 Mulders PC, van Eijndhoven PF, Schene AH, Beckmann CF, Tendolkar I. Resting-state

For peer review only

For peer review only - http://bmjopen.bmj.com/site/about/guidelines.xhtml

functional connectivity in major depressive disorder: A review. *Neurosci Biobehav Rev.* 2015;**56**:330-44. doi: 10.1016/j.neubiorev.2015.07.014.

18 Lan MJ, Chhetry BT, Liston C, Mann JJ, Dubin M. Transcranial magnetic stimulation of left dorsolateral prefrontal cortex induces brain morphological changes in regions associated with a treatment resistant major depressive episode: an exploratory analysis. *Brain Stimul*. 2016;**9**:577-83. doi: 10.1016/j.brs.2016.02.011.

19 McGrath CL, Kelley ME, Holtzheimer PE *et al.* Toward a neuroimaging treatment selection biomarker for major depressive disorder. *JAMA Psychiatry*. 2013;**70**:821-9. doi: 10.1001/jamapsychiatry.2013.143.

20 Drysdale AT, Grosenick L, Downar J, *et al.* Resting-state connectivity biomarkers define neurophysiological subtypes of depression. *Nat Med* 2017;**23**:28–38. doi:10.1038/nm.4246

21 Iwabuchi SJ, Auer DP, Lankappa ST, *et al.* Baseline effective connectivity predicts response to repetitive transcranial magnetic stimulation in patients with treatment-resistant depression. *Eur Neuropsychopharmacol* 2019;**29**:681–90. doi:10.1016/j.euroneuro.2019.02.012

22 Iwabuchi SJ, Raschke F, Auer DP, *et al.* Targeted transcranial theta-burst stimulation alters fronto-insular network and prefrontal GABA. *Neuroimage* 2017;**146**:395–403. doi:10.1016/j.neuroimage.2016.09.043

23 Liston C, Chen AC, Zebley BD, *et al.* Default mode network mechanisms of transcranial magnetic stimulation in depression. *Biol Psychiatry* 2014;**76**:517–26. doi:10.1016/j.biopsych.2014.01.023

24 First MB, Williams JBW, Karg RS, *et al*. Structured clinical interview for DSM-5—Research version (SCID-5 for DSM-5, research version; SCID-5-RV). Arlington, Va, American Psychiatric Association 2015

25 American Psychiatric Association. *Diagnostic and statistical manual of mental disorders (DSM-5. 5th edn).* Arlington, VA: American Psychiatric Association Publishing, 2013

26 Fava M. Diagnosis and definition of treatment-resistant depression. *Biol Psychiatry* 2003;**53**:649–59. doi:10.1016/S0006-3223(03)00231-2

27. Williams JBW, Kobak KA, Bech P, *et al.* The GRID-HAMD: standardization of the Hamilton Depression Rating Scale. *Int Clin Psychopharmacol* 2008;**23**:120–9. doi:10.1097/YIC.0b013e3282f948f5

28. Bernstein DP, Fink L, Handelsman L, *et al.* Initial reliability and validity of a new retrospective measure of child abuse and neglect. *Am J Psychiatry* 1994;**151**:1132–6. doi:10.1176/ajp.151.8.1132

29 Beck AT, Steer RA, Brown GK. Manual for Beck Depression Inventory-II. San Antonio, TX: Psychological Corporation, 1996

30 Kroenke K, Spitzer RL, Williams JBW. The PHQ-9: Validity of a brief depression severity measure. *J Gen Intern Med* 2001;**16**:606–13. doi:10.1046/j.1525-1497.2001.016009606.x

31 Spitzer RL, Kroenke K, Williams JBW, *et al.* A brief measure for assessing generalized anxiety disorder: The GAD-7. *Arch Intern Med* 2006;**166**:1092–7. doi:10.1001/archinte.166.10.1092

32 Mundt JC, Marks IM, Shear MK, et al. The Work and Social Adjustment Scale: A simple measure of impairment in functioning. Br J Psychiatry 2002;180:461–4. doi:10.1192/bjp.180.5.461

For peer review only - http://bmjopen.bmj.com/site/about/guidelines.xhtml

33 Van Hout B, Janssen MF, Feng Y-F et al. Interim Scoring for the EQ-5D-5L: Mapping the EQ-5D-5L to EQ-5D-3L. Value Sets. Value in Health 2012; 15: 708-715. doi: 10.1016/j.jval.2012.02.008

34 Rush AJ, Trivedi MH, Ibrahim HM, et al. The 16-item Quick Inventory of Depressive Symptomatology (QIDS) Clinician Rating (QIDS-C) and Self-Report (QIDS-SR): A psychometric evaluation in patients with chronic major depression. Biol Psychiatry 2003; 54: 573-583. doi: 10.1016/s0006-3223(02)01866-8

35 Beecham J, Knapp MR. Costing psychiatric intervention. In: Thornicroft GJ ed. Measuring mental health needs. Gaskell, 2001

36 Demyttenaere K, Desaiah D, Petit C, et al. Patient-assessed versus physician-assessed disease severity and outcome in patients with nonspecific pain associated with major depressive disorder. Prim Care Companion J Clin Psychiatry 2009;11:8–15. doi:10.4088/pcc.08m00670

37 Zimmerman M, Martinez JH, Young D, *et al.* Severity classification on the Hamilton depression rating scale. *J Affect Disord* 2013;**150**:384–8. doi:10.1016/j.jad.2013.04.028

38 McAllister-Williams RH, Anderson IM, Finkelmeyer A, *et al.* Antidepressant augmentation with metyrapone for treatment-resistant depression (the ADD study): A double-blind, randomised, placebo-controlled trial. *The Lancet Psychiatry* 2016;**3**:117–27. doi:10.1016/S2215-0366(15)00436-8

39 Curtis, L., Burns, A. (2017) Unit Costs of Health and Social Care 2017, Personal Social Services Research Unit, University of Kent, Canterbury. https://doi.org/10.22024/UniKent/01.02/65559

40 Hoch JS, Briggs AH, Willan AR. Something old, something new, something borrowed, something blue: a framework for the marriage of health econometrics and cost-effectiveness analysis. *Health Econ* 2002;**11**:415–30. doi:10.1002/hec.678

For peer review only

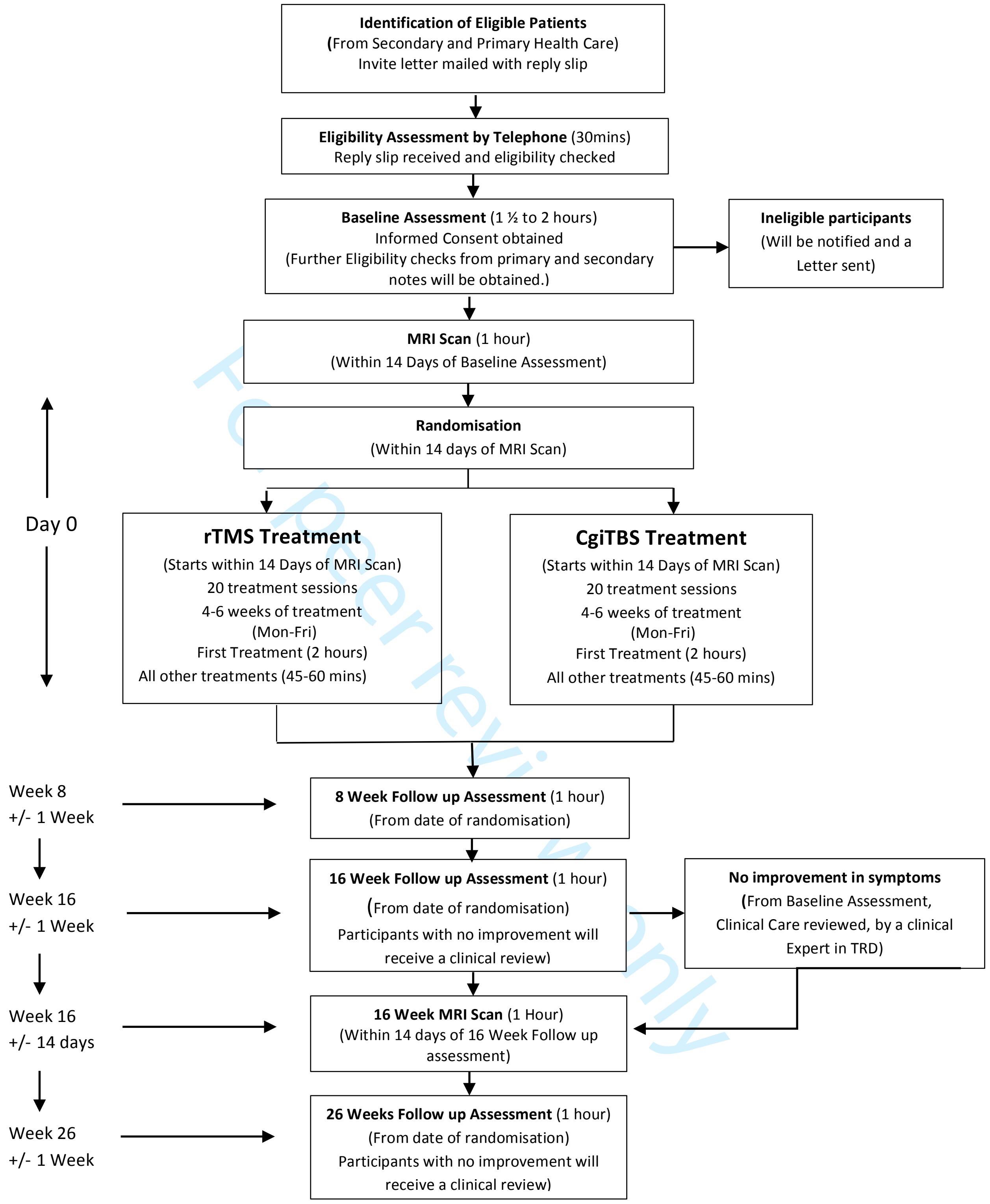

Figure 1. Study Flow Chart

For peer review only - http://bmjopen.bmj.com/site/about/guidelines.xhtml

Appendix 1: Assessment of degree of treatment resistance.

The definition and assessment of the degree of treatment resistance depression (TRD) in the BRIGhTMIND study is operationalised using the Massachusetts General Hospital (MGH) TRD scoring system (Fava et al, 2003). This assesses the number of previous biological treatments for depression, given as an adequate treatment trial, in the current episode of depression. The use of psychological treatments is not assessed by this tool because it can be challenging to obtain reliable accounts of structured psychotherapy from participant's accounts (Lobban et al, 2017). To date, the relationship between non-response to psychotherapy and response to TMS has not been investigated. This is in contrast to evidence that the degree of treatment resistance to biological treatments may moderate treatment outcome to TMS (Lee et al, 2012). The use of, and response to, psychological therapy is recorded separately to the MGH TRD score in the BRIGhTMIND study for use as an exploratory variable in relation to predictors of response.

The MGH TRD score is based on one point being scored for every different antidepressant prescribed at the minimum effective dose for a minimum of 6 weeks, without response. An extra 0.5 points is scored if the trial has been at least 10 weeks and if the dose of the antidepressant has been optimised to a defined level. A further 0.5 can be scored if the antidepressant has been augmented by a second drug. Any course of electroconvulsive therapy lasting a minimum of 4 treatment sessions is given a score of 3 points.

The MGH TRD scoring tool used in BRIGhTMIND is an updated version of that published by Fava et al. in 2003, with medications not available at that time added and ones that are no longer, or where never, available in the UK removed (see supplementary table 1). Specifically, the tricyclic antidepressants amoxapine, desipramine, maprotiline and protriptyline, and the "other" antidepressant nefazadone, that were included in the scoring table in Fava et al. (2003), were removed. The tricyclic antidepressants dosulepin (75 and 150mg) lofepramine (140 and 210mg), monoamine oxidase inhibitor moclobemide (300 and 600mg) and "other" antidepressants reboxetine (8 and 12mg), agomelatine (25 and 50mg) and vortioxetine (10 and 20mg) were added with minimum and optimised doses indicated in brackets. These doses were based on the various drugs' Summary of Product Characteristics (SPC) and consensus of the clinicians involved in the setup of BRIGhTMIND.

In the scoring table published by Fava et al. (2003), the definition of which drugs could be counted towards augmenting an antidepressant is vague, simply presenting examples: "e.g., buspirone (Buspar), lithium, psychostimulants such as methylphenidate (Ritalin), atypical antipsychotics such as olanzapine (Zyprexa). It was decided that more precise guidance should be provided. The drugs included are based on consensus of BRIGhTMIND clinicians and British Association for Psychopharmacology (BAP) Guidelines (Cleare et al, 2015). All first and second line BAP options were included (lithium, aripiprazole, quetiapine, risperidone, olanzapine and tri-iodothyronine (T3)). BAP guidelines also include mirtazapine augmentation of another antidepressant. Because of the diverse range of antidepressant combinations sometimes used in clinical practice, "mirtazapine" was broadened to "any second antidepressant". The two remaining drugs specifically mentioned by Fava et al. (2003), buspirone and methylphenidate were retained (i.e. all drugs specifically mentioned

For peer review only

by Fava et al. are included). Two extra drugs were then added by consensus on the basis of awareness of their use in the centres involved in the BRIGhTMIND study: modafinil and pramipexole. These both have some RCT evidence supporting their use as augmentation agents (Goss et al. 2013; Tundo et al. 2019).

In BRIGhTMIND each patient is scored on the adapted MGH TRD scoring system. The inclusion criterion for the RCT is a score of 2 points or more. This is broadly in line with the conventional definition of TRD as being failure to respond to two adequate courses of different antidepressants (Brown et al. 2019): failure to respond to two different antidepressants prescribed at the minimum dose for 6 weeks each would score 2 X 1 point. However, it should be noted that dose optimisation and a treatment trial of a single antidepressant could lead to the same score (1 + 0.5 + 0.5 points).

Scoring the MGD TRD scale is dependent on having information regarding the patient's past treatments during this current episode. In BRIGhTMIND, this is established through a combination of interview with the patient and examination of primary and secondary care case notes. Patients are interviewed using timeline follow back techniques to identify when the current episode of depression started. However, the MGH TRD scores are very susceptible to error due to mis-remembered details and inadequate, inaccurate or inaccessible case notes. Rather than focusing on the specific MGH TRD score, patients in BRIGhTMIND are allocated to three categories of degree of treatment resistance: low, medium and high. These are defined on the basis of data collected in a previous RCT in patients with TRD, the ADD study (McAllister-Williams et al. 2016), by scores of 2-3.5, 4-6 and ≥6.5 respectively. If there is any concern that there is incomplete data on past treatments, then a patient is allocated to the high resistance group.

The specific guidance on using the MGH TRD scoring system in BRIGhTMIND is as follows:

1. The scale assesses the degree of treatment resistance in the CURRENT EPISODE. For some patients with long histories of depression it can be difficult to determine the beginning of an episode. Count a new episode of depression from the end of any period of substantially better mood for a minimum of 2 months.
2. Confirm if ANY antidepressants have been taken (not just prescribed) in the current episode. If not, then the patient is excluded.
3. If the participant has taken any antidepressants in the current episode then collect information regarding WHAT has been taken (all psychotropics), at what DOSE and for how LONG at the minimum dose or greater. Then, using supplementary table 1:
    a. in column A, tick an antidepressant the patient has taken at the minimum dose for at least 6 weeks during THIS episode of depression.
    b. for antidepressants ticked in column A, put another tick in column B if the treatment continued for at least 10 weeks.
    c. tick column C if the patient has taken the drug at a dose equal to or greater than the maximum dosage listed for that medication. *(There is no extra score for doses higher than the maximum)*
    d. If the patient has been prescribed any of the drugs listed here (taken for at least 4 weeks) during the same time period to boost the antidepressant effect, write the name in column D.

For peer review only

i. **NB** – if an antidepressant combination is used, then only score for one of these with the second antidepressant being the augmentation agent. For example if a patient on venlafaxine has mirtazapine added, put a tick in the venlafaxine row and write 'mirtazapine' in Column D. Don't tick the Mirtazapine row (unless this was also used in monotherapy).

ii. **NB** – augmentation agents should in theory be used at minimum effective doses. However, there is a lack of consensus as to what these should be. If in any doubt seek a view of the local Principal Investigators or Chief Investigator.

4. Add the scores:
   a. For each antidepressant add a score to column E. This is +1 for the antidepressant, +0.5 if used for at least 10 weeks (at minimum dose or above), +0.5 if the Maximum dose or greater was used and +0.5 for EACH augmentation agent used.
   b. If the patient has received ECT in this episode, then add 3 into column E on the ECT row at the bottom
   c. Calculate the total MGH TRD score adding up all scores in column E.

For peer review only

Supplementary table 1

Adapted Massachusetts General Hospitals Staging Method to classify treatment resistant depression for the BRIGhTMIND Study.

Has the patient received any treatment with ANTIDEPRESSANT medications since the beginning of THIS CURRENT episode or period of depression?

1 = Yes 2 = No ☐ If NO – Exclude from Study

If Yes

1) On the Grid below in column A, tick an antidepressants the patient has taken at the minimum dose for at least 6 weeks during THIS episode of depression.
2) For antidepressants ticked in column A, put another tick in column B if the treatment continued for at least 10 weeks.
3) Tick column C if the patient has taken the drug at a dose equal to or greater than the maximum dosage listed for that medication. *(There is no extra score for doses higher than the maximum.)*
4) If the patient has been prescribed any of the drugs listed here during the same time period to boost the antidepressant effect, write the name in column D, *Amisulpride, Aripiprazole, Buspirone, Lithium, Methylphenidate, Modafinil, Olanzapine, Pramipexole, Quetiapine, Risperidone, triiodothyronine (T3), and any second Antidepressant.*

**If ticked score**			1	0.5		0.5	0.5	
			A	B		C	D	
	**Generic name**	**Min dose (mg/day)**	**At least 6 wks**	**At least 10 wks**	**Max dose (mg/day)**	**Equal or greater**	**Name of drug added to augment this antidepressant**	**Score**
**Tricyclic antidepressants**	Doxepin	150			250			
	Clomipramine	150			250			
	Amitriptyline	150			250			
	Nortriptyline	75			125			
	Trimipramine	150			250			
	Imipramine	150			250			
	Dosulepin	75			150			
	Lofepramine	140			210			
**MAOIs**	Isocarboxazid	30			60			
	Phenelzine	45			90			
	Tranylcypromine	30			60			
	Moclobemide	300			600			

SSRIs	Fluvoxamine	50			150			
	Paroxetine	20			60			
	Fluoxetine	20			60			
	Sertraline	50			150			
	Citalopram	20			60			
	Escitalopram	10			30			
SNRI	Venlafaxine	125			250			
	Duloxetine	60			100			
Other	Trazodone	300			600			
	Bupropion	300			450			
	Mirtazapine	15			45			
	Reboxetine	8			12			
	Agomelatin	25			50			
	Vortioxetine	10			20			
Did the patient receive electroconvulsive treatment (ECT) during the current episode (Score 3)?								
							Total MGH score	

Appendix 2 Studies on Cognition, depression and effective connectivity

Background

Depression is associated with marked impairments in attention and executive function (Austin et al., 2001; McDermott and Ebmeier, 2009). Part of these impairments could be explained by increased intraindividual variability (IIV) in attentional performance – patients with depression show a skewed distribution of response times on sustained attention tasks, with an increased frequency of slow responses consistent with attentional lapses (Gallagher et al., 2015). This can be analysed by decomposing the distribution of response times into a Gaussian component and an exponential component (Schmiedek et al., 2007) – Gallagher et al. (2015) found the time constant of the exponential component to be a more sensitive measure for differentiating patients from controls than simple mean reaction times.

It may be that attentional lapses reflect interference from brain areas involved in internally directed mental activity and rumination (the so-called "default mode network" of areas, DMN) on areas involved in executive function (the "frontoparietal network" of areas, FPN). Previous work has found that executive function in non-clinical populations is related to the degree of anticorrelation between the activities of the FPN and DMN (Kelly et al., 2008; Keller et al., 2015), suggesting that the ability to separate the activities of these networks may be critical to task performance. One of the key findings of a meta-analysis of resting-state fMRI studies in patients with depression was an increase in connectivity between the FPN and the DMN, suggesting a breakdown of this separability (Kaiser et al., 2015).

It is unclear the extent to which attentional and executive impairments are independent components of the pathophysiology of depression or a secondary consequence of impairments in mood (Bora et al., 2013; Rock et al., 2014). Independence could be demonstrated in a dissociation between improvements in mood and improvements in cognition during treatment. Cheng et al. (2016) provide preliminary evidence for such a dissociation with a selective improvement in executive function in a group receiving intermittent TBS compared to those receiving continuous TBS or a combination of intermittent and continuous TBS.

Methods

Embedded within the BRIGhTMIND study, we will explore correlations between IIV on the THINC-it battery of cognitive tests (measured with the method of Gallagher et al., 2015) and resting-state functional connectivity between the FPN and the DMN. Separate correlations using baseline measures of each variable and using change in each variable from baseline to 16 weeks will be calculated. Analyses will be performed without unblinding TMS treatment allocation group. We predict a positive correlation between IIV and FPN-DMN functional connectivity at baseline, and a positive correlation between change in IIV and change in FPN-DMN connectivity. We will explore the mediating effects of low mood (measured with the QIDS-SR, Rush et al., 2013), and improvements in mood, on these relationships. Additionally, we will examine the longitudinal relationship between mood and IIV in a within-subject design using unblinded QIDS-SR and THINC-it data, to ascertain whether increased IIV is a state or trait property of depression.

For peer review only

References

Austin MP, Mitchell P, Goodwin GM. Cognitive deficits in depression: possible implications for functional neuropathology. *Br J Psychiatry* 2001; **178**:200-6.

Bora E, Harrison BJ, Yücel M, et al. Neurocognitive intra-individual variability in mood disorders: effects on attentional response time distributions. *Psychol Med* 2015; **45**: 2985-97.

Brown S, Rittenbach K, Cheung S, McKean G, MacMaster FP, Clement F. Current and Common Definitions of Treatment-Resistant Depression: Findings from a Systematic Review and Qualitative Interviews. *Can. J. Psychiatry* 2019; **64**: 380–387. https://doi.org/10.1177/0706743719828965

Cheng C-M, Juan C-H, Chen M-H, et al. Different forms of prefrontal theta burst stimulation for executive function of medication-resistant depression: evidence from a randomized sham-controlled study. *Prog Neuro-Psychopharmacol Biol Psychiatry* 2016; **66**: 35-40.

Cleare A, Pariante C, Young A, et al. Evidenced-based guidelines for treating depressive disorders with antidepressants: A revision of the 2008 British Association Psychopharmacology guidelines. J Psychopharmacol 2015;29:459-525.

Fava M. Diagnosis and definition of treatment-resistant depression. *Biol Psychiatry* 2003;**53**:649–59. doi:10.1016/S0006-3223(03)00231-2.

Gallagher P, Nilsson J, Finkelmeyer A et al. Neurocognitive intra-individual variability in mood disorders: effects on attentional response time distributions. *Psychol Med.* 2015;**45**:2985-97. doi: 10.1017/S0033291715000926.

Goss AJ, Kaser M, Costafreda SG, Sahakian BJ, Fu CH. Modafinil augmentation therapy in unipolar and bipolar depression: a systematic review and meta-analysis of randomized controlled trials. *J Clin Psychiatry* 2013; **74**:1101-7. doi: 10.4088/JCP.13r08560.

Kaiser RH, Andrews-Hanna JR, Wager TD, Pizzagalli DA. Large-Scale network dysfunction in major depressive disorder: a meta-analysis of resting-state functional connectivity. *JAMA Psychiatry.* 2015;**72**:603-11. doi: 10.1001/jamapsychiatry.2015.0071.

Keller JB, Hedden T, Thompson TW, Anteraper SA, Gabrieli JD, Whitfield-Gabrieli S. Resting-state anticorrelations between medial and lateral prefrontal cortex: association with working memory, aging, and individual differences. *Cortex.* 2015;**64**:271-80. doi: 10.1016/j.cortex.2014.12.001

Kelly AM, Uddin LQ, Biswal BB et al. Competition between functional brain networks mediates behavioral variability. *Neuroimage.* 2008;**39**:527-37.

Lee JC, Blumberger DM, Fitzgerald PB, Daskalakis ZJ, Levinson AJ. The role of transcranial magnetic stimulation in treatment-resistant depression: a review. Curr Pharm Des. 2012;18:5846-52.

Lobban F, Dodd AL, Sawczuk AP et al. Assessing Feasibility and Acceptability of Web-Based Enhanced Relapse Prevention for Bipolar Disorder (ERPonline): A Randomized Controlled Trial. J Med Internet Res. 2017 Mar 24;19(3):e85. doi: 10.2196/jmir.7008.

McAllister-Williams RH, Anderson IM, Finkelmeyer A, et al. Antidepressant augmentation with metyrapone for treatment-resistant depression (the ADD study): A double-blind, randomised, placebo-controlled trial. *Lancet Psychiatry* 2016; **3**:117–27. doi:10.1016/S2215-0366(15)00436-8

For peer review only

McDermott LM, Ebmeier KP. A meta-analysis of depression severity and cognitive function. *J Affect Disord.* 2009;**119**:1-8. doi: 10.1016/j.jad.2009.04.022

Rock PL, Roiser JP, Riedel WJ, Blackwell AD. Cognitive impairment in depression: a systematic review and meta-analysis. *Psychol Med.* 2014;**44**:2029-40. doi: 10.1017/S0033291713002535.

Rush AJ, Trivedi MH, Ibrahim HM, et al. The 16-item Quick Inventory of Depressive Symptomatology (QIDS) Clinician Rating (QIDS-C) and Self-Report (QIDS-SR): A psychometric evaluation in patients with chronic major depression. *Biol Psychiatry* 2003; **54**: 573-583. doi: 10.1016/s0006-3223(02)01866.

Schmiedek F, Oberauer K, Wilhelm O, et al. Individual differences in components of reaction time distributions and their relations to working memory and intelligence. *J Exp Psychol General* 2007; **136**: 414-429.

Tundo A, de Filippis R, De Crescenzo F. Pramipexole in the treatment of unipolar and bipolar depression. A systematic review and meta-analysis. *Acta Psychiatr Scand* 2019; **140**:116-125. doi: 10.1111/acps.13055.

For peer review only

SPIRIT
STANDARD PROTOCOL ITEMS: RECOMMENDATIONS FOR INTERVENTIONAL TRIALS

SPIRIT 2013 Checklist: Recommended items to address in a clinical trial protocol and related documents*

Section/item	Item No	Description	Addressed on page number
**Administrative information**			
Title	1	Descriptive title identifying the study design, population, interventions, and, if applicable, trial acronym	_______1________
Trial registration	2a	Trial identifier and registry name. If not yet registered, name of intended registry	_______2________
	2b	All items from the World Health Organization Trial Registration Data Set	ISRCTN19674644
Protocol version	3	Date and version identifier	______18_________
Funding	4	Sources and types of financial, material, and other support	______18________
Roles and responsibilities	5a	Names, affiliations, and roles of protocol contributors	____1,17,18_____
	5b	Name and contact information for the trial sponsor	______18_________
	5c	Role of study sponsor and funders, if any, in study design; collection, management, analysis, and interpretation of data; writing of the report; and the decision to submit the report for publication, including whether they will have ultimate authority over any of these activities	______18_________
	5d	Composition, roles, and responsibilities of the coordinating centre, steering committee, endpoint adjudication committee, data management team, and other individuals or groups overseeing the trial, if applicable (see Item 21a for data monitoring committee)	______14_________

For peer review only - http://bmjopen.bmj.com/site/about/guidelines.xhtml

**Introduction**			
Background and rationale	6a	Description of research question and justification for undertaking the trial, including summary of relevant studies (published and unpublished) examining benefits and harms for each intervention	____3,4________
	6b	Explanation for choice of comparators	____3,4________
Objectives	7	Specific objectives or hypotheses	_____4,7_______
Trial design	8	Description of trial design including type of trial (eg, parallel group, crossover, factorial, single group), allocation ratio, and framework (eg, superiority, equivalence, noninferiority, exploratory)	______5_______
**Methods: Participants, interventions, and outcomes**			
Study setting	9	Description of study settings (eg, community clinic, academic hospital) and list of countries where data will be collected. Reference to where list of study sites can be obtained	______5_______
Eligibility criteria	10	Inclusion and exclusion criteria for participants. If applicable, eligibility criteria for study centres and individuals who will perform the interventions (eg, surgeons, psychotherapists)	______5,6______
Interventions	11a	Interventions for each group with sufficient detail to allow replication, including how and when they will be administered	______6,7______
	11b	Criteria for discontinuing or modifying allocated interventions for a given trial participant (eg, drug dose change in response to harms, participant request, or improving/worsening disease)	_____6,7_______
	11c	Strategies to improve adherence to intervention protocols, and any procedures for monitoring adherence (eg, drug tablet return, laboratory tests)	_____6,7_______
	11d	Relevant concomitant care and interventions that are permitted or prohibited during the trial	______7_______
Outcomes	12	Primary, secondary, and other outcomes, including the specific measurement variable (eg, systolic blood pressure), analysis metric (eg, change from baseline, final value, time to event), method of aggregation (eg, median, proportion), and time point for each outcome. Explanation of the clinical relevance of chosen efficacy and harm outcomes is strongly recommended	______8,9, 11___
Participant timeline	13	Time schedule of enrolment, interventions (including any run-ins and washouts), assessments, and visits for participants. A schematic diagram is highly recommended (see Figure)	______9, Figure 1

For peer review only - http://bmjopen.bmj.com/site/about/guidelines.xhtml

Sample size	14	Estimated number of participants needed to achieve study objectives and how it was determined, including clinical and statistical assumptions supporting any sample size calculations	___13_______
Recruitment	15	Strategies for achieving adequate participant enrolment to reach target sample size	_____8________

Methods: Assignment of interventions (for controlled trials)

Allocation:

Sequence generation	16a	Method of generating the allocation sequence (eg, computer-generated random numbers), and list of any factors for stratification. To reduce predictability of a random sequence, details of any planned restriction (eg, blocking) should be provided in a separate document that is unavailable to those who enrol participants or assign interventions	______10_______
Allocation concealment mechanism	16b	Mechanism of implementing the allocation sequence (eg, central telephone; sequentially numbered, opaque, sealed envelopes), describing any steps to conceal the sequence until interventions are assigned	______10_______
Implementation	16c	Who will generate the allocation sequence, who will enrol participants, and who will assign participants to interventions	______10_______
Blinding (masking)	17a	Who will be blinded after assignment to interventions (eg, trial participants, care providers, outcome assessors, data analysts), and how	______13_______
	17b	If blinded, circumstances under which unblinding is permissible, and procedure for revealing a participant's allocated intervention during the trial	______13_______

Methods: Data collection, management, and analysis

Data collection methods	18a	Plans for assessment and collection of outcome, baseline, and other trial data, including any related processes to promote data quality (eg, duplicate measurements, training of assessors) and a description of study instruments (eg, questionnaires, laboratory tests) along with their reliability and validity, if known. Reference to where data collection forms can be found, if not in the protocol	____8-10______
	18b	Plans to promote participant retention and complete follow-up, including list of any outcome data to be collected for participants who discontinue or deviate from intervention protocols	_______14_____

Data management	19	Plans for data entry, coding, security, and storage, including any related processes to promote data quality (eg, double data entry; range checks for data values). Reference to where details of data management procedures can be found, if not in the protocol	Statistical Analysis Plan___________
Statistical methods	20a	Statistical methods for analysing primary and secondary outcomes. Reference to where other details of the statistical analysis plan can be found, if not in the protocol	__10-12_______
	20b	Methods for any additional analyses (eg, subgroup and adjusted analyses)	___11-12_______
	20c	Definition of analysis population relating to protocol non-adherence (eg, as randomised analysis), and any statistical methods to handle missing data (eg, multiple imputation)	___10-12_______
**Methods: Monitoring**			
Data monitoring	21a	Composition of data monitoring committee (DMC); summary of its role and reporting structure; statement of whether it is independent from the sponsor and competing interests; and reference to where further details about its charter can be found, if not in the protocol. Alternatively, an explanation of why a DMC is not needed	_____14,17_____
	21b	Description of any interim analyses and stopping guidelines, including who will have access to these interim results and make the final decision to terminate the trial	_____None______
Harms	22	Plans for collecting, assessing, reporting, and managing solicited and spontaneously reported adverse events and other unintended effects of trial interventions or trial conduct	_____14________
Auditing	23	Frequency and procedures for auditing trial conduct, if any, and whether the process will be independent from investigators and the sponsor	___By sponsor__
**Ethics and dissemination**			
Research ethics approval	24	Plans for seeking research ethics committee/institutional review board (REC/IRB) approval	______14_______
Protocol amendments	25	Plans for communicating important protocol modifications (eg, changes to eligibility criteria, outcomes, analyses) to relevant parties (eg, investigators, REC/IRBs, trial participants, trial registries, journals, regulators)	___No changes_

For peer review only - http://bmjopen.bmj.com/site/about/guidelines.xhtml

Consent or assent	26a	Who will obtain informed consent or assent from potential trial participants or authorised surrogates, and how (see Item 32)	_______8_______
	26b	Additional consent provisions for collection and use of participant data and biological specimens in ancillary studies, if applicable	_____N/A_________
Confidentiality	27	How personal information about potential and enrolled participants will be collected, shared, and maintained in order to protect confidentiality before, during, and after the trial	___Under GCP and Data Protection Act___
Declaration of interests	28	Financial and other competing interests for principal investigators for the overall trial and each study site	_____19__________
Access to data	29	Statement of who will have access to the final trial dataset, and disclosure of contractual agreements that limit such access for investigators	_Statistical Analysis Plan_
Ancillary and post-trial care	30	Provisions, if any, for ancillary and post-trial care, and for compensation to those who suffer harm from trial participation	___None_________
Dissemination policy	31a	Plans for investigators and sponsor to communicate trial results to participants, healthcare professionals, the public, and other relevant groups (eg, via publication, reporting in results databases, or other data sharing arrangements), including any publication restrictions	_____14__________
	31b	Authorship eligibility guidelines and any intended use of professional writers	___17,18________
	31c	Plans, if any, for granting public access to the full protocol, participant-level dataset, and statistical code	Publication of protocol paper and Statistical Analysis Plan
**Appendices**			
Informed consent materials	32	Model consent form and other related documentation given to participants and authorised surrogates	From sponsor
Biological specimens	33	Plans for collection, laboratory evaluation, and storage of biological specimens for genetic or molecular analysis in the current trial and for future use in ancillary studies, if applicable	______N/A_______

For peer review only - http://bmjopen.bmj.com/site/about/guidelines.xhtml

*It is strongly recommended that this checklist be read in conjunction with the SPIRIT 2013 Explanation & Elaboration for important clarification on the items. Amendments to the protocol should be tracked and dated. The SPIRIT checklist is copyrighted by the SPIRIT Group under the Creative Commons "Attribution-NonCommercial-NoDerivs 3.0 Unported" license.

For peer review only

For peer review only - http://bmjopen.bmj.com/site/about/guidelines.xhtml
